# PRDX6 Prevents NNMT Ubiquitination and Degradation as a Nonenzymatic Mechanism to Promote Ovarian Cancer Progression

**DOI:** 10.1002/advs.202416484

**Published:** 2025-01-30

**Authors:** Xingyun Wu, Li Luo, Mao Wang, Lixia Dong, Jiawu Fan, Yan Zeng, Sijia Li, Kui Wang

**Affiliations:** ^1^ West China School of Basic Medical Sciences & Forensic Medicine State Key Laboratory of Biotherapy West China Hospital Sichuan University Chengdu 610041 P. R. China; ^2^ Center for Reproductive Medicine Department of Gynecology and Obstetrics West China Second University Hospital Sichuan University Chengdu 610041 P. R. China; ^3^ Key Laboratory of Birth Defects and Related Diseases of Women and Children (Sichuan University) Ministry of Education Chengdu 610041 P. R. China

**Keywords:** NNMT, nonenzymatic function, ovarian cancer, PRDX6, ubiquitin‐mediated degradation

## Abstract

Cancer cells cope with oxidative stress for their proliferation and metastasis by equipping antioxidant systems, among which the antioxidant enzymes peroxiredoxins (PRDXs) play crucial roles. However, whether PRDXs exhibit nonenzymatic functions remains unclear. Here, it is shown that the 1‐cysteine PRDX (PRDX6) upregulates nicotinamide *N*‐methyltransferase (NNMT) to promote the growth and metastasis of ovarian cancer cells, independently of PRDX6's enzymatic activities. Mechanistically, PRDX6 interacts with NNMT to prevent its binding to the E3 ubiquitin ligase tripartite‐motif protein 56 (TRIM56), leading to the inhibition of NNMT ubiquitination at lysine 23 and 210 and suppression of subsequent proteasomal degradation. In addition, PRDX6‐mediated NNMT upregulation activates mitogen‐activated protein kinase (MAPK) signaling, thereby promoting the growth and metastasis of ovarian cancer cells. Notably, PRDX6 overexpression is associated with higher NNMT protein levels in human ovarian cancer tissues and is predictive of poor prognosis of ovarian cancer patients. Overall, the findings illustrate a critical oncogenic mechanism of the antioxidant enzyme PRDX6 in promoting ovarian cancer progression beyond its enzymatic mechanisms.

## Graphical abstract

This study unravels a crucial oncogenic mechanism of the antioxidant enzyme PRDX6 in ovarian cancer beyond its enzymatic mechanisms. PRDX6 interacts with and upregulates NNMT via preventing TRIM56‐mediated ubiquitination and proteasomal degradation, thereby activating MAPK signaling and promoting ovarian cancer progression.

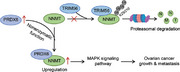

## 1 Introduction

Ovarian cancer is the most lethal gynecologic malignancy worldwide, with ≈3 14 000 new cases and 2 07 000 new deaths annually.^[^ ^1^ ^]^ Due to the lack of specific symptoms and inadequacy of effective early diagnosis, many patients are initially diagnosed at advanced stages, leading to the 5‐year survival rate less than 50%.^[^ ^2^ ^]^ The standard therapy for ovarian cancer is debulking surgery combined with platinum‐based chemotherapy.^[^ ^3^ ^]^ Although this therapeutic regimen confers a survival advantage, most patients ultimately suffer recurrence due to peritoneal metastasis or chemoresistance.^[^ ^4^ ^]^ Therefore, it is imperative to decipher the molecular mechanisms underlying the pathogenesis and progression of ovarian cancer, which will facilitate the identification of promising therapeutic targets and development of potential treatment strategies.

Recently, accumulating evidence has revealed that, apart from their conventional enzymatic activities, metabolic enzymes exhibit moonlighting functions to regulate various instrumental biological events for cancer progression.^[^ ^5^ ^]^ On one hand, some metabolic enzymes can gain noncanonical enzymatic functions. For instance, metabolic enzymes including pyruvate kinase M2 (PKM2), phosphoenolpyruvate carboxykinase 1 (PCK1), fructose‐2,6‐bisphosphatase 4 (PFKFB4), and ketohexokinase‐A, function as protein kinases to phosphorylate various protein substrates, resulting in the regulation of gene transcription enabling cancer development.^[^ ^6^ ^]^ Phosphoglycerate dehydrogenase (PHGDH) and lactate dehydrogenase A (LDHA) were found to gain noncanonical enzymatic activities in the nucleus to influence gene transcription and promote cancer cell growth.^[^ ^7^ ^]^ On the other hand, several metabolic enzymes also harbor nonenzymatic functions. For example, PHGDH, LDHA, glutamine synthetase, and thymidine kinase 1 (TK1) can regulate the activity, stability, or function of other proteins through protein‐protein interaction to support cancer progression, independently of their metabolic enzyme activities.^[^ ^8^ ^]^ Deciphering the moonlighting functions (including noncanonical enzymatic activities and nonenzymatic functions) of metabolic enzymes or other enzymes will deepen our understanding of the mechanisms of cancer progression from a new perspective, and lead to the development of novel therapeutic strategies in cancer.

The peroxiredoxins (PRDXs) family is one of the major antioxidant enzymes and is frequently overexpressed in cancer. Given their peroxidase activities, PRDXs play crucial roles in maintaining intracellular redox homeostasis and regulating redox signaling to promote cancer progression.^[^ ^9^ ^]^ Among them, peroxiredoxin 6 (PRDX6) is a unique member of PRDXs family due to its lack of a resolving cysteine (1‐cysteine PRDX), as well as gain of two noncanonical enzymatic activities, phospholipase A_2_ (PLA_2_) activity and lysophosphatidylcholine acyltransferase (LPCAT) activity.^[^ ^10^ ^]^ It has been reported that PRDX6 can remove lipid reactive oxygen species (LOOH) and thereby inhibit ferroptosis of lung cancer cells through noncanonical PLA_2_ activity.^[^ ^11^ ^]^ In addition, PRDX6 was also found to exhibit LPCAT activity for the repair of peroxidized cell membrane phospholipids.^[^ ^12^ ^]^ Currently, the oncogenic role of PRDX6 has been widely demonstrated in various cancers, including breast cancer, liver cancer and lung cancer.^[^ ^13^ ^]^ However, the tumor‐promoting mechanisms of PRDX6 are mainly attributable to its conventional peroxidase activity or noncanonical PLA_2_ activity, whether PRDX6 has nonenzymatic function in cancer remains unclear.

In this study, we show a nonenzymatic mechanism underlying PRDX6's tumor‐promoting role in ovarian cancer. PRDX6 nonenzymatically interacts with nicotinamide *N*‐methyltransferase (NNMT) to prevent the binding of NNMT with the E3 ubiquitin ligase tripartite‐motif protein 56 (TRIM56), leading to the inhibition of NNMT ubiquitination and proteasomal degradation. PRDX6‐mediated NNMT upregulation thus promotes the growth and metastasis of ovarian cancer cells by activating the mitogen‐activated protein kinase (MAPK) pathway.

## 2 Results

### 2.1 PRDX6 is Upregulated in Ovarian Cancer and Promotes the Growth and Metastasis of Ovarian Cancer Cells

**Figure 1:**
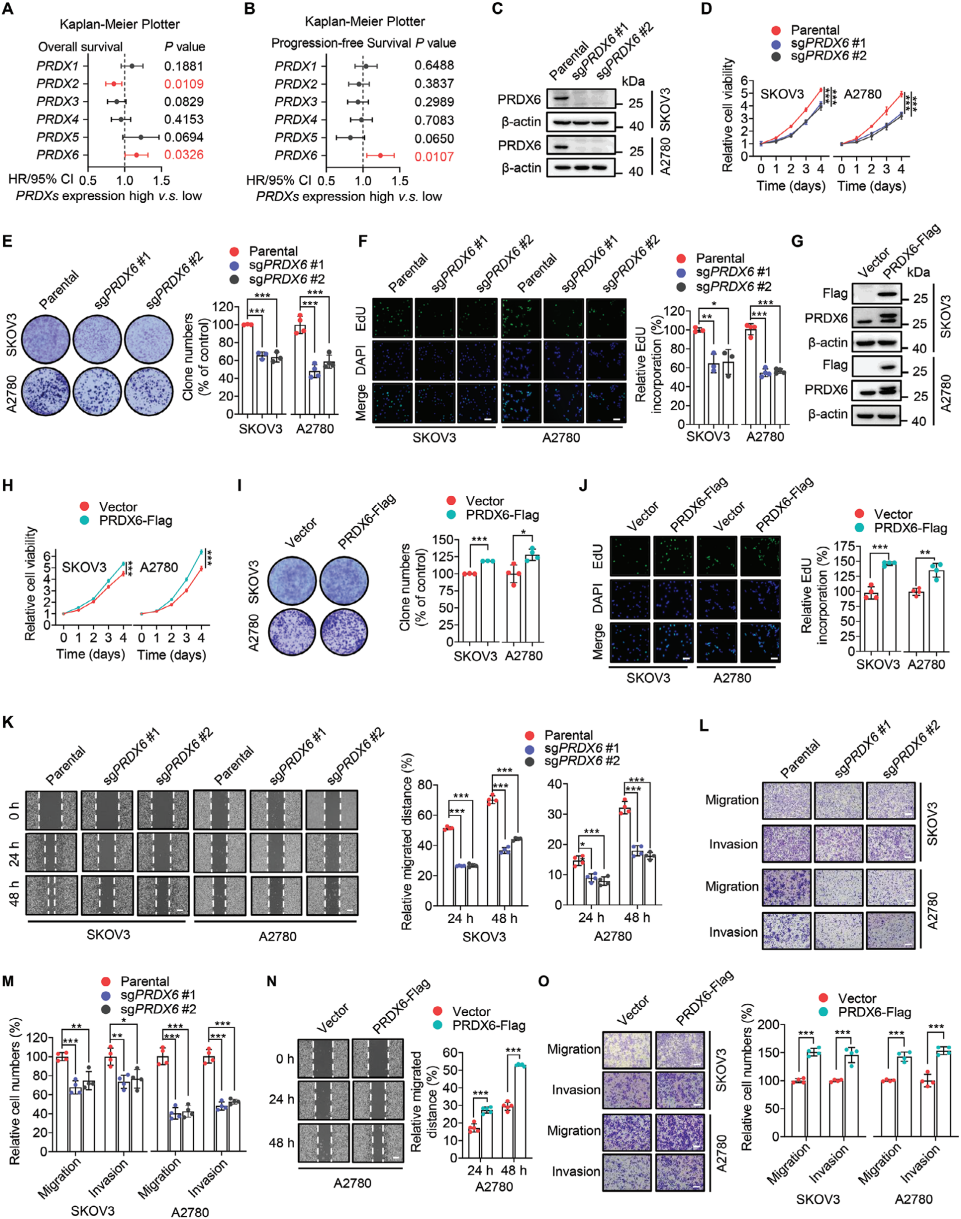
PRDX6 promotes the growth and metastasis of ovarian cancer cells. A) Forest plot showing the hazard ratios (HR) and *P* values for overall (A) and progression‐free (B) survival analysis based on the expression of different PRDXs in human ovarian cancer tissues using the Kaplan‐Meier Plotter database. C) Immunoblotting analysis of PRDX6 in SKOV3 and A2780 cells with or without *PRDX6* knockout (KO). D–F) Cell viability (D), colony formation (E), and 5‐ethynyl‐2′‐deoxyuridine (EdU) (F) assays of SKOV3 and A2780 cells with or without *PRDX6* KO. G) Immunoblotting analysis of PRDX6 in SKOV3 and A2780 cells with or without PRDX6 overexpression. H–J) Cell viability (H), colony formation (I), and EdU (J) assays of SKOV3 and A2780 cells with or without PRDX6 overexpression. K) Wound healing assay of SKOV3 and A2780 cells with or without *PRDX6* KO. L,M) Transwell assay examining the migration and invasion of SKOV3 and A2780 cells with or without *PRDX6* KO. N) Wound healing assay of A2780 cells with or without PRDX6 overexpression. O) Transwell assay examining the migration and invasion of SKOV3 and A2780 cells with or without PRDX6 overexpression. Scale bar, 100 µm. Results are representative of at least three independent experiments. Data are presented as mean ± SD. ^*^ *P* < 0.05, ^**^ *P* < 0.01, ^***^ *P* < 0.001.

To investigate the role of PRDXs in the malignant progression of ovarian cancer, we analyzed the correlation between PRDXs expression and survival of ovarian cancer patients using the Kaplan‐Meier Plotter online tool. Among the six PRDX members, only the expression of PRDX6 was positively associated with worse overall survival and progression‐free survival of ovarian cancer patients (**Figure** **1**A,B; Figure S1A,B, Supporting Information). We then analyzed the TCGA, GTEx, and GSE26712 datasets, and found that the mRNA level of PRDX6 was significantly upregulated in human ovarian cancer tissues compared with normal ovarian tissues (Figure S1C,D, Supporting Information). These observations were confirmed by immunoblotting analysis, which showed that PRDX6 was highly expressed in ovarian cancer cells (SKOV3, A2780, and OVCAR5 cells) compared with normal ovarian epithelial cells (IOSE80 and HOSEpiC cells; Figure S1E, Supporting Information). In addition, PRDX6 mRNA level was significantly higher in patients with stage III‐IV ovarian cancer compared to those with stage I‐II disease (Figure S1F, Supporting Information). Notably, the frequency of increased mRNA level of PRDX6 in ovarian cancer tissues ranked top among all PRDX members by analyzing the cBioportal database (Figure S1G, Supporting Information). These data suggest that PRDX6 is upregulated in ovarian cancer and associated with poor prognosis of ovarian cancer patients.

We next asked how the PRDX6 mRNA level is increased in ovarian cancer. Genomic analysis of the TCGA database revealed a remarkable amplification of PRDX6 copy number in ovarian cancer tissues (Figure S1H, Supporting Information). In comparison with the PRDX6 diploid group, the mRNA and protein levels of PRDX6 were both elevated in copy number gain and amplification groups (Figure S1I,J, Supporting Information). In addition, the expression of PRDX6 was positively correlated with its copy number in ovarian cancer tissues (Figure S1K,L, Supporting Information). Taken together, these data demonstrate that PRDX6 is upregulated in ovarian cancer, at least in part, due to copy number gain and amplification.

Given the pathological role of PRDX6 in ovarian cancer, we sought to investigate the biological function of PRDX6 in ovarian cancer progression in cultured ovarian cancer cells. We generated *PRDX6* knockout (KO) ovarian cancer cells using CRISPR/Cas9 genome editing (Figure 1C) and then performed cell viability and colony formation assays to examine the effect of PRDX6 on the growth of ovarian cancer cells. As shown in Figure 1D,E, KO of *PRDX6* markedly inhibited the growth of SKOV3 and A2780 cells. The decreased growth of ovarian cancer cells caused by *PRDX6* KO was mainly attributed to impaired proliferation abilities as evidenced by 5‐ethynyl‐2′‐deoxyuridine (EdU) assay (Figure 1F), but not induction of apoptosis (Figure S2A,B, Supporting Information). In contrast, ectopic expression of PRDX6 profoundly promoted the growth and proliferation of ovarian cancer cells (Figure 1G–J). We further examined the impact of PRDX6 on the metastatic potential of ovarian cancer cells by performing wound healing assay and transwell assay. *PRDX6* KO profoundly diminished the migration and invasion of SKOV3 and A2780 cells (Figure 1K–M), whereas overexpression of PRDX6 obviously increased the migration and invasion of ovarian cancer cells (Figure 1N,O). Collectively, these results indicate that PRDX6 promotes the growth, migration, and invasion of ovarian cancer cells in vitro.

### 2.2 The Nonenzymatic Function of PRDX6 is Required for Ovarian Cancer Progression

**Figure 2:**
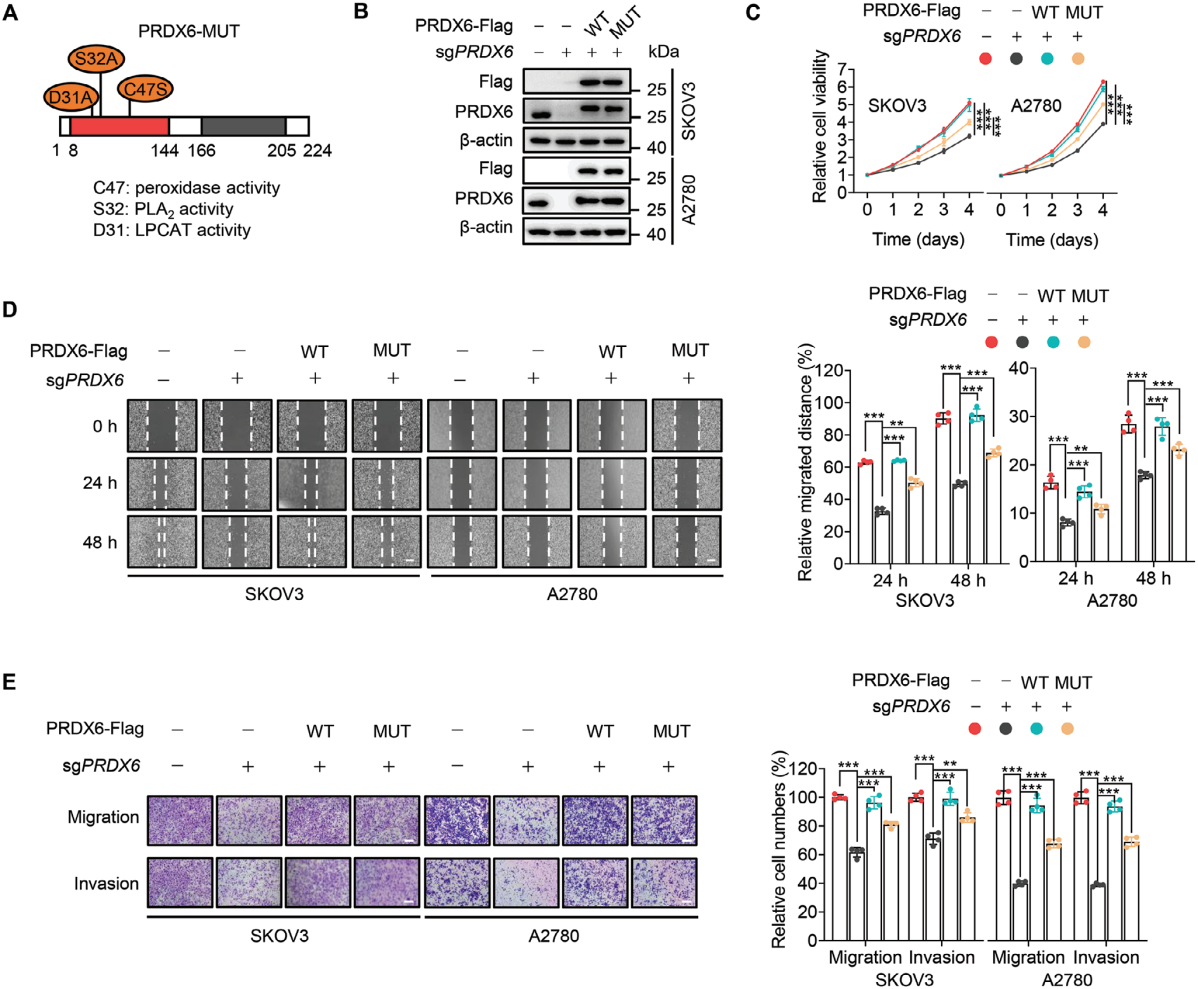
The nonenzymatic function of PRDX6 is required for ovarian cancer progression. A) Schematic representation of the enzymatically dead mutant of PRDX6 (C47S/S32A/D31A, namely PRDX6‐MUT). B) Immunoblotting analysis of endogenous and exogenous PRDX6 in *PRDX6* knockout (KO) ovarian cancer cells re‐introducing PRDX6‐WT or PRDX6‐MUT. C,D) Cell viability (C) and wound healing assays (D) of *PRDX6* KO ovarian cancer cells re‐introducing PRDX6‐WT or PRDX6‐MUT. E) Transwell assay examining the migration and invasion of *PRDX6* KO ovarian cancer cells re‐introducing PRDX6‐WT or PRDX6‐MUT. Scale bar, 100 µm. Results are representative of at least three independent experiments. Data are presented as mean ± SD. ^*^ *P* < 0.05, ^**^ *P* < 0.01, ^***^ *P* < 0.001.

PRDX6 is a trifunctional enzyme with peroxidase, PLA_2_, and LPCAT activities.^[^ ^10a^ ^]^ To determine whether PRDX6 promotes ovarian cancer progression via its enzymatic activities, we generated an enzymatically dead mutant of PRDX6 (C47S/S32A/D31A, namely PRDX6‐MUT), with all three enzymatic activities abrogated (**Figure** **2**A; Figure S3A,B, Supporting Information).^[^ ^10^, ^12^, ^14^ ^]^ Wild‐type PRDX6 (PRDX6‐WT) or PRDX6‐MUT was reconstituted approximately equal to physiological level into *PRDX6* KO cells (Figure 2B). Consistent with the observation in Figure 1D, the growth of SKOV3 and A2780 cells was significantly suppressed by *PRDX6* KO, and re‐introducing PRDX6‐WT completely restored this growth inhibition effect (Figure 2C). Interestingly, reconstituted expression of the enzymatically dead mutant PRDX6‐MUT could still partially, but markedly, rescue *PRDX6* KO‐induced growth inhibition of ovarian cancer cells (Figure 2C). Similar rescuing phenotypes were also observed for the migration and invasion of *PRDX6* KO ovarian cancer cells (Figure 2D,E). In addition, withangulatin A, a covalent PRDX6 inhibitor which inhibited the peroxidase and PLA_2_ activities of PRDX6 (Figure S3C,D, Supporting Information),^[^ ^15^ ^]^ profoundly suppressed the growth, migration, and invasion of ovarian cancer cells (Figure S3E,F, Supporting Information). These data indicate that, in addition to its enzymatic activities, PRDX6 promotes the growth, migration, and invasion of ovarian cancer cells via nonenzymatic mechanisms.

### 2.3 PRDX6 Interacts with and Upregulates NNMT Independently of PRDX6's Enzymatic Activities

**Figure 3:**
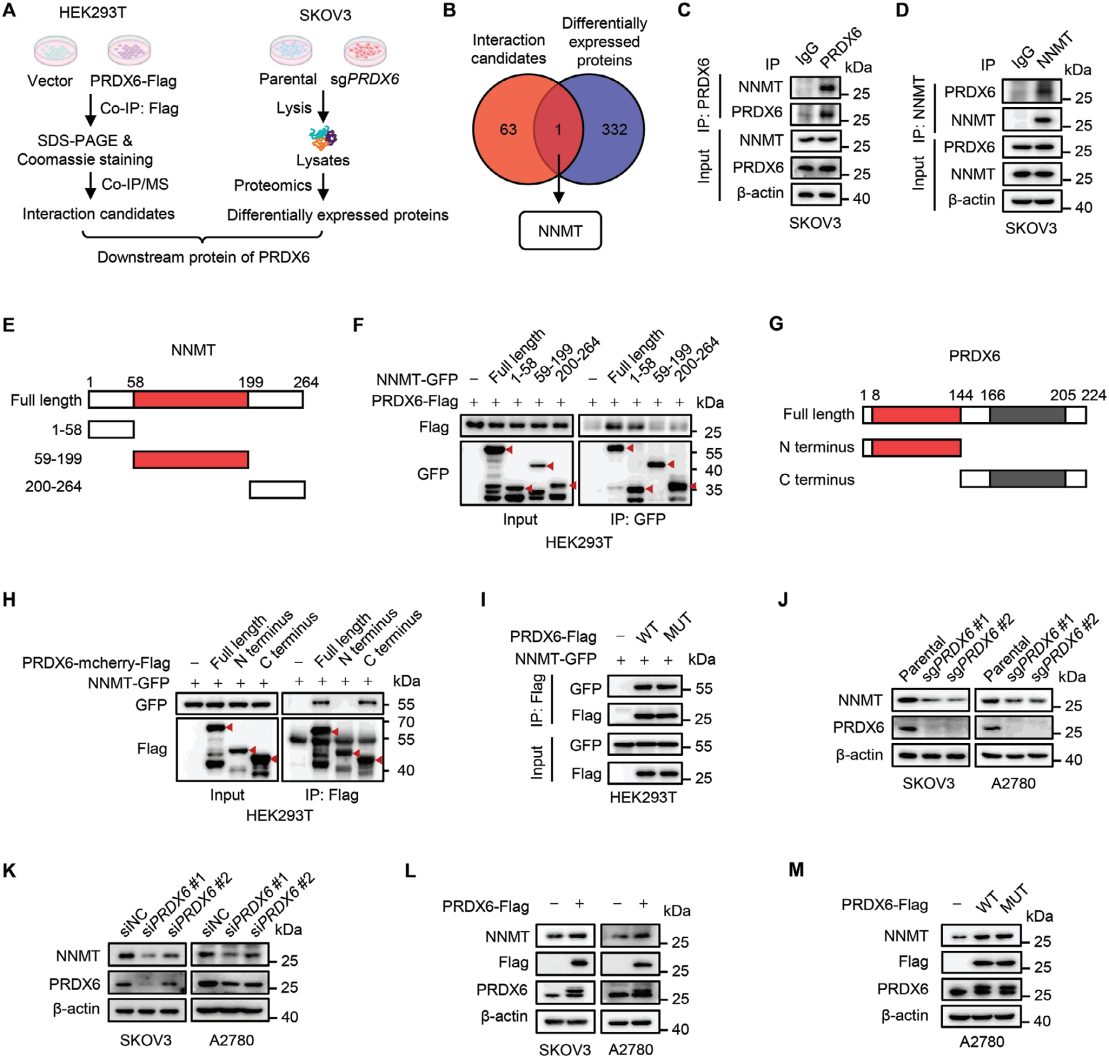
PRDX6 interacts with and upregulates NNMT independently of PRDX6's enzymatic activities. A) Schematic of the screening workflow for identifying the direct downstream targets of PRDX6. B) Venn diagram showing overlap of the results from the co‐immunoprecipitation (co‐IP)/mass spectrometry (MS) and TMT‐based quantitative proteomics analysis. C,D) Reciprocal co‐IP analysis of endogenous PRDX6 and NNMT in SKOV3 cells. E) Schematic representation of full length and different truncation mutations of NNMT. F) Full length or indicated truncation mutations of NNMT‐GFP were co‐expressed with PRDX6‐Flag in HEK293T cells. NNMT‐GFP was immunoprecipitated with GFP antibody, followed with immunoblotting analysis of PRDX6‐Flag using Flag antibody. G) Schematic representation of full length and different truncation mutants of PRDX6. H) Full length or indicated truncation mutations of PRDX6‐mcherry‐Flag were co‐expressed with NNMT‐GFP in HEK293T cells. PRDX6‐mcherry‐Flag was immunoprecipitated with Flag beads, followed with immunoblotting analysis of NNMT‐GFP using GFP antibody. I) Co‐IP analysis of PRDX6‐WT or PRDX6‐MUT with NNMT‐GFP in HEK293T cells. J,K) Immunoblotting analysis of NNMT in SKOV3 and A2780 cells with *PRDX6* knockout (J) or knockdown (K). L) Immunoblotting analysis of NNMT in SKOV3 and A2780 cells with PRDX6 overexpression. M) Immunoblotting analysis of NNMT in A2780 cells overexpressing PRDX6‐WT or PRDX6‐MUT. Results are representative of at least three independent experiments.

To investigate the nonenzymatic mechanism underlying PRDX6's tumor‐promoting function in ovarian cancer, we performed an integrated screening strategy to identify the direct downstream targets of PRDX6. Potential PRDX6‐interacting proteins were pulled down and identified using co‐immunoprecipitation (co‐IP) assay coupled with mass spectrometry (MS) analysis in PRDX6‐overexpressing cells (**Figure** **3**A, left; Table S1, Supporting Information). In parallel, a TMT‐based quantitative proteomics analysis was conducted to profile the differentially expressed proteins in *PRDX6* KO cells (Figure 3A, right; Table S2, Supporting Information). By analyzing the integrated screening data, we identified NNMT as the only protein that interacts with and is regulated by PRDX6 (Figure 3B). Co‐IP analysis showed that exogenous PRDX6 interacted with NNMT in HEK293T cells with ectopic expression of PRDX6‐FLAG and NNMT‐HA (Figure S4A,B, Supporting Information). The binding of endogenous PRDX6 with NNMT in SKOV3 and A2780 cells was further confirmed by reciprocal co‐IP analysis (Figure 3C,D; Figure S4C,D, Supporting Information). We then mapped the domains of PRDX6 and NNMT required for their interaction by expressing different truncation mutations of PRDX6 and NNMT in HEK293T cells. As a result, the N‐terminal domain (1‐58aa) of NNMT was required for its binding with PRDX6 (Figure 3E,F), and the C‐terminal domain (145‐224aa) of PRDX6 was necessary for its binding with NNMT (Figure 3G,H). Given that the key amino acids (C47, S32, and D31) required for the three catalytic activities of PRDX6 were all located at the N‐terminal domain, we presumed its interaction with NNMT is independent of PRDX6's enzymatic activities. As expected, compared with PRDX6‐WT, the enzymatically dead mutation (PRDX6‐MUT) had a negligible effect on the interaction between PRDX6 and NNMT (Figure 3I). Together, these results demonstrate that PRDX6 interacts with NNMT independently of PRDX6's enzymatic activities.

The above quantitative proteomics analysis revealed downregulation of NNMT protein level in response to *PRDX6* KO in ovarian cancer cells. In support of this observation, immunoblotting analysis revealed an obvious decrease in NNMT protein level upon *PRDX6* KO or knockdown (KD) by siRNA in ovarian cancer cells (Figure 3J,K). Moreover, the ectopic expression of PRDX6 markedly elevated NNMT protein level in ovarian cancer cells (Figure 3L). Notably, enforced expression of PRDX6‐MUT led to a comparable increase of NNMT protein level in comparison with PRDX6‐WT (Figure 3M). Overall, these data indicate that PRDX6 interacts with and upregulates NNMT independently of PRDX6 enzymatic activities.

### 2.4 PRDX6 Inhibits TRIM56‐Mediated NNMT Ubiquitination at Lysine 23 and 210 to Prevent NNMT Proteasomal Degradation in Ovarian Cancer Cells

**Figure 4:**
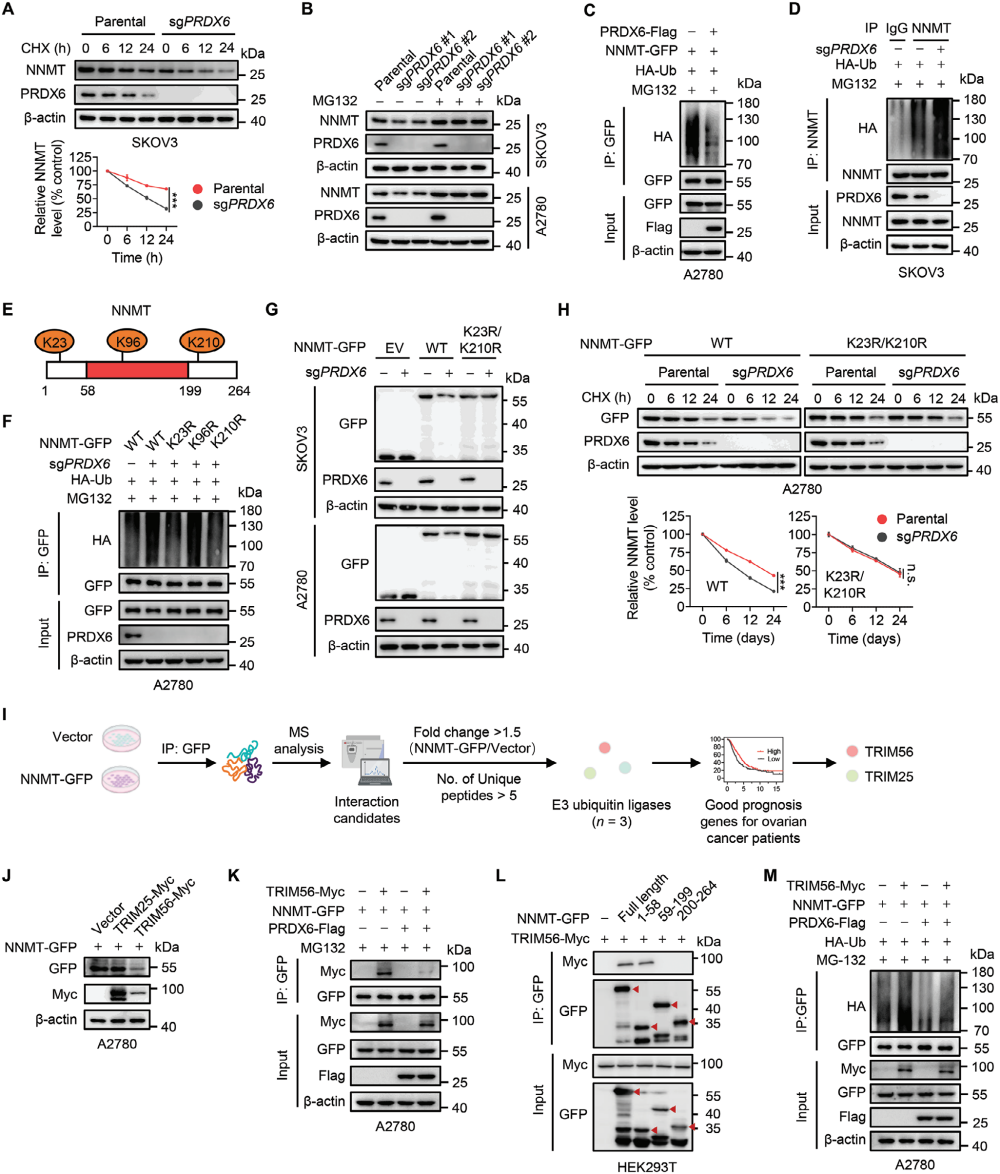
PRDX6 inhibits TRIM56‐mediated NNMT ubiquitination at lysine 23 and 210 to prevent NNMT proteasomal degradation in ovarian cancer cells. A) Immunoblotting analysis of NNMT in *PRDX6* knockout (KO) SKOV3 and A2780 cells treated with or without cycloheximide (CHX) for the indicated time. Quantitation of NNMT protein level based on band intensity was shown (bottom). B) Immunoblotting analysis of NNMT in *PRDX6* KO SKOV3 and A2780 cells treated with or without MG132. C) NNMT‐GFP and HA‐Ub were co‐expressed with or without PRDX6‐Flag in A2780 cells. After MG132 treatment, immunoprecipitation (IP) was performed using GFP antibody, followed by immunoblotting analysis with indicated antibodies. D) HA‐Ub plasmid was transfected in SKOV3 cells with or without *PRDX6* KO. After MG132 treatment, IP was performed using IgG or NNMT antibody, followed by immunoblotting analysis with indicated antibodies. E) Three potential lysine ubiquitination sites (K23, K96, and L210) of NNMT identified by mass spectrometry analysis. F) Wild‐type (WT) or indicated KR mutants of NNMT‐GFP was co‐expressed with HA‐Ub in A2780 cells with or without *PRDX6* KD. After MG132 treatment, IP was performed using GFP antibody, followed by immunoblotting analysis with indicated antibodies. G) WT or K23R/K210R double mutant of NNMT‐GFP was expressed in SKOV3 and A2780 cells with or without *PRDX6* KD. Immunoblotting analysis was performed with indicated antibodies. GFP empty vector was used a negative control. H) NNMT‐GFP WT or K23R/K210R mutant plasmid was transfected into A2780 cells with or without *PRDX6* KD, followed by CHX treatment for the indicated time. Immunoblotting analysis was performed with indicated antibodies. Quantitation of NNMT protein level based on band intensity was shown (bottom). I) Schematic of the screening workflow to identify the potential E3 ubiquitin ligase for NNMT ubiquitination. J) Immunoblotting analysis of NNMT‐GFP in A2780 cells expressing TRIM56 or TRIM25. K) NNMT‐GFP was co‐expressed with TRIM56‐Myc and/or PRDX6‐Flag in A2780 cells. After MG132 treatment, IP was performed using GFP antibody, followed by immunoblotting analysis with indicated antibodies. L) Full length or indicated truncation mutations of NNMT‐GFP were co‐expressed with TRIM56‐Myc in HEK293T cells. NNMT‐GFP was immunoprecipitated with GFP antibody, followed with immunoblotting analysis of TRIM56‐Myc using Myc antibody. M) NNMT‐GFP and HA‐Ub were co‐expressed with TRIM56‐Myc and/or PRDX6‐Flag in A2780 cells. After MG132 treatment, IP was performed using GFP antibody, followed by immunoblotting analysis with indicated antibodies. Results are representative of at least three independent experiments. Data are presented as mean ± SD. ^*^ *P* < 0.05, ^**^ *P* < 0.01, ^***^ *P* < 0.001. n.s., not significant.

Given that PRDX6 interacts with NNMT and upregulates NNMT protein level, we speculated that PRDX6 upregulates NNMT by regulating protein stability. We therefore determined the half‐life of NNMT to evaluate its protein stability using cycloheximide (CHX), a protein synthesis inhibitor. Compared with parental cells, KO of *PRDX6* significantly reduced the half‐life of NNMT protein (**Figure** **4**A), whereas ectopic expression of PRDX6 markedly prolonged the half‐life of NNMT protein in ovarian cancer cells (Figure S5A, Supporting Information), suggesting that PRDX6 enhances the protein stability of NNMT. In addition, treatment with the proteasome inhibitor MG132 obviously increased NNMT protein level, and rescued *PRDX6* KO‐mediated decrease of NNMT protein level in ovarian cancer cells (Figure 4B). These observations suggest that PRDX6 upregulates the NNMT protein level probably by preventing its ubiquitin‐proteasome degradation. To determine whether PRDX6 suppresses the ubiquitination of NNMT, we co‐expressed NNMT‐GFP, PRDX6‐Flag, and HA‐ubiquitin (HA‐Ub) in HEK293T and A2780 cells, followed by MG132 treatment. Ectopic expression of PRDX6 markedly decreased the ubiquitination of exogenous NNMT‐GFP in HEK293T (Figure S5B, Supporting Information) and A2780 cells (Figure 4C). Whereas the level of NNMT‐GFP ubiquitination was prominently elevated by *PRDX6* KO in SKOV3 cells (Figure S5C, Supporting Information). These observations were further supported by the increased ubiquitination of endogenous NNMT protein in *PRDX6* KO cells compared with parental SKOV3 cells (Figure 4D). Collectively, these data demonstrate that PRDX6 stabilizes and upregulates NNMT protein levels by inhibiting its ubiquitin‐mediated degradation in ovarian cancer cells.

To identify the ubiquitination sites of NNMT, ubiquitinated NNMT‐Flag protein was immunoprecipitated for LC‐MS/MS analysis following the displayed workflow (Figure S5D, Supporting Information). Three lysine residues (K23, K96, and K210) were identified as potential ubiquitination sites of NNMT (Figure 4E). To determine which lysine residue is involved in PRDX6‐mediated inhibition of NNMT ubiquitination, we generated NNMT‐GFP mutants by replacing these lysine residues with arginine (K23R, K96R, or K210R) respectively, and then transfected these mutant constructs into *PRDX6* KO A2780 cells. By performing an in vivo ubiquitination assay, we found that KO of *PRDX6* obviously elevated the ubiquitination of wild‐type NNMT‐GFP. However, either K23R or K210R, but not K96R, of NNMT‐GFP profoundly countered *PRDX6* KO‐mediated increase of NNMT ubiquitination (Figure 4F). We then mutated both lysine 23 and 210 residues of NNMT‐GFP to arginine simultaneously (K23R/K210R), and found the K23R/K210R double mutation of NNMT markedly impeded the reduction of NNMT protein level caused by *PRDX6* KO in ovarian cancer cells (Figure 4G). Moreover, *PRDX6* KD significantly shortened the half‐life of wild‐type NNMT, but not K23R/K210R mutant (Figure 4H). The MS spectra indicating K23 and K210 ubiquitination of NNMT were shown (Figure S5E,F, Supporting Information). Overall, these results indicate that PRDX6 prevents NNMT ubiquitination at lysine 23 and 210, leading to the inhibition of proteasomal degradation and upregulation of NNMT in ovarian cancer cells.

We then employed a co‐IP assay coupled with MS analysis to identify the potential E3 ubiquitin ligase required for NNMT ubiquitination. As depicted in the screening workflow (Figure 4I), a total of 3 E3 ligases were identified among NNMT‐interacting proteins (Table S3, Supporting Information). Among them, only the expression of tripartite‐motif protein 25 (TRIM25) and TRIM56 predicted good survival for ovarian cancer patients (Figure 4I). Ectopic expression of TRIM56, but not TRIM25, significantly decreased the NNMT protein level in A2780 cells (Figure 4J). In addition, TRIM56 was capable of interacting with NNMT, and this interaction was disrupted by overexpressing PRDX6 (Figure 4K; Figure S6A, Supporting Information). However, we did not observe an interaction between TRIM56 and PRDX6 (Figure S6A, Supporting Information). Interestingly, the N‐terminal domain (1‐58aa) of NNMT was required for its binding with both TRIM56 (Figure 4L) and PRDX6 (Figure 3F). These observations suggest that PRDX6 can competitively bind to the N‐terminal domain of NNMT to abrogate the interaction of NNMT with TRIM56. To determine whether TRIM56 acts an E3 ubiquitin ligase for NNMT ubiquitination and degradation, we treated *TRIM56* KD cells with MG132 or CHX. Immunoblotting analysis showed that *TRIM56* KD‐mediated NNMT upregulation was abrogated in MG132‐treated cells (Figure S6B, Supporting Information). Meanwhile, KD of *TRIM56* profoundly prolonged the half‐life of NNMT protein (Figure S6C, Supporting Information). We further performed an in vivo ubiquitination assay, and found that enforced expression of TRIM56 prominently increased NNMT ubiquitination, whereas *TRIM56* KD decreased the ubiquitination level of NNMT protein (Figure S6D,E, Supporting Information). Interestingly, TRIM56‐mediated NNMT ubiquitination was markedly mitigated by PRDX6 overexpression (Figure 4M). Moreover, KD of *TRIM56* promoted the growth, migration, and invasion of ovarian cancer cells (Figure S6F,G, Supporting Information), which was consistent with a previous study.^[^ ^16^ ^]^ Ovarian cancer patients with higher TRIM56 expression exhibited longer survival time (Figure S6H, Supporting Information). Together, these data suggest that PRDX6 competitively interacts with NNMT to prevent TRIM56‐mediated NNMT ubiquitination, leading to NNMT upregulation in ovarian cancer cells.

### 2.5 PRDX6 Promotes the Growth and Metastasis of Ovarian Cancer Cells by Upregulating NNMT

**Figure 5:**
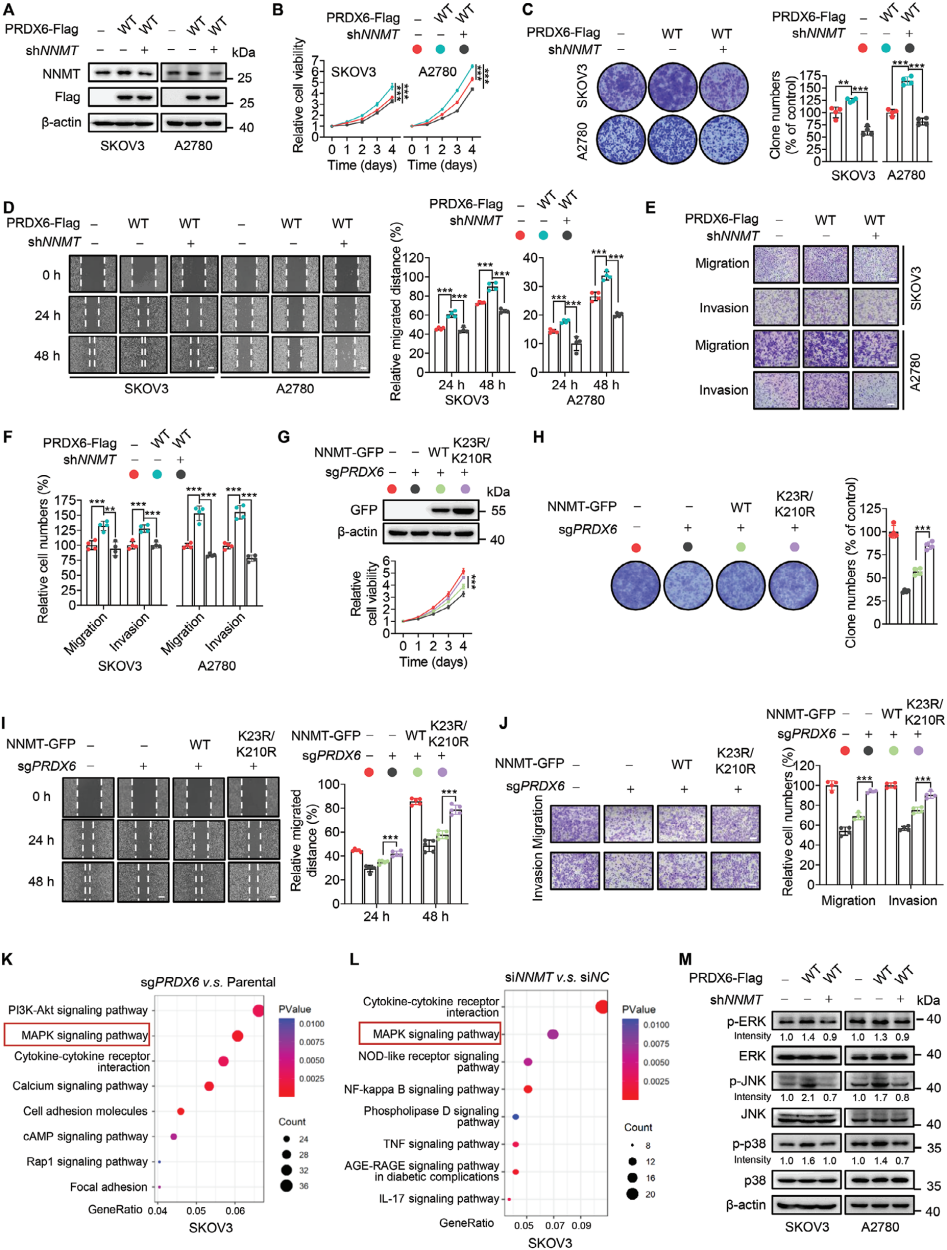
PRDX6 promotes the growth and metastasis of ovarian cancer cells by upregulating NNMT in vitro. A) Immunoblotting analysis of NNMT in PRDX6‐WT‐overexpressing SKOV3 and A2780 cells with or without *NNMT* knockdown (KD). B–D) Cell viability (B), colony formation (C), and wound healing (D) assays of PRDX6‐WT‐overexpressing SKOV3 and A2780 cells with or without *NNMT* KD. E,F) Transwell assay examining the migration and invasion of PRDX6‐WT‐overexpressing cells with or without *NNMT* KD. G) Immunoblotting analysis of NNMT‐GFP and cell viability assay in *PRDX6* knockout (KO) SKOV3 cells overexpressing wild‐type (WT) or K23R/K210R mutant of NNMT‐GFP. H,I) Colony formation (H) and wound healing (I) assays of *PRDX6* KO SKOV3 cells overexpressing NNMT‐GFP‐WT or K23R/K210R. J) Transwell assay examining the migration and invasion of *PRDX6* KO SKOV3 cells overexpressing NNMT‐GFP‐WT or K23R/K210R. K,L) Control and *PRDX6* KO (K) or *NNMT* KD (L) SKOV3 cells were subjected to RNA‐seq analysis. Differentially expressed genes were identified and subjected to Kyoto Encyclopedia of Genes and Genomes (KEGG) enrichment analysis. M) Immunoblotting analysis of MAPK signaling pathway using indicated antibodies in PRDX6‐WT‐overexpressing SKOV3 and A2780 cells with or without *NNMT* KD. The intensity of p‐ERK, p‐JNK, and p‐p38 was quantified using Image J software, and then normalized by β‐actin intensity. Scale bar, 100 µm. Results are representative of at least three independent experiments. Data are presented as mean ± SD. ^*^ *P* < 0.05, ^**^ *P* < 0.01, ^***^ *P* < 0.001.

Given that PRDX6 upregulates NNMT independently of its enzymatic activities, we next investigated whether NNMT upregulation contributes to PRDX6's nonenzymatic function in promoting ovarian cancer progression. As shown in Figure S7A–C (Supporting Information), *NNMT* KD significantly decreased the clone numbers, migration, and invasion of ovarian cancer cells. In addition, ovarian cancer patients with higher NNMT expression exhibited shorter overall and progression‐free survival time (Figure S7D,E, Supporting Information). These observations suggest a tumor‐promoting effect of NNMT in ovarian cancer. Interestingly, by knocking down *NNMT* in PRDX6‐WT‐overexpressing cells (**Figure** **5**A), we found that *NNMT* KD markedly abolished the promoting effect of PRDX6‐WT on the growth and proliferation of ovarian cancer cells, as evidenced by cell viability and colony formation assays (Figure 5B,C). Moreover, wound healing and transwell assays showed that PRDX6‐WT‐overexpressing cells exhibited increased migration and invasion, which could be obviously rescued by *NNMT* KD (Figure 5D–F). Similarly, *NNMT* KD could also compromise the tumor‐promoting effect of PRDX6‐MUT in ovarian cancer cells (Figure S7F–H, Supporting Information). To further confirm these observations, wild‐type NNMT or K23R/K210R double mutant was ectopically expressed in *PRDX6* KO SKOV3 cells. As shown in Figure 5G,H, enforced expression of K23R/K210R mutant of NNMT was blunt to *PRDX6* KO‐mediated downregulation, and significantly restored the decreased growth of *PRDX6* KO cells compared with wild‐type NNMT. Consistently, *PRDX6* KO‐mediated suppression of migration and invasion of ovarian cancer cells were markedly rescued by overexpressing NNMT‐K23R/K210R mutant (Figure 5I,J). Together, these results suggest that PRDX6 promotes the growth and metastasis of ovarian cancer cells by upregulating NNMT in vitro.

To determine the downstream signaling pathway of PRDX6‐mediated NNMT upregulation, we performed RNA‐seq analysis to profile the differentially expressed genes in response to *PRDX6* KO (Table S4, Supporting Information) or *NNMT* KD (Table S5, Supporting Information), respectively. Kyoto Encyclopedia of Genes and Genomes (KEGG) enrichment analysis revealed that both *PRDX6* KO and *NNMT* KD led to a significant alteration in the MAPK signaling pathway (Figure 5K,L). Therefore, we next investigated the effect of PRDX6‐mediated NNMT upregulation on MAPK signaling pathway. Immunoblotting analysis showed that overexpression of PRDX6 obviously promoted the activation of MAPK signaling pathway in ovarian cancer cells, as evidenced by increased phosphorylation levels of extracellular signal‐regulated kinase (ERK), c‐Jun N‐terminal kinase (JNK), and p38 (Figure 5M). The activation of MAPK pathway upon PRDX6 overexpression was profoundly abolished by KD of *NNMT* (Figure 5M). Previous studies have reported that NNMT, as a methyltransferase consuming the methyl donor *S*‐adenosylmethionine (SAM), could decrease the methylation level of protein phosphatase 2A (PP2A), resulting in the activation of MEK/ERK pathway in cancer cells.^[^ ^17^ ^]^ In this regard, we detected the levels of PP2A methylation and MEK phosphorylation in PRDX6‐overexpressing cells with or without *NNMT* KD. As shown in Figure S7I (Supporting Information), PRDX6 overexpression markedly reduced the methylation level of PP2A and elevated the phosphorylation level of MEK, which can both be rescued by *NNMT* KD, suggesting that PRDX6‐mediated NNMT upregulation activates MEK/ERK pathway by preventing PP2A methylation. Collectively, these results indicate that PRDX6‐mediated NNMT upregulation promotes the growth and metastasis of ovarian cancer cells by activating MAPK signaling.

**Figure 6:**
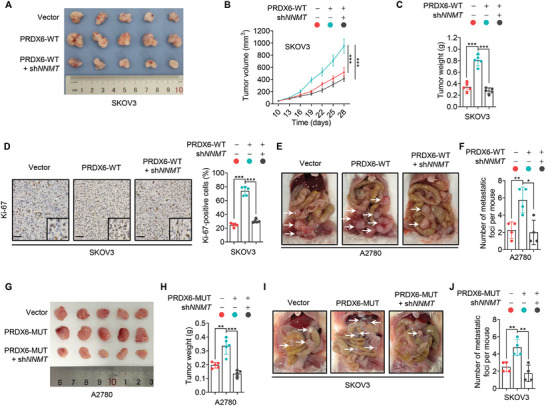
PRDX6 promotes ovarian cancer progression by upregulating NNMT in vivo. A–C) PRDX6‐WT‐overexpressing SKOV3 cells with or without *NNMT* knockdown (KD) were subcutaneously inoculated into immunodeficient mice. Tumor image (A), volume (B), and weight (C) were shown (*n* = 5). D) Representative image and quantitative analysis of immunohistochemical (IHC) staining for Ki‐67 in tumor xenografts in (A). Scale bar, 100 µm. E) PRDX6‐WT‐overexpressing A2780 cells with or without *NNMT* KD were intraperitoneally inoculated into immunodeficient mice. Representative image of metastatic nodules in the abdomen of mice was shown. F) Quantitative analysis of the number of metastatic tumors in (E) (*n* = 4). G,H) PRDX6‐MUT‐overexpressing A2780 cells with or without *NNMT* KD were subcutaneously inoculated into immunodeficient mice. Tumor image (G) and weight (H) were shown (*n* = 5). I) PRDX6‐MUT‐overexpressing SKOV3 cells with or without *NNMT* KD were intraperitoneally inoculated into immunodeficient mice. Representative image of metastatic nodules in the abdomen of mice was shown. J) Quantitative analysis of the number of metastatic tumors in (I) (*n* = 4). Data are presented as mean ± SD. ^*^ *P* < 0.05, ^**^ *P* < 0.01, ^***^ *P* < 0.001.

To investigate the effect of the PRDX6‐NNMT axis on the growth of ovarian cancer cells in vivo, we generated a mouse xenograft model by subcutaneously inoculating PRDX6‐overexpressing SKOV3 cells with or without *NNMT* KD into immunodeficient mice. Overexpression of PRDX6 significantly promoted the growth of tumor xenografts, as evidenced by increased tumor size, volume, and weight (**Figure** **6**A–C). However, this tumor‐promoting effect was greatly abolished by KD of *NNMT* (Figure 6A–C). Immunohistochemical (IHC) staining of Ki‐67 revealed a similar trend for the proliferation of tumor cells of xenografts (Figure 6D). In addition, a peritoneal metastatic xenograft model was also established to evaluate the effect of PRDX6‐NNMT axis on ovarian cancer metastasis in vivo. As shown in Figure 6E,F, PRDX6‐overexpressing tumors showed a marked increase of metastatic foci, which could be largely rescued by *NNMT* KD. Similarly, *NNMT* KD could also rescue the enhanced ovarian cancer growth (Figure 6G,H) and metastasis (Figure 6I,J) caused by PRDX6‐MUT overexpression in both subcutaneous and peritoneal metastatic xenograft mice models, respectively. Overall, these findings suggest that PRDX6 promotes the growth and metastasis of ovarian cancer cells in vivo by upregulating NNMT.

### 2.6 PRDX6 is Positively Correlated with NNMT in Ovarian Cancer Tissues and Predicts Poor Prognosis

**Figure 7:**
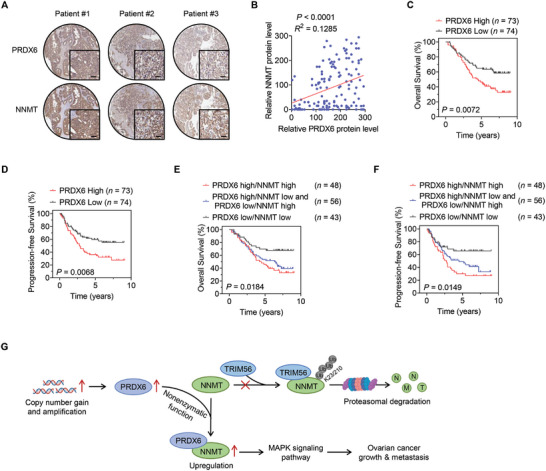
PRDX6 is positively correlated with NNMT in ovarian cancer tissues. A) Representative images of immunohistochemical (IHC) staining with PRDX6 and NNMT antibodies in ovarian cancer tissues (*n* = 147). Scale bar, 50 µm. B) Correlation analysis of the protein levels of PRDX6 and NNMT based on IHC staining scores. C,D) Overall (C) and progression‐free (D) survival analysis based on PRDX6 protein level in ovarian cancer patients (*n* = 147). E,F) Overall (E) and progression‐free (F) survival analysis based on combined PRDX6 and NNMT protein levels in ovarian cancer patients (*n* = 147). G) Schematic model for the nonenzymatic function of PRDX6 in promoting ovarian cancer progression.

We next interrogated the clinical relevance of PRDX6‐mediated NNMT upregulation in ovarian cancer using a tissue microarray containing 147 cases of human ovarian cancer. IHC staining revealed a positive correlation between PRDX6 and NNMT protein levels (**Figure** **7**A,B). Moreover, we evaluated the association of PRDX6 with the survival of ovarian cancer patients, and found that higher PRDX6 protein level was correlated with shorter overall and progression‐free survival, respectively (Figure 7C,D). Notably, ovarian cancer patients exhibiting high protein levels of both PRDX6 and NNMT showed worse overall and progression‐free survival (Figure 7E,F). Conversely, patients with low PRDX6 and NNMT proteins levels had the best survival outcome (Figure 7E,F). Taken together, these data indicate that PRDX6‐mediated NNMT upregulation can be potential biomarkers to predict the prognosis of ovarian cancer patients.

## 3 Discussion

Cancer progression is often associated with oxidative stress, requiring a higher antioxidant capacity to prevent oxidative damage and enable cell survival.^[^ ^18^ ^]^ The PRDXs family is an essential member of the antioxidant enzymes for maintaining intracellular redox homeostasis.^[^ ^19^ ^]^ Previous studies on PRDXs in cancer have focused on their conventional roles as antioxidant enzymes. Although the noncanonical PLA_2_ and LPCAT activities were also characterized for PRDX6,^[^ ^10^, ^12^ ^]^ whether PRDXs exhibit nonenzymatic function in cancer remains unknown. In this study, we reveal a nonenzymatic mechanism of PRDX6 in promoting ovarian cancer progression. In detail, PRDX6 competitively interacts with NNMT to prevent its binding to the E3 ubiquitin ligase TRIM56, resulting in the inhibition of ubiquitin‐mediated degradation of NNMT to activate MAPK signaling pathway and promote ovarian cancer progression (Figure 7G).

There is growing evidence demonstrating that cellular enzymes, especially metabolic enzymes, are capable of gaining moonlighting functions.^[^ ^5^ ^]^ For example, PKM2 can translocate to the nucleus to regulate gene expression by acting as an epigenetic regulator.^[^ ^20^ ^]^ TK1 can directly bind to protein arginine methyltransferase 1 (PRMT1) to interrupt proteasomal degradation of PRMT1, thereby promoting glycolysis‐related malignancy in hepatocellular carcinoma.^[^ ^8d^ ^]^ In addition to these metabolic enzymes, we here found the antioxidant enzyme PRDX6 also exhibits nonenzymatic function to promote ovarian cancer progression by binding and upregulating NNMT. It has been reported that PRDX6 prevents ROS‐induced apoptosis in cisplatin‐treated ovarian cancer.^[^ ^21^ ^]^ Interestingly, we observed that *PRDX6* KD has no obvious effect on apoptosis induction in untreated ovarian cancer cells, but prominently attenuates cell proliferation and metastasis.

Both the enzymatic and nonenzymatic functions contribute to this tumor‐promoting role of PRDX6 in ovarian cancer. Efforts have been made to identify PRDX6 inhibitors for cancer treatment. For example, the natural product thiacremonone was reported to bind PRDX6 to suppress its peroxidase activity, resulting in growth inhibition of lung cancer cells.^[^ ^22^ ^]^ Withangulatin A, another natural small molecule, was characterized as a covalent PRDX6 inhibitor to exhibit anticancer activity against non‐small cell lung cancer by binding and inhibiting PRDX6's peroxidase and PLA_2_ activities.^[^ ^15^ ^]^ However, the nonenzymatic function of PRDX6 may compromise the anticancer effects or confer resistance of these PRDX6 inhibitors specifically targeting its enzymatic activities. Apart from PRDX6, superoxide dismutase 1 (SOD1), another antioxidant enzyme catalyzing superoxide to hydrogen peroxide, has been reported to exert nonenzymatic activities. For instance, SOD1 suppresses WNT signaling and upregulates EGFR ligand expression to support intestinal stem cell growth, or acts as a nuclear transcription factor to promote the expression of oxidative stress responsive genes for resisting oxidative DNA damage, independently of its enzymatic activity.^[^ ^23^ ^]^ However, whether the nonenzymatic functions of SOD1 play roles in cancer remains to be further investigated. Unraveling more unorthodox functions of redox enzymes will pave new avenues for understanding the dark side mechanisms of cancer progression, and facilitate the development of innovative therapeutic interventions.

The nonenzymatic mechanism of PRDX6 in promoting ovarian cancer progression underscores the upregulation of NNMT. NNMT is an intracellular methyltransferase that catalyzes the *N*‐methylation of nicotinamide (NAM, the precursor of NAD^+^) to generate 1‐methylnicotinamide (1‐MNA) using SAM as the methyl donor.^[^ ^17^, ^24^ ^]^ Given its function in consuming NAD^+^ precursor and SAM, NNMT is emerging as a crucial regulator in cellular redox and energy metabolism, as well as epigenetic regulation.^[^ ^17^, ^25^ ^]^ Accumulating evidence has indicated the overexpression of NNMT with an oncogenic role in various cancer types. For example, NNMT‐mediated decrease in SAM level can attenuate PP2A methylation to inhibit PP2A activity, or reduce histone methylation to suppress E‐cadherin expression, resulting in increased growth or metastasis of cancer cells in breast cancer, esophageal squamous cell carcinoma or glioblastoma.^[^ ^7^, ^17^, ^26^ ^]^ NNMT‐catalyzed production of 1‐MNA was found to promote cell cycle progression in breast cancer by inhibiting UBC12/Cullin‐1‐mediated p27 degradation.^[^ ^27^ ^]^ However, how NNMT is upregulated in cancer remains unclear. Our study showed that PRDX6 interacts with NNMT to diminish its proteasomal degradation, leading to the upregulation of NNMT in ovarian cancer. Although the inhibition of NNMT proteasomal degradation has been reported in gastric cancer,^[^ ^28^ ^]^ we here further characterized TRIM56 as the key E3 ubiquitin ligase for NNMT ubiquitination, and identified lysine 23 and 210 as its major ubiquitination sites. Previous studies have indicated that NNMT promotes treatment resistance in ovarian cancer and is associated with poor prognosis of ovarian cancer patients,^[^ ^29^ ^]^ but the mechanism underlying NNMT's tumor‐promoting function in ovarian cancer is unclear. Our study found that PRDX6‐mediated NNMT upregulation activates MAPK pathway, thereby provoking the proliferation and metastasis of ovarian cancer cells. In addition to the function of NNMT expressed in cancer cells, NNMT in stromal cells also plays a crucial role in cancer. A recent study revealed that stromal NNMT supports ovarian cancer progression by driving the phenotype of cancer‐associated fibroblasts, during which the depletion of SAM and reduction of histone methylation associating with gene expression alterations were involved.^[^ ^30^ ^]^

In conclusion, our results demonstrated that beyond its canonical enzyme activities, PRDX6 promotes the malignant progression of ovarian cancer through a nonenzymatic mechanism, by which PRDX6 interacts with NNMT and suppresses TRIM56‐mediated ubiquitination degradation of NNMT. Our findings provide novel insights into the oncogenic mechanism of the PRDXs family and suggest the PRDX6‐NNMT axis as a potential treatment target for ovarian cancer.

## 4 Experimental Section

### Cell Culture

SKOV3, A2780, OVCAR5, and HEK293T cells were purchased from the Bank of Type Culture Collection of the Chinese Academy of Sciences. Normal ovarian epithelial cell lines IOSE80 and HOSEpiC were generously provided by Dr. Yinglan Zhao from the State Key Laboratory of Biotherapy at Sichuan University (Chengdu, China). Cells were cultured in Dulbecco's modified Eagle medium (DMEM) supplemented with 10% fetal bovine serum (VivaCell Biosciences, C04001) and 100 U mL^−1^ penicillin‐streptomycin (HyClone, SV30010). Cells were grown in a humidified chamber at 37 °C under a 5% CO_2_ atmosphere. Short tandem repeat (STR) analysis was performed for each cell line. All cells were verified to be mycoplasma‐free.

### Reagents

Antibodies against PRDX6 (ab92322), GFP (ab32146), and HA (ab18181) were purchased from Abcam. Antibodies against NNMT (15123‐1‐AP), Myc (16286‐1‐AP), and TRIM56 (25509‐1‐AP) were purchased from Proteintech. Antibody against GFP (11814460001) was purchased from Roche. Antibodies against PP2A (2038T), p‐MEK (9154T), and MEK (8727T) were purchased from Cell Signaling Technology. Antibodies against FLAG (F3165) and Ki‐67 (AB9260) were purchased from Sigma‐Aldrich. Antibodies against p38 (sc‐7972), p‐p38 (sc‐7973), ERK (sc‐514302), p‐ERK (sc‐81492), JNK (sc‐7345), p‐JNK (sc‐6254), and methyl‐PP2A (SC‐81603) were purchased from Santa Cruz Biotechnology. Antibody against β‐actin (AC026) was purchased from ABclonal.

CHX (HY‐12320), MG132 (HY13259), and withangulatin A (HY‐N10303) were purchased from MedChemExpress. Lipofectamine 3000 (L3000015) was purchased from Thermo Fisher Scientific. MTT (M2128) was purchased from Sigma–Aldrich.

### DNA Constructs and Mutagenesis

The cDNA encoding full‐length human PRDX6 was generously provided by Dr. Ji Cao from the Institute of Pharmacology and Toxicology at Zhejiang University (Hangzhou, China). The cDNA of full‐length human NNMT was cloned from SKOV3 cells. The cDNAs of PRDX6 and NNMT were then cloned into pcDNA3.1, pLenti‐6.3‐Flag, peGFP‐N1, or pCDH‐Flag vectors to generate overexpression plasmids with different tags. For point mutations of PRDX6 (MUT: D31A/S32A/C47S) and NNMT (K23R, K96R, and K210R), the plasmids were generated using a Fast Site‐directed Mutagenesis Kit (TransGen Biotech, FM111). For truncation mutations of PRDX6 (1‐144aa and 145–224aa) and NNMT (1‐58aa, 59–199aa, and 200–264aa), the cloned truncates amplified by polymerase chain reaction were inserted into pcDNA3.1‐mcherry‐Flag and peGFP‐N1 vectors, respectively.

The shRNA sequences for *NNMT* were designed using the BROAD online tool (https://portals.broadinstitute.org/gpp/public/), while the sgRNA sequences for *PRDX6* were designed using the MIT online tool (http://crispr.mit.edu). The annealed shRNA and sgRNA oligos were inserted into pLKO.1 and lentiCRISPRv2 vectors, respectively. The shRNA and sgRNA targeting sequences are listed as follows:

sh*NNMT*: 5′‐TGCAGAAAGCCAGATTCTTAA‐3′
sg*PRDX6* #1: 5′‐TCTTTGGTGAAGACTCCTTT‐3′
sg*PRDX6* #2: 5′‐ATCCTCTACCCAGCTACCAC‐3′

### Transfection

SKOV3, A2780, and HEK293T cells were transfected with different plasmids or siRNA using Lipofectamine 3000 following the manufacturer's protocol. The transfection efficiency was assessed 48 h post‐transfection using immunoblotting analysis. siRNA sequences were synthesized by GenePharma as follows:

si*PRDX6* #1: 5′‐GAAUGUUAAGAUGAUUGCCUUUCA‐3′
si*PRDX6* #2: 5′‐GGUGAAGACUCCUUUCGGGTT‐3′
si*TRIM56*: 5′‐UGUGGAUAAGAAGGGCUACAUTT‐3′

### Generation of Stable Cell Pools

To generate stable cell pools, the shRNA plasmids, sgRNA plasmids, or overexpression plasmids were transfected into HEK293T cells along with packing plasmids. The supernatants were collected and filtered through a 0.45 µm membrane (Millipore, SLGPR33RS) at 24 h after transfection. SKOV3 and A2780 cells were then infected with the collected lentivirus supernatants along with polybrene (2 µg mL^−1^) treatment for 24 h. Infected cells were then selected with puromycin (2 µg mL^−1^) for 1 week, followed by immunoblotting analysis for verification. To rescue PRDX6 (WT or MUT) in *PRDX6* KO cells, the pQCHIX vector and packing plasmids were co‐transfected into HEK293T cells to generate retrovirus expressing PRDX6‐WT or PRDX6‐MUT. After infection, the cells were selected with hygromycin (200 µg mL^−1^) for 1 week. The rescue efficiency was examined by immunoblotting analysis.

### Immunoblotting and Immunoprecipitation

Immunoblotting and immunoprecipitation were performed as previously described.^[^ ^31^ ^]^ For immunoblotting analysis, cells were washed twice with ice‐cold PBS and subsequently lysed in RIPA lysis buffer (50 mm Tris‐HCl, pH 7.4; 150 mM NaCl; 1 mM EDTA; 1% NP‐40; 1% Sodium deoxycholate). The protein concentrations were quantified using the Bradford reagent (Bio‐Rad Laboratories, 5000205). The protein lysates were subjected to SDS‐PAGE separation and then transferred to PVDF membranes (MilliporeSigma, ISEQ0001). Following a blocking step in TBST with 5% skimmed milk for 1.5 h, the membranes were incubated with the primary antibody overnight at 4 °C, followed by incubation with the secondary antibody for 1 h at room temperature. Finally, Immobilon Western HRP Substrate (MilliporeSigma, WBKLS0500) was employed to visualize the immunoreactive bands using a ChemiScope 6000 Touch (Clinx Science Instruments).

For immunoprecipitation, protein lysates were prepared with cold IP lysis buffer (20 mm Tris‐HCl, pH 7.4; 137 mM NaCl; 0.5% NP‐40; 0.5 mM EDTA). The lysates were incubated with Flag beads (MilliporeSigma, A2220) at 4 °C for 3 h, or with the indicated primary antibody at 4 °C overnight followed by incubation with protein A/G agarose beads (Millipore, IP10) for 3 h. Subsequently, the beads were washed four times with wash buffer (20 mm Tris‐HCl, pH 7.4; 274 mm NaCl; 0.5% NP‐40; 0.5 mM EDTA), and then boiled with loading buffer for immunoblotting analysis.

### Mass Spectrometry (MS) Analysis

For tandem mass tags (TMT)‐labeled quantitative proteomics analysis, 1 × 10^7^ cells were harvested and lysed. The protein solution was reduced with dithiothreitol, alkylated with iodoacetamide, and digested with sequencing‐grade trypsin (Promega, V5111). Peptides were then labeled with isobaric TMT tags according to the manufacturer's protocol (Thermo Fisher Scientific, 90061). Subsequently, the TMT‐labelled peptides were mixed and desalted using C18 ZipTips (Millipore). The desalted peptides were then analyzed using a Q Extractive Plus Orbitrap LC‐MS/MS System (Thermo Fisher Scientific). The differentially expressed proteins were identified by Proteome Discoverer 1.2 software (Thermo Fisher Scientific).

To identify the potential interacting proteins of PRDX6 or NNMT, co‐IP combined with MS analysis was performed as previously described.^[^ ^32^ ^]^ In brief, HEK293T cells overexpressing PRDX6‐GFP‐Flag or NNMT‐GFP were lysed with ice‐cold IP lysis buffer and incubated with Flag beads or GFP antibody. The immunoprecipitated proteins were separated by SDS‐PAGE and stained with Coomassie Bright Blue. The protein bands were excised and digested in gel with sequencing‐grade trypsin and then subjected to a Q Exactive Plus Orbitrap LC‐MS/MS System (Thermo Fisher Scientific) for label‐free quantitative analysis. The MS/MS data were processed using Proteome Discoverer 1.2 software (Thermo Fisher Scientific).

Identification of the ubiquitination sites of NNMT was performed as described in a previous study.^[^ ^33^ ^]^ Briefly, HEK293T cells were co‐transfected with NNMT‐Flag and HA‐Ub plasmids, followed by treatment with MG132 for 6 h. Cells were then lysed using ice‐cold IP lysis buffer and subjected to IP analysis using Flag beads. Subsequently, the purified NNMT‐Flag proteins were enriched by competitive elution using Flag peptides (Sigma‐Aldrich, F4799). The elution pools were incubated with HA antibody at 4 °C overnight, followed by enrichment with protein A/G magnetic beads (MCE, HY‐K0202) for 3 h. The ubiquitinated NNMT proteins were eluted and subjected to an HPLC system and Q Exactive Plus LC‐MS/MS System (Thermo Fisher Scientific). The acquired MS/MS data were processed using Proteome Discoverer 1.3 (Thermo Fisher Scientific).

### RNA‐Seq Analysis

Total RNA was extracted from ovarian cancer cells using TRIzol (Thermo Fisher Scientific, 15596026). Subsequently, RNA sequencing was performed using the Illumina NovaSeq 6000 platform by Novogene Inc. (Tianjin, China). Differentially expressed genes were filtered using the DEGseq package for significance analysis and false discovery rate analysis. The ClusterProfiler package was employed for enrichment analysis.

### Immunohistochemical (IHC) Staining

Tissue microarray (purchased from Shanghai Outdo Biotech, Shanghai, China. Ethical approval number: SHYJS‐CP‐1804029) consisting of 147 ovarian tumor tissues with informed written consent from patients were subjected to IHC staining to evaluate the protein levels of PRDX6 and NNMT. The clinical characteristics of ovarian patients are shown in Table S6 (Supporting Information). In brief, the sections were deparaffinized, rehydrated, and blocked by 3% H_2_O_2_. Antigen retrieval was then performed by heating the slides in citrate buffer. After blocking with 10% goat serum at room temperature for 1.5 h, the sections were incubated with the primary antibody at 4 °C overnight, followed by treatment with MaxVision HRP‐Polymer anti‐Mouse/Rabbit IHC Kit (MXB Biotechnology, 5010) and DAB Kit (MXB Biotechnology, 0031). Subsequently, the slides were counterstained with Mayer's hematoxylin (MXB Biotechnology, CST1096) to visualize the nuclei. The stained sections were visualized using Slide Scanner Systems (3DHISTECH, Pannoramic MIDI). Finally, the IHC staining intensity was independently evaluated by two investigators and scored as follows: negative (0), weak (1), moderate (2), and strong (3). The Score was calculated as (3 × % of strong staining) + (2× % of moderate staining) + (1 × % of weak staining).

### In Vitro Cell Growth and Proliferation Assays

For MTT assays, cells were cultured in 96‐well plates (2000 cells/well). At indicated time points (0, 1, 2, 3, and 4 days), 20 µL MTT solution (5 mg mL^−1^) was added to each well and incubated for 4 h. Subsequently, the supernatants were removed, and the formazan crystals were solubilized using DMSO. The absorbance at 570 nm for each well was measured using a Varioskan LUX Multimode Microplate Reader (Thermo Fisher Scientific).

For colony formation assay, cells were seeded into 24‐well plates (1000 cells/well) and cultured for 15 days. Following three washes with ice‐cold PBS, the colonies were fixed with methyl alcohol and stained with crystal violet for 4 h. The number of colonies was then counted for statistical analysis.

For 5‐ethynyl‐20‐deoxyuridine (EdU) assays, cells were cultured into 96‐well plates (5000 cells/well) for 24 h. Each well was added with 50 µM EdU and incubated for 4 h according to the protocol of the EdU incorporation Kit (RiboBio, C10310). Subsequently, cells were fixed with 4% paraformaldehyde, permeabilized with 0.1% Triton X‐100, and stained with a reaction cocktail. The nuclei were stained with Hoechst 33342. Finally, the fluorescent images were captured using a fluorescence microscope (ZEISS, Axio Observer 7).

### In Vitro Metastasis Assays

For the wound healing assay, cells were seeded in 6‐well plates. When reaching ≈100% confluence, the linear and parallel wound was scraped with a pipette tip. At indicated time points (0, 24, and 48 h), the gap of the wound was imaged and measured at the same position using a light microscope.

For transwell assay, 5 × 10^4^ cells suspended in 200 µL serum‐free medium were seeded in the upper chamber of a Transwell plate (Corning, 3422). The lower part of the transwell unit was added with 800 µL serum‐containing medium. After incubation for 24 h, the cells in the upper chamber were fixed with 4% paraformaldehyde and stained with crystal violet for 4 h. Unmigrated cells were removed by softly swiping the upper chamber with cotton swabs. The migrated cells were then imaged and counted using a light microscope. For the invasion assay, Matrigel (Corning, 354277) was added to the chamber surface to assess the invasion ability of cells following the manufacturer's instruction.

### TUNEL Assay

Cells were plated onto glass cover slips in 24‐well plates, and then fixed with 4% paraformaldehyde at room temperature for 30 min. Subsequently, cells were permeabilized using 0.1% Triton X‐100 for 30 min. The DeadEnd Fluorometric TUNEL Kit (Promega, G3250) was then used to label the TUNEL‐positive cells. The nuclei were stained with DAPI. Specifically, DNase‐treated cells were employed as a positive control. Fluorescent images were captured using a ZEISS LSM 710 confocal microscope.

### Protein Half‐Life Assay

To examine the half‐life of the NNMT protein, cells were treated with CHX (40 µg mL^−1^) for the indicated time periods (0, 6, 12, and 24 h). The protein level of NNMT was then measured by immunoblotting analysis.

### PRDX6 Activity Assay

The peroxidase activity of PRDX6 was measured using a glutathione peroxidase (GPx) assay Kit (Cayman Chemical, 703102) following the manufacturer's instructions. The PLA_2_ activity of PRDX6 was measured using a PLA_2_ assay Kit (Cayman Chemical, 765021) according to the manufacturer's instructions.

### Animal Studies

For the growth of tumor xenografts, 5 × 10^5^ cells were subcutaneously injected into the flanks of five‐week‐old female NSG mice (HFK Bioscience). Tumor volumes were measured every three days when they reached ≈80 mm^3^, and calculated using the formula (length × width^2^)/2. After euthanasia, the tumor xenografts were collected and fixed with 4% paraformaldehyde for IHC analysis. For the peritoneal metastasis model, 5 × 10^5^ cells were inoculated into mice by intraperitoneal injection. Mice were euthanized at the end of the experiment, and the number of metastatic nodules was counted. All animal studies were performed in accordance with guidelines provided by the Institutional Animal Care and Treatment Committee of Sichuan University.

### Statistical Analysis

Statistical analysis was performed using GraphPad Prism 8.0 software. The two‐tailed student's *t*‐test was employed to analyze the variables between the two groups. Pearson correlation and linear regression analysis were conducted to determine the correlations between two factors. Two‐way ANOVA was used to compare the differences in cell and tumor growth rates. Survival analysis, including Kaplan‐Meier survival curve plotting and the log‐rank test, was conducted using SPSS software. All experiments were independently repeated at least three times. The data are presented as mean ± SD, and the significance was denoted as follows: ^*^ *P* < 0.05, ^**^ *P* < 0.01, ^***^ *P* < 0.001, n.s., not significant.

## Conflict of Interest

The authors declare no conflict of interest.

## Author Contributions

X.W., L.L., and M.W. contributed equally to this work. X.W., L.L., M.W., L.D., J.F., Y.Z., and S.L. conducted the experiments and analyzed the data. K.W. conceived and supervised the study. X.W., L.L., and K.W. wrote the manuscript.

## Supporting information



Supporting Information

## Data Availability

The data that support the findings of this study are available in the supplementary material of this article.
