# Supplementary material for: PRDX6 Prevents NNMT Ubiquitination and Degradation as a Nonenzymatic Mechanism to Promote Ovarian Cancer Progression
Source: Adv Sci (Weinh). 2025 Jan 30;12(12):2416484. doi: 10.1002/advs.202416484 (PMC11948025; doi:10.1002/advs.202416484)
Supplement: Supplementary file 1 — Supporting Information [file ADVS-12-2416484-s001.pdf]

ADVANCED SCIENCE
Open Access

Supporting Information

for *Adv. Sci.*, DOI 10.1002/advs.202416484

PRDX6 Prevents NNMT Ubiquitination and Degradation as a Nonenzymatic Mechanism to Promote Ovarian Cancer Progression

*Xingyun Wu, Li Luo, Mao Wang, Lixia Dong, Jiawu Fan, Yan Zeng, Sijia Li and Kui Wang**

Supporting Information

PRDX6 Prevents NNMT Ubiquitination and Degradation as a Nonenzymatic Mechanism to Promote Ovarian Cancer Progression

Xingyun Wu, Li Luo, Mao Wang, Lixia Dong, Jiawu Fan, Yan Zeng, Sijia Li, Kui Wang

Contents

Figure S1-S7

Table S1-S6

Figure S1. PRDX6 is upregulated in ovarian cancer and is associated with poor prognosis of ovarian cancer patients.

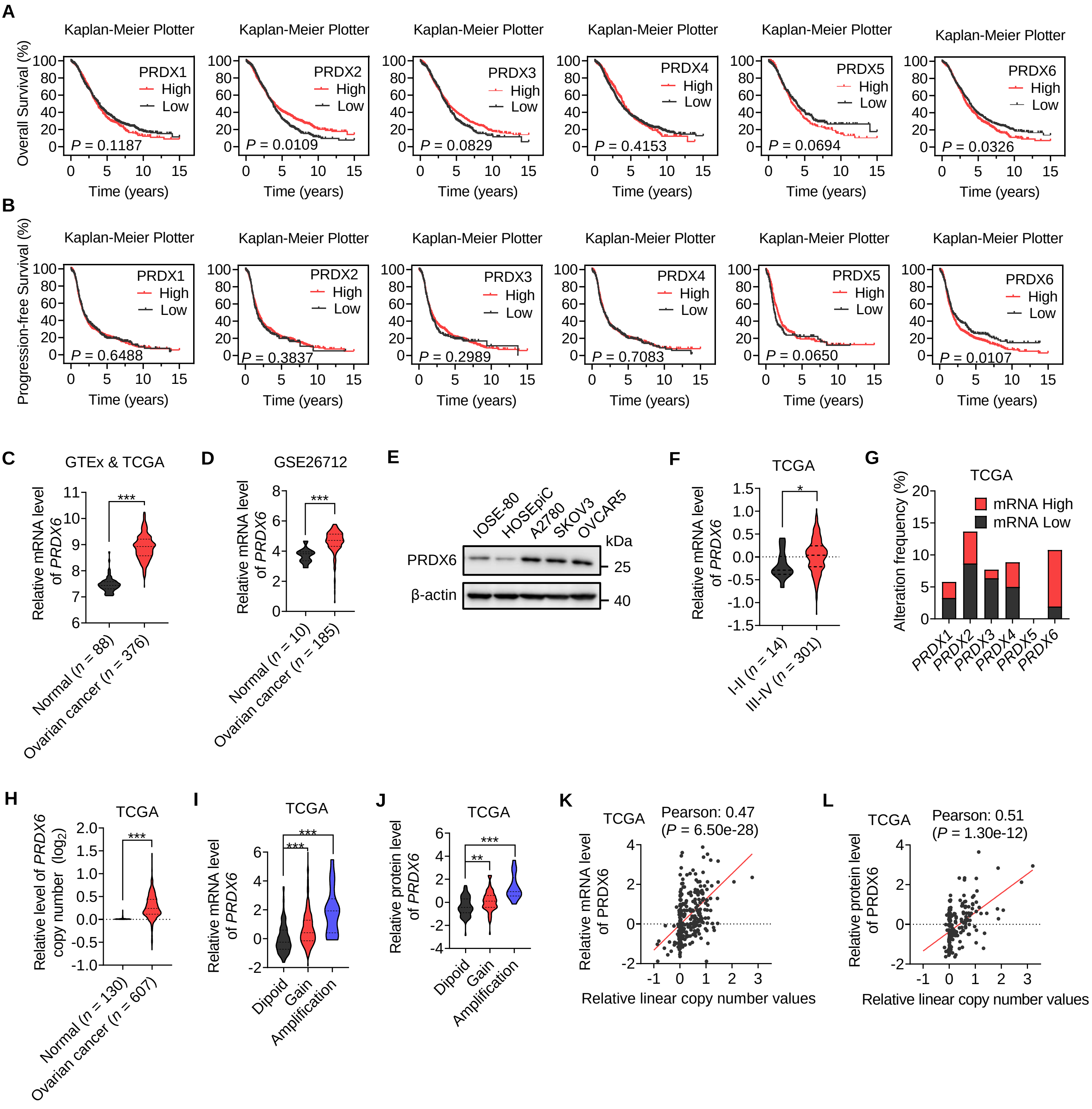

Figure S1. PRDX6 is upregulated in ovarian cancer and is associated with poor prognosis of ovarian cancer patients. (A and B) Overall survival (A, n = 1656) and progression-free survival (B, n = 1435) analysis based on the expression of different PRDXs in human ovarian cancer tissues using the Kaplan-Meier Plotter database. (C and D) Relative mRNA level of PRDX6 in ovarian cancer tissues compared with normal ovarian tissues by integrated analysis of the TCGA and GTEx datasets (C), and GSE26712 dataset (D). (E) Immunoblotting analysis of PRDX6 in ovarian cancer cells and normal ovarian epithelial cells. (F) Relative mRNA level of PRDX6 in ovarian cancer tissues with different stages in the TCGA dataset. (G) mRNA expression analysis of different PRDXs in ovarian cancer patients according to the cBioportal TCGA database. (H) Relative level of PRDX6 copy number in ovarian cancer patients using the Oncomine online tool. (I and J) Relative mRNA (I) and protein (J) levels of PRDX6 in ovarian cancer tissues with different PRDX6 copy number status using the TCGA database. (K and L) Pearson correlation test analyzing the relationship between PRDX6 mRNA (K) or protein (L) level and relative linear copy number values of PRDX6 in the TCGA database. Data are presented as mean ± SD. *$P < 0.05$, **$P < 0.01$, ***$P < 0.001$.

Figure S2. PRDX6 has no obvious effect on apoptosis in ovarian cancer cells.

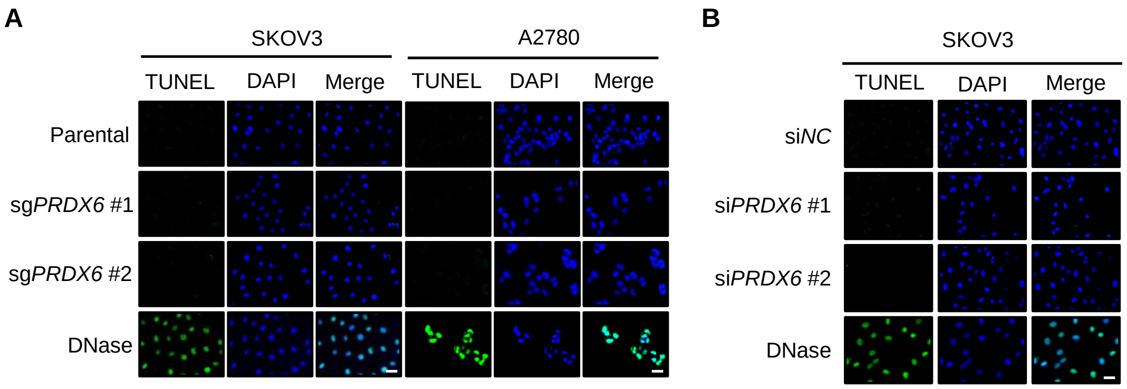

Figure S2. PRDX6 has no obvious effect on apoptosis in ovarian cancer cells. (A and B) TUNEL assay examining apoptosis of SKOV3 and A2780 cells with *PRDX6* knockout (A) or knockdown using siRNA (B). Scale bar, 100 μm.

Figure S3. PRDX6 inhibitor decreases the growth and metastasis of ovarian cancer cells.

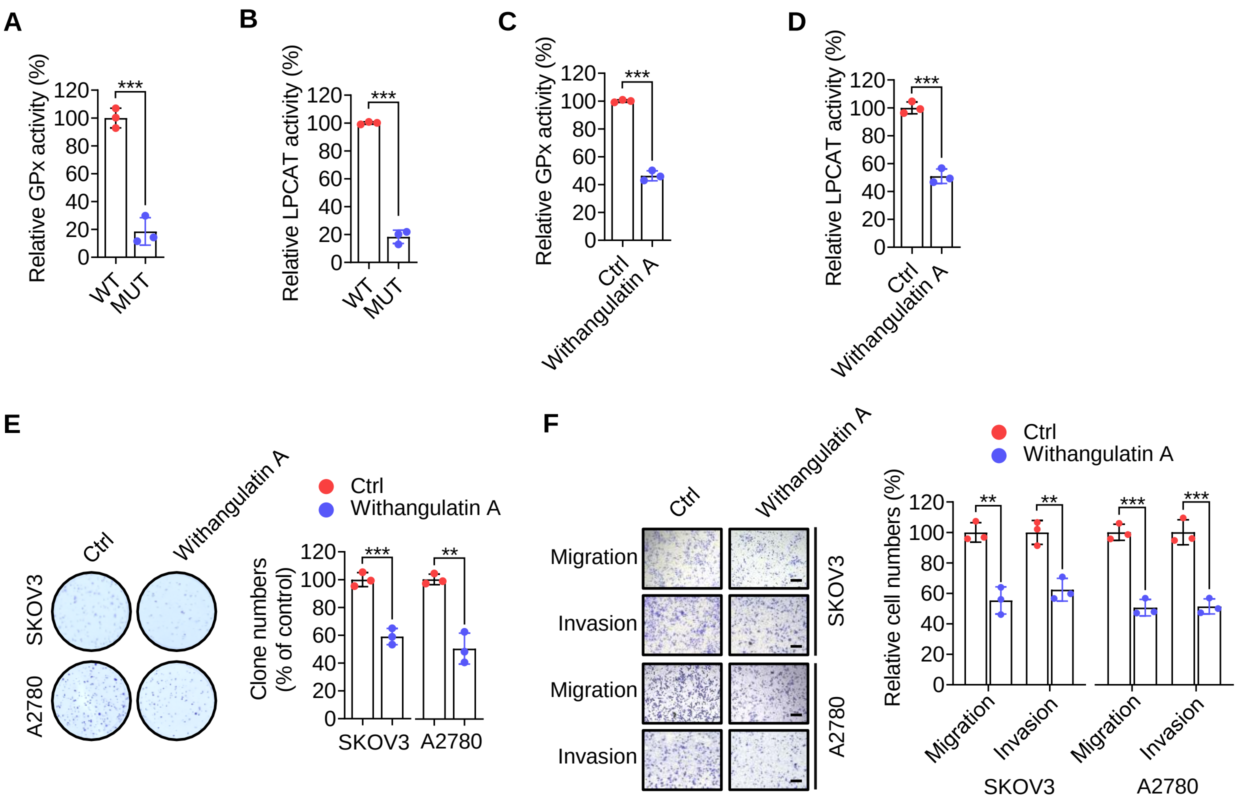

Figure S3. PRDX6 inhibitor decreases the growth and metastasis of ovarian cancer cells. (A and B) The glutathione peroxidase (GPx) activity (A) and PLA_2 activity (B) in cells expressing PRDX6-WT or PRDX6-MUT. (C and D) The GPx activity (C) and PLA_2 activity (D) in cells treated with or without withangulatin A for 24 h (5 μM). (E and F) Colony formation (E) and transwell (F) assays of SKOV3 and A2780 cells treated with or without withangulatin A for 24 h (5 μM). Scale bar, 100 μm. Results are representative of at least three independent experiments. Data are presented as mean ± SD. **P* < 0.05, ***P* < 0.01, ****P* < 0.001.

Figure S4. PRDX6 interacts with NNMT.

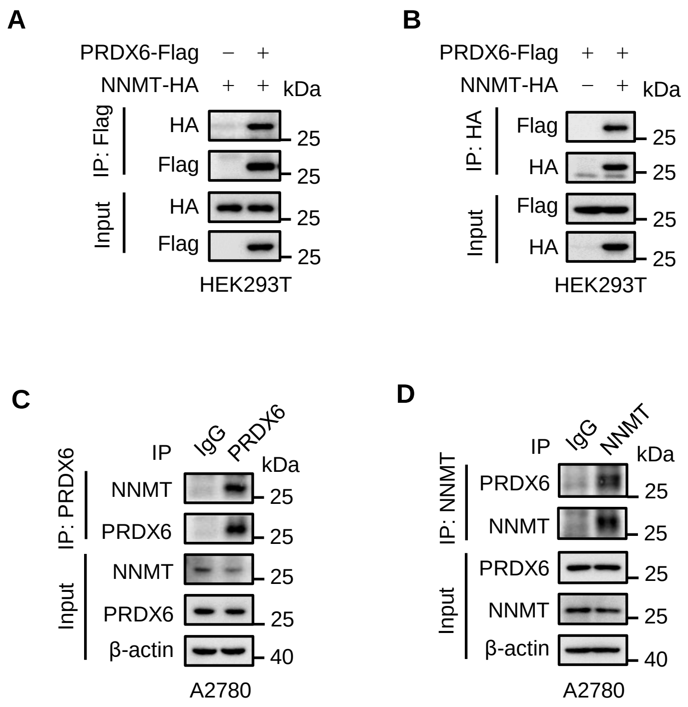

Figure S4. PRDX6 interacts with NNMT. (A and B) Reciprocal co-IP analysis of exogenous PRDX6 and NNMT in HEK293T cells co-expressing PRDX6-Flag and NNMT-HA. (C and D) Reciprocal co-IP analysis of endogenous PRDX6 and NNMT in A2780 cells. Results are representative of at least three independent experiments.

Figure S5. PRDX6 prevents the ubiquitin-mediated degradation of NNMT.

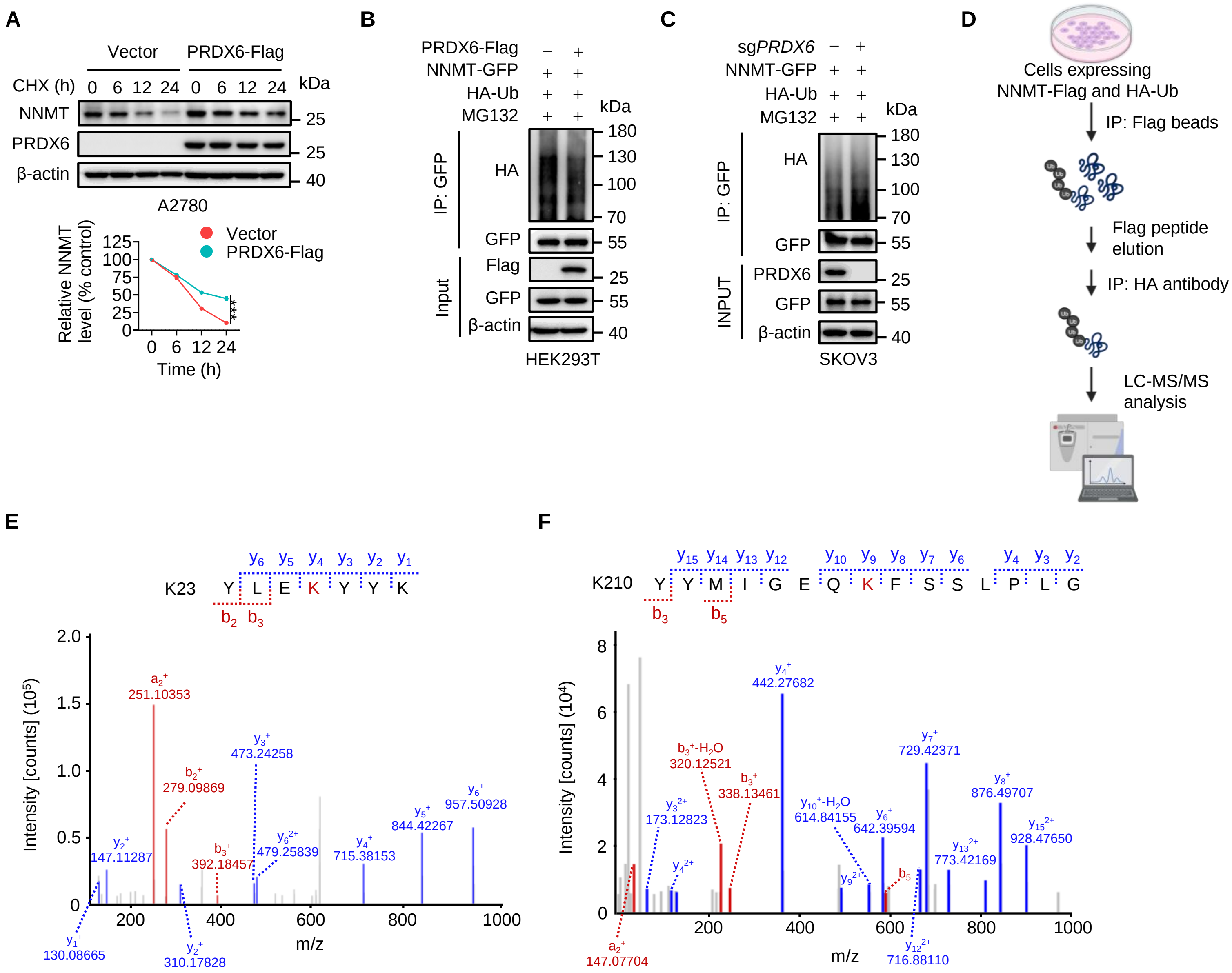

Figure S5. PRDX6 prevents the ubiquitin-mediated degradation of NNMT. (A) Immunoblotting analysis of NNMT in PRDX6-overxpressing SKOV3 and A2780 cells treated with or without cycloheximide (CHX) for the indicated time. Quantitation of NNMT protein level based on band intensity was shown (bottom). (B) NNMT-GFP and HA-Ub were co-expressed with or without PRDX6-Flag in HEK293T cells. After MG132 treatment, IP was performed using GFP antibody, followed by immunoblotting analysis with indicated antibodies. (C) NNMT-GFP and HA-Ub were co-expressed in SKOV3 cells with or without *PRDX6* KO. After MG132 treatment, IP was performed using GFP antibody, followed by immunoblotting analysis with indicated antibodies. (D) Schematic of the workflow for identifying the ubiquitination sites of NNMT by mass spectrometry analysis. (E and F) The MS spectra showing the ubiquitination sites of NNMT at lysine 23 (E) and 210 (F). Results are representative of at least three independent experiments. Data are presented as mean ± SD. *P < 0.05, **P < 0.01, ***P < 0.001.

Figure S6. TRIM56 is an E3 ubiquitin ligase required for the ubiquitin-mediated degradation of NNMT.

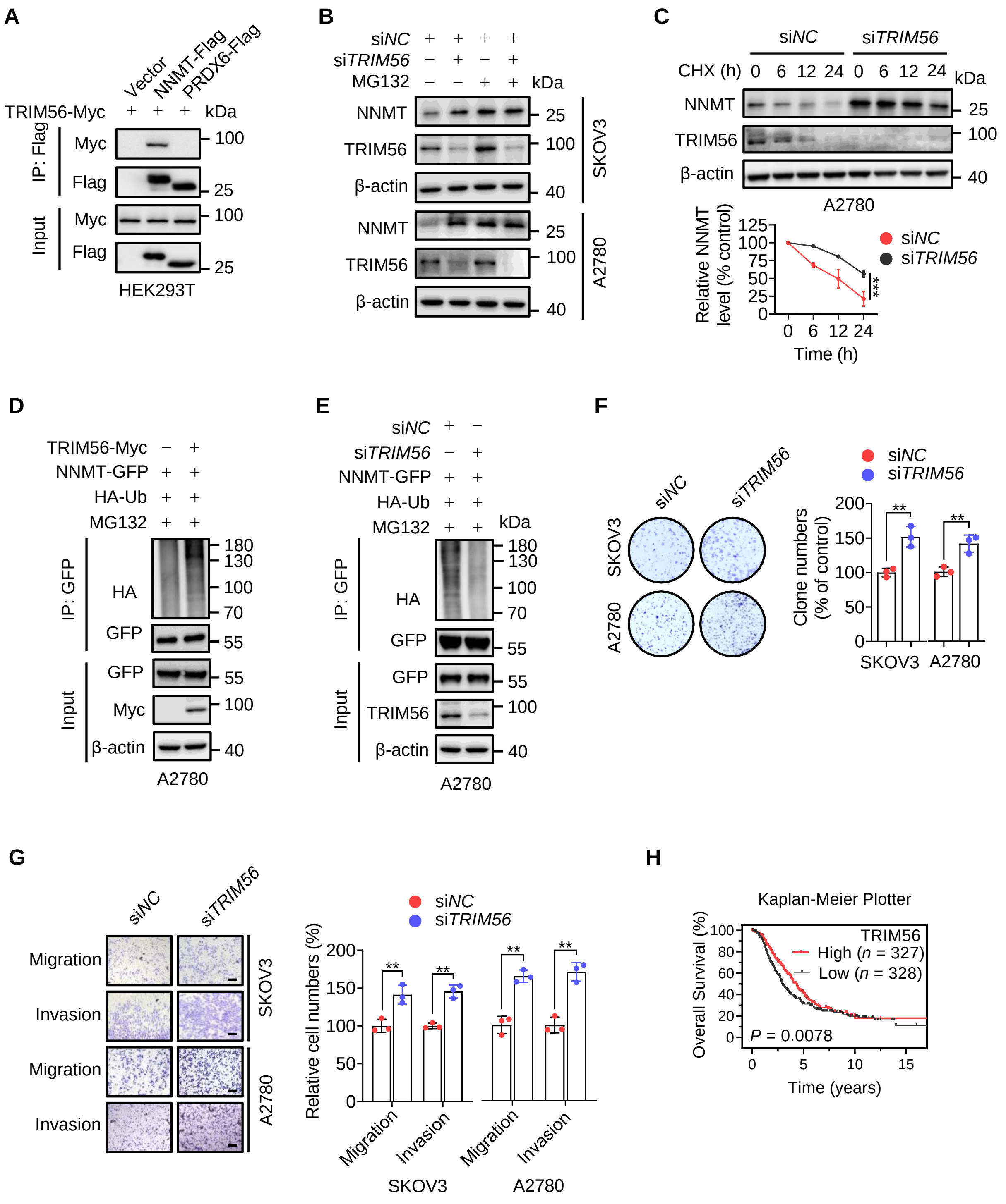

Figure S6. TRIM56 is an E3 ubiquitin ligase required for the ubiquitin-mediated degradation of NNMT. (A) Co-IP analysis of TRIM56-Myc with NNMT-Flag or PRDX6-Flag in HEK293T cells. (B) Immunoblotting analysis of NNMT in *TRIM56* knockdown (KD) SKOV3 and A2780 cells treated with or without MG132. (C) Immunoblotting analysis of NNMT in *TRIM56* KD SKOV3 and A2780 cells treated with or without CHX for the indicated time. Quantitation of NNMT protein level based on band intensity was shown (bottom). (D and E) A2780 cells with TRIM56 overexpression (D) or *TRIM56* KD (E) were transfected with NNMT-GFP and HA-Ub plasmids. After MG132 treatment, IP was performed using GFP antibody, followed by immunoblotting analysis with indicated antibodies. (F and G) Colony formation (F) and transwell (G) assays of SKOV3 and A2780 cells with or without *TRIM56* KD. Scale bar, 100 μm. (H) Overall survival analysis based on TRIM56 expression in human ovarian cancer tissues using the Kaplan-Meier Plotter database. Results are representative of at least three independent experiments. Data are presented as mean ± SD. *$P < 0.05$, **$P < 0.01$, ***$P < 0.001$.

Figure S7. NNMT upregulation contributes to the nonenzymatic function of PRDX6 in promoting the growth and metastasis of ovarian cancer cells

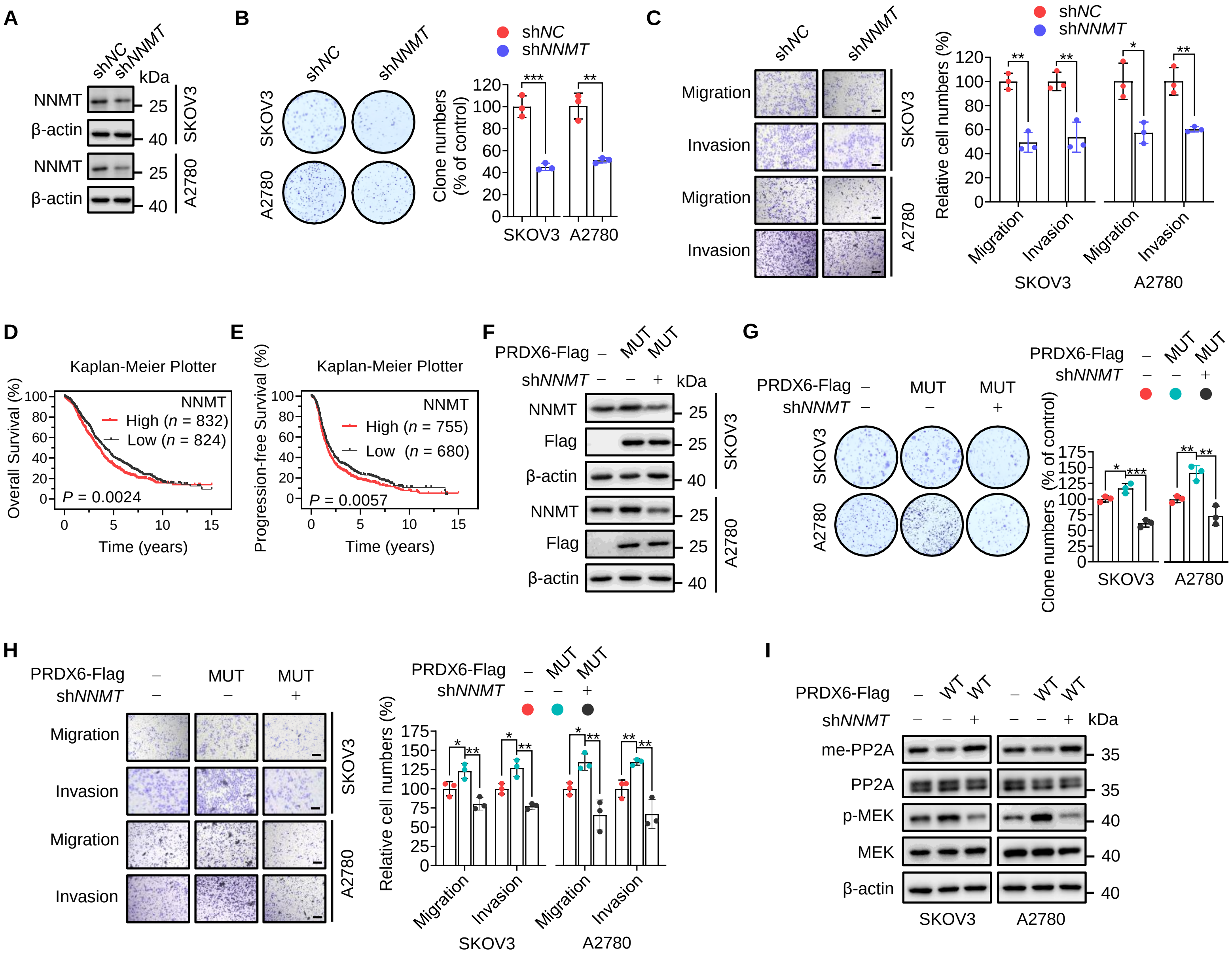

Figure S7. NNMT upregulation contributes to the nonenzymatic function of PRDX6 in promoting the growth and metastasis of ovarian cancer cells. (A) Immunoblotting analysis of NNMT in SKOV3 and A2780 cells with or without *NNMT* knockdown (KD). (B and C) Colony formation (B) and transwell (C) assays of SKOV3 and A2780 cells with or without *NNMT* KD. Scale bar, 100 μm. (D and E) Overall (D) and progression-free (E) survival analysis based on NNMT expression in human ovarian cancer tissues using the Kaplan-Meier Plotter database. (F) Immunoblotting analysis of NNMT in PRDX6-MUT-overexpressing SKOV3 and A2780 cells with or without *NNMT* KD. (G and H) Colony formation (G) and transwell (H) assays of PRDX6-MUT-overexpressing SKOV3 and A2780 cells with or without *NNMT* KD. Scale bar, 100 μm. (I) Immunoblotting analysis of PP2A, methyl-PP2A (me-PP2A), MEK, and phosphorylated MEK (p-MEK) in PRDX6-WT-overexpressing SKOV3 and A2780 cells with or without *NNMT* KD. Results are representative of at least three independent experiments. Data are presented as mean ± SD. *$P < 0.05$, **$P < 0.01$, ***$P < 0.001$.

Table S1. Potential PRDX6-interacting proteins identified by co-IP coupled with MS analysis.

Accession	Protein names	MW [kDa]	Protein score	Sequence coverage (%)	Unique Peptides
P30041	PRDX6	25	2321	63	13
P05141	SLC25A5	32.8	599	43	8
P14678	SNRPB	24.6	529	22	7
Q9NXV2	KCTD5	26.1	438	43	8
P60709	ACTB	41.7	419	18	6
P01614	IGKV2D-40	13.3	402	11	1
P62701	RPS4X	29.6	348	31	8
P18124	RPL7	29.2	346	38	9
P68363	TUBA1B	50.1	321	10	3
P06733	ENO1	47.1	270	7	2
P12236	SLC25A6	32.8	268	24	2
P11142	HSPA8	70.9	266	13	3
Q9BQA1	WDR77	36.7	245	25	6
A2NJV5	IGKV2-29	13.1	226	17	2
P04406	GAPDH	36	217	11	2
P07195	LDHB	36.6	209	4	1
P62241	RPS8	24.2	194	25	4
P28066	PSMA5	26.4	171	18	3
P08579	SNRPB2	25.5	170	17	3
P62906	RPL10A	24.8	169	23	4
P10809	HSPD1	61	144	3	1
P40261	NNMT	29.6	143	14	4
P39023	RPL3	46.1	135	6	3
P00918	CA2	29.2	133	23	4
P00338	LDHA	36.7	130	5	1
P26373	RPL13	24.2	121	12	3
P60900	PSMA6	27.4	120	10	2
Q9NPD3	EXOSC4	26.4	118	12	3
P50914	RPL14	23.4	113	13	3
Q9Y657	SPIN1	29.6	111	9	2
Q13885	TUBB2A	49.9	104	9	3
P68104	EEF1A1	50.1	102	6	3
A5A3E0	POTEF	121.4	100	1	1
P07900	HSP90AA1	84.6	93	2	1
P54652	HSPA2	70	82	6	1
P52732	KIF11	119.1	78	3	3
P0DMV8	HSPA1A	70	77	6	1
Q9NY12	GAR1	22.3	77	7	2
P81605	DCD	11.3	75	23	2
O14744	PRMT5	72.6	73	3	2
P02768	ALB	69.3	71	4	2
P25789	PSMA4	29.5	65	4	1
A0A075B6R	IGKV2D-24	13.1	64	8	1
Q12931	TRAP1	80.1	58	2	1
Q70IA6	MOB2	26.9	57	5	1
P47914	RPL29	17.7	56	9	1
P00558	PGK1	44.6	51	3	1
Q8TAA3	PSMA8	28.5	47	4	1
P36957	DLST	48.7	47	2	1
Q5JUX0	SPIN3	29.2	45	4	1
Q15393	SF3B3	135.5	45	1	1
P38646	HSPA9	73.6	45	1	1
Q9BUZ4	TRAF4	53.5	43	1	1
P60842	EIF4A1	46.1	42	2	1
P22061	PCMT1	24.6	41	6	1

Table S1. Potential PRDX6-interacting proteins identified by co-IP coupled with MS analysis.

Accession	Protein names	MW [kDa]	Protein score	Sequence coverage (%)	Unique Peptides
P30041	PRDX6	25	2321	63	13
P05141	SLC25A5	32.8	599	43	8
P14678	SNRPB	24.6	529	22	7
Q9NXV2	KCTD5	26.1	438	43	8
P60709	ACTB	41.7	419	18	6
P01614	IGKV2D-40	13.3	402	11	1
P62701	RPS4X	29.6	348	31	8
P18124	RPL7	29.2	346	38	9
P68363	TUBA1B	50.1	321	10	3
P06733	ENO1	47.1	270	7	2
P12236	SLC25A6	32.8	268	24	2
P11142	HSPA8	70.9	266	13	3
Q9BQA1	WDR77	36.7	245	25	6
A2NJV5	IGKV2-29	13.1	226	17	2
P04406	GAPDH	36	217	11	2
P07195	LDHB	36.6	209	4	1
P62241	RPS8	24.2	194	25	4
P28066	PSMA5	26.4	171	18	3
P08579	SNRPB2	25.5	170	17	3
P62906	RPL10A	24.8	169	23	4
P10809	HSPD1	61	144	3	1
P40261	NNMT	29.6	143	14	4
P39023	RPL3	46.1	135	6	3
P00918	CA2	29.2	133	23	4
P00338	LDHA	36.7	130	5	1
P26373	RPL13	24.2	121	12	3
P60900	PSMA6	27.4	120	10	2
Q9NPD3	EXOSC4	26.4	118	12	3
P50914	RPL14	23.4	113	13	3
Q9Y657	SPIN1	29.6	111	9	2
Q13885	TUBB2A	49.9	104	9	3
P68104	EEF1A1	50.1	102	6	3
A5A3E0	POTEF	121.4	100	1	1
P07900	HSP90AA1	84.6	93	2	1
P54652	HSPA2	70	82	6	1
P52732	KIF11	119.1	78	3	3
P0DMV8	HSPA1A	70	77	6	1
Q9NY12	GAR1	22.3	77	7	2
P81605	DCD	11.3	75	23	2
O14744	PRMT5	72.6	73	3	2
P02768	ALB	69.3	71	4	2
P25789	PSMA4	29.5	65	4	1
A0A075B6R	IGKV2D-24	13.1	64	8	1
Q12931	TRAP1	80.1	58	2	1
Q70IA6	MOB2	26.9	57	5	1
P47914	RPL29	17.7	56	9	1
P00558	PGK1	44.6	51	3	1
Q8TAA3	PSMA8	28.5	47	4	1
P36957	DLST	48.7	47	2	1
Q5JUX0	SPIN3	29.2	45	4	1
Q15393	SF3B3	135.5	45	1	1
P38646	HSPA9	73.6	45	1	1
Q9BUZ4	TRAF4	53.5	43	1	1
P60842	EIF4A1	46.1	42	2	1
P22061	PCMT1	24.6	41	6	1

P23528	CFL1	18.5	39	10	1
Q86VD1	MORC1	112.8	38	1	1
P23588	EIF4B	69.1	36	2	1
P52597	HNRNPF	45.6	35	3	1
P62249	RPS16	16.4	32	7	1
A6NMY6	ANXA2P2	38.6	32	5	1
P31153	MAT2A	43.6	25	4	1
P04075	ALDOA	39.4	23	3	1
Q96HU8	DIRAS2	22.5	22	6	1

Table S2. Differentially expressed proteins in *PRDX6* KO SKOV3 cells identified by TMT proteomics analysis.

Protein accession	Protein name	Ratio	P value	Regulated Type	MW [kDa]	Coverage [%]	Unique peptides
A3KN83	SBNO1	0.733	0.007708251	Down	154.31	10.4	12
P21980	TGM2	0.579	3.81891E-06	Down	77.328	29.8	17
P82675	MRPS5	1.529	2.47366E-08	Up	48.006	24	10
P09001	MRPL3	1.431	0.001059235	Up	38.632	19	7
Q6NUK1	SLC25A24	1.405	2.18883E-05	Up	53.354	28.7	14
Q8N4H5	TOMM5	0.769	0.02777461	Down	6.0352	45.1	3
P51398	DAP3	1.306	0.001960281	Up	45.566	17.6	6
P51570	GALK1	0.695	0.001137594	Down	42.272	36.5	14
P49773	HINT1	1.383	0.010537876	Up	13.802	30.2	3
P12268	IMPDH2	0.597	0.014123005	Down	55.804	44.2	18
P09758	TACSTD2	1.694	0.025319271	Up	35.709	19.8	5
O15305	PMM2	1.362	0.024198897	Up	28.082	43.9	10
O43660	PLRG1	0.761	3.6297E-06	Down	57.193	33.3	14
Q9UHR5	SAP30BP	0.614	0.01440109	Down	33.87	21.8	7
P31040	SDHA	0.733	0.011216555	Down	72.691	34.6	19
Q14249	ENDOG	0.746	0.036092859	Down	32.62	20.2	5
P49591	SARS1	1.375	5.4913E-17	Up	58.777	40.3	19
P30536	TSPO	1.46	0.00770284	Up	18.828	13.6	3
P12955	PEPD	0.652	0.000109013	Down	54.548	31	14
P11216	PYGB	1.432	3.31373E-08	Up	96.695	40.3	29
O15533	TAPBP	2.046	2.29704E-05	Up	47.625	12.1	5
P22695	UQCRC2	1.928	0.016957332	Up	48.442	24.7	9
Q9UBG0	MRC2	0.633	0.013261875	Down	166.67	11.6	14
Q00653	NFKB2	1.613	3.35487E-07	Up	96.748	24.7	20
Q6IN85	PPP4R3A	0.747	0.020397414	Down	95.367	19.3	8
P27105	STOM	0.407	0.000524176	Down	31.73	33.7	8
Q8WUY1	THEM6	1.325	0.002385461	Up	23.865	26	6
P31930	UQCRC1	1.479	0.001723664	Up	52.645	31.7	11
Q9NR46	SH3GLB2	1.523	0.004777161	Up	43.973	28.4	13
Q9UBT2	UBA2	0.659	1.43507E-05	Down	71.223	26.6	15
P82933	MRPS9	1.323	0.020850273	Up	45.834	29.3	13
P78330	PSPH	1.443	0.000875645	Up	25.007	35.1	6
P30044	PRDX5	1.551	1.96355E-13	Up	22.086	36.4	6
Q9H0U4	RAB1B	1.377	0.049999774	Up	22.171	53.7	5
O94885	SASH1	0.397	0.006071005	Down	136.65	1.5	2
Q96BP3	PPWD1	0.731	0.044545588	Down	73.574	25.9	15
Q99460	PSMD1	0.756	3.433E-15	Down	105.84	36.7	29
O15294	OGT	0.684	0.000679773	Down	116.92	12.4	13
P29034	S100A2	0.639	5.11549E-05	Down	11.117	26.5	3
O43175	PHGDH	1.38	7.81417E-06	Up	56.65	43.2	19
P04179	SOD2	1.756	7.25431E-08	Up	24.75	41.4	8
P40261	NNMT	0.767	0.001133404	Down	29.574	40.5	10
P28331	NDUFS1	1.4	0.001150262	Up	79.467	27.9	17
P07737	PFN1	0.766	8.41876E-05	Down	15.054	70	5
Q9UHD8	SEPTIN9	0.769	1.08304E-13	Down	65.401	37	22
Q3YEC7	RABL6	1.301	2.22262E-05	Up	79.548	15	10
P43007	SLC1A4	1.654	0.019297554	Up	55.722	13.7	5
Q8TEM1	NUP210	1.425	1.18192E-06	Up	205.11	18	30
Q14914	PTGR1	0.624	0.001490196	Down	35.869	31.9	9
Q14684	RRP1B	0.739	6.52589E-06	Down	84.427	23.6	16
Q70UQ0	IKBIP	0.75	0.000328801	Down	39.309	36.6	14
Q9HCE1	MOV10	1.437	3.54896E-05	Up	113.67	31.5	26
Q14764	MVP	1.337	0.005930792	Up	99.326	41.9	29
P04183	TK1	0.505	4.45148E-07	Down	25.468	50.4	10

P42704	LRPPRC	1.483	5.86107E-12	Up	157.9	48.8	62
P36776	LONP1	1.455	8.75223E-05	Up	106.49	25.5	22
O00483	NDUFA4	1.765	8.06883E-05	Up	9.3697	55.6	5
P15531	NME1	0.746	0.003552732	Down	17.149	57.2	4
P42226	STAT6	1.864	6.64051E-05	Up	94.134	13	10
Q8WX92	NELFB	0.748	0.021084155	Down	65.697	15.9	9
O00469	PLOD2	1.501	0.0004692	Up	84.685	19.4	12
P55786	NPEPPS	0.657	4.80629E-11	Down	103.28	32.3	29
Q06323	PSME1	1.437	4.1088E-07	Up	28.723	49	12
P49756	RBM25	0.75	0.011613194	Down	100.18	11.7	11
Q7Z6M1	RABEPK	1.306	0.047936803	Up	40.564	19.6	6
Q9UJ70	NAGK	0.576	0.010648367	Down	37.375	43.6	11
P31350	RRM2	0.597	0.004834361	Down	44.877	38.3	13
Q9UKK3	PARP4	1.456	0.000410485	Up	192.59	16.9	29
Q9Y446	PKP3	1.359	0.023567061	Up	87.081	25.7	17
Q99567	NUP88	0.687	0.000556183	Down	83.541	22.1	13
P61927	RPL37	1.629	0.031069805	Up	11.078	32	4
Q8N806	UBR7	0.656	0.01469358	Down	47.998	19.5	7
O95425	SVIL	1.663	0.010882805	Up	247.74	10.4	20
O75683	SURF6	1.88	3.9932E-05	Up	41.45	19.7	8
Q00059	TFAM	1.474	0.041767766	Up	29.096	28.5	8
Q9BRA2	TXNDC17	0.696	0.006886629	Down	13.941	36.6	4
Q9BVJ6	UTP14A	1.481	0.000437358	Up	87.977	23	17
Q9BYJ9	YTHDF1	0.723	0.002774338	Down	60.873	19.1	4
Q9UBN6	TNFRSF10D	1.368	0.029471403	Up	41.823	8.8	3
Q9Y3A2	UTP11	1.605	0.000923458	Up	30.446	24.5	7
P0DN76	U2AF1L5	0.742	0.002427345	Down	27.872	28.3	7
Q9P217	ZSWIM5	4.012	1.47116E-08	Up	130.63	1.6	2
Q9P260	RELCH	1.326	0.020650265	Up	134.63	8.2	8
P35579	MYH9	1.369	1.49483E-19	Up	226.53	41.1	64
P11047	LAMC1	0.751	0.000287789	Down	177.6	20.4	30
Q9BYG3	NIFK	1.35	0.009494407	Up	34.222	35.5	9
P28062	PSMB8	1.417	0.031735695	Up	30.354	29	7
P30041	PRDX6	0.131	0.000499515	Down	25.035	40.2	8
Q9UMS4	PRPF19	0.764	2.40908E-06	Down	55.18	34.9	13
O76021	RSL1D1	1.802	0.000176318	Up	54.972	32.4	15
Q03519	TAP2	2.19	0.000399746	Up	75.663	16.6	10
O00267	SUPT5H	0.737	0.000187748	Down	121	32.5	29
P23381	WARS1	1.895	0.001053422	Up	53.165	42.7	15
P13726	F3	0.671	0.012505785	Down	33.067	19.7	5
P10599	TXN	1.325	0.000480688	Up	11.737	48.6	6
Q3ZCQ8	TIMM50	0.555	0.003635533	Down	39.646	36.5	11
Q96J01	THOC3	0.732	0.000437152	Down	38.771	39.6	11
P11388	TOP2A	0.605	4.17725E-10	Down	174.38	34.9	39
Q96KR1	ZFR	0.644	0.013879962	Down	117.01	24.8	21
P36871	PGM1	1.353	2.98298E-17	Up	61.448	42.3	23
Q29RF7	PDS5A	0.757	8.65605E-06	Down	150.83	26.8	31
P27694	RPA1	0.741	0.002563158	Down	68.137	43.8	24
Q9BZK7	TBL1XR1	1.557	0.014841409	Up	55.594	37.9	10
Q14166	TTLL12	1.44	0.000342147	Up	74.403	33.2	16
Q14119	VEZF1	0.627	0.013325993	Down	56.931	12.7	5
P30408	TM4SF1	0.645	0.001983313	Down	21.632	4.5	1
P36873	PPP1CC	0.687	0.036201781	Down	36.983	37.2	2
Q9UBV2	SEL1L	1.541	0.040615344	Up	88.754	10.3	8
P08842	STS	0.483	0.000861993	Down	65.492	9.9	6
Q96T60	PNKP	0.75	0.000115896	Down	57.076	24.4	13
P28072	PSMB6	0.721	0.00167356	Down	25.357	23.4	6
Q9H6W3	RIOX1	0.7	9.45258E-06	Down	71.085	19.8	10

Q8IZQ5	SELENOH	0.527	0.004583606	Down	13.453	35.2	4
P62195	PSMC5	0.764	8.63345E-11	Down	45.626	60.1	21
Q01970	PLCB3	1.333	0.000186483	Up	138.8	19.3	19
Q6UW63	POGLUT2	0.74	0.006486466	Down	58.042	17.9	7
P98179	RBM3	1.66	0.020270026	Up	17.17	28.7	5
Q9NQG5	RPRD1B	0.704	0.028918185	Down	36.899	36.2	10
Q92541	RTF1	0.721	0.000551275	Down	80.313	23.8	17
Q96I25	RBM17	0.749	0.011689882	Down	44.961	33.2	13
P28065	PSMB9	2.497	0.00267514	Up	23.264	28.3	5
Q9NS91	RAD18	0.631	0.001547306	Down	56.222	9.9	5
Q7L4I2	RSRC2	0.765	0.038974576	Down	50.559	12.2	4
O00422	SAP18	0.723	5.3046E-05	Down	17.561	29.4	6
Q9NRY2	INIP	0.744	0.026338663	Down	11.425	24	2
Q8IX01	SUGP2	0.69	0.000548658	Down	120.21	25.1	23
Q9Y312	AAR2	0.744	0.003369415	Down	43.472	13.8	5
O60547	GMDS	1.442	3.356E-07	Up	41.949	35.2	13
Q05682	CALD1	1.564	0.008940741	Up	93.23	16.3	10
Q8IZR5	CMTM4	1.533	0.001067275	Up	25.827	8.1	2
Q99439	CNN2	2.02	0.000216243	Up	33.697	26.5	7
P25686	DNAJB2	0.637	0.030604846	Down	35.58	15.4	5
Q14244	MAP7	1.522	0.00717624	Up	84.051	3.5	3
P13798	APEH	1.355	8.28445E-07	Up	81.224	24.5	15
Q9BYM8	RBCK1	1.471	0.018667901	Up	57.571	17.6	8
P48506	GCLC	1.315	0.030423044	Up	72.765	22	12
P61769	B2M	2.755	0.004418089	Up	13.714	22.7	3
O43148	RNMT	0.746	0.01044095	Down	54.843	18.5	8
Q14566	MCM6	0.652	2.37533E-08	Down	92.888	36.3	29
P33993	MCM7	0.717	0.008259438	Down	81.307	33.4	21
Q6P9B6	MEAK7	1.449	0.016439042	Up	50.993	20.4	7
Q5JU85	IQSEC2	1.475	0.017549721	Up	162.78	3.3	3
Q14562	DHX8	0.678	0.00021858	Down	139.31	17.5	19
Q7Z460	CLASP1	1.392	0.004273034	Up	169.45	17	19
P09543	CNP	0.757	0.01427161	Down	47.578	36.1	15
P04083	ANXA1	1.364	8.59219E-05	Up	38.714	59.8	20
Q99700	ATXN2	0.716	0.009192111	Down	140.28	16.3	17
P04920	SLC4A2	1.401	0.004751428	Up	137.01	5	6
P09496	CLTA	0.659	1.67758E-08	Down	27.076	21.8	7
Q9NP61	ARFGAP3	0.747	3.2184E-05	Down	56.928	23.1	12
P02511	CRYAB	3.605	8.29464E-05	Up	20.159	23.4	4
Q96CT7	CCDC124	0.644	2.03688E-05	Down	25.835	33.2	9
Q9Y6D6	ARFGEF1	0.739	0.001149525	Down	208.76	15.5	18
P48509	CD151	1.358	0.04230193	Up	28.295	20.9	6
Q9NY33	DPP3	1.343	2.7324E-07	Up	82.588	29	16
Q6P6C2	ALKBH5	0.762	0.005284629	Down	44.255	16.2	7
P08236	GUSB	0.732	0.002159479	Down	74.731	9.2	6
Q96F63	CCDC97	0.702	0.01747439	Down	38.946	14.6	4
P16220	CREB1	0.747	0.022575093	Down	35.136	9.2	3
P18858	LIG1	0.704	0.021179242	Down	101.73	20	17
Q15067	ACOX1	0.571	8.82913E-08	Down	74.423	27.3	14
P08243	ASNS	1.38	0.000176769	Up	64.369	37.8	19
P07858	CTSB	0.379	6.39469E-05	Down	37.821	34.8	10
Q03135	CAV1	0.751	0.000536577	Down	20.471	28.7	6
Q86X55	CARM1	1.37	1.10188E-05	Up	65.853	20.2	12
P41250	GARS1	1.511	7.48015E-10	Up	83.165	29.5	22
P30260	CDC27	0.72	0.00010379	Down	91.866	19.1	15
O14757	CHEK1	0.574	0.000199193	Down	54.433	24.6	11
P50416	CPT1A	1.352	0.00274279	Up	88.367	16.3	12
Q9NR28	DIABLO	0.732	0.012793041	Down	27.131	20.5	5

O60610	DIAPH1	1.468	2.1954E-05	Up	141.35	22.8	29
O14641	DVL2	0.605	0.039635802	Down	78.947	9.1	6
Q8N8S7	ENAH	1.326	0.001445432	Up	66.509	20.1	10
P50613	CDK7	0.684	0.000442859	Down	39.038	28	9
Q14320	FAM50A	0.731	2.36022E-06	Down	40.241	30.4	12
P42126	ECI1	1.352	3.45176E-07	Up	32.816	26.2	8
P21589	NT5E	1.55	0.018833891	Up	63.367	24.6	11
Q9BRD0	BUD13	0.734	0.002513026	Down	70.52	11.8	7
P17174	GOT1	1.344	0.046587907	Up	46.247	48.2	16
Q96CM8	ACSF2	0.544	0.002283551	Down	68.124	17.6	9
P07384	CAPN1	1.427	5.59747E-11	Up	81.889	33.8	23
P49916	LIG3	0.678	3.37009E-08	Down	112.91	22.6	23
Q6PD62	CTR9	0.711	0.000840638	Down	133.5	15.3	18
Q9UII2	ATP5IF1	1.565	0.001404307	Up	12.249	14.2	2
P51946	CCNH	0.564	0.02357216	Down	37.643	20.1	6
P07199	CENPB	0.73	0.00337469	Down	65.171	6.2	3
Q92888	ARHGEF1	0.565	2.8671E-06	Down	102.43	26.2	23
Q92974	ARHGEF2	1.544	3.24956E-06	Up	111.54	27	22
Q99459	CDC5L	0.761	0.000475104	Down	92.25	24.2	15
P15121	AKR1B1	1.602	1.36728E-05	Up	35.853	37	13
Q9UQB8	BAIAP2	0.649	0.006354164	Down	60.867	32.6	17
Q96F07	CYFIP2	1.342	0.028780435	Up	148.4	15.3	5
Q8WYA6	CTNNBL1	0.724	0.043964643	Down	65.173	12.8	8
P00966	ASS1	1.734	0.00898011	Up	46.53	23.8	10
Q6DD88	ATL3	1.365	0.041152384	Up	60.541	31.1	15
P11021	HSPA5	1.374	8.26041E-26	Up	72.332	54.9	35
P28340	POLD1	0.695	8.62643E-05	Down	123.63	19.6	20
Q96JD6	AKR1E2	1.381	0.010552941	Up	36.589	2.5	1
P04626	ERBB2	0.653	1.22502E-08	Down	137.91	17.1	17
Q13111	CHAF1A	0.702	0.008724106	Down	106.91	11.4	11
Q9BVP2	GNL3	1.323	0.000193613	Up	61.992	18.6	9
P50502	ST13	1.432	2.75659E-05	Up	41.331	30.9	11
Q9NZ08	ERAP1	1.665	5.28889E-06	Up	107.23	14.9	14
P09382	LGALS1	1.504	2.45884E-09	Up	14.716	70.4	9
O95232	LUC7L3	0.722	0.000368708	Down	51.466	18.3	8
Q9BUT1	BDH2	0.572	0.000962789	Down	26.724	22	5
Q96DG6	CMBL	0.744	1.14189E-05	Down	28.048	25.7	7
P00403	MT-CO2	4.828	0.014716004	Up	25.565	8.8	2
Q68CQ7	GLT8D1	0.761	0.014586609	Down	41.935	10	4
P15586	GNS	0.693	0.013135362	Down	62.081	18.8	10
P04439	HLA-A	2.779	0.001633142	Up	40.84	44.1	10
Q8TED1	GPX8	0.656	0.011461711	Down	23.881	34.4	6
Q9HC38	GLOD4	0.746	1.79907E-07	Down	34.793	31.3	10
Q1KMD3	HNRNPUL2	0.586	2.2714E-11	Down	85.104	30	21
O00291	HIP1	1.31	0.019495415	Up	116.22	17.6	14
Q27J81	INF2	1.368	0.001551192	Up	135.62	14.7	17
Q9Y3D8	AK6	0.678	0.047829299	Down	20.061	25	4
Q9UK76	JPT1	0.575	0.020812155	Down	16.014	46.1	5
P38919	EIF4A3	0.724	0.014868995	Down	46.871	37.5	14
O60645	EXOC3	0.768	0.001839012	Down	85.566	23.6	15
O96005	CLPTM1	0.747	0.000554555	Down	76.096	24.4	13
Q14019	COTL1	1.311	4.63375E-07	Up	15.945	47.2	7
Q9BTC0	DIDO1	0.643	0.000764753	Down	243.87	12.5	24
O95466	FMNL1	1.405	0.01872559	Up	121.85	8.9	7
Q8IWX8	CHERP	0.762	9.2918E-06	Down	103.7	21.2	17
O75976	CPD	0.729	0.000400618	Down	152.93	22.9	26
P57737	CORO7	1.673	0.002788898	Up	100.6	7.6	6
P63218	GNG5	0.675	0.001022561	Down	7.3184	38.2	3

P33316	DUT	0.71	5.42508E-06	Down	26.563	46.4	10
Q16643	DBN1	1.368	3.14685E-06	Up	71.428	37.3	17
P04114	APOB	1.585	0.000990479	Up	515.6	2	8
Q8WUP2	FBLIM1	1.666	0.001427092	Up	40.669	18.8	7
Q15392	DHCR24	1.377	0.000140978	Up	60.101	14.1	8
Q9NZN4	EHD2	0.57	0.00206958	Down	61.161	27.8	14
Q8IWT0	ZBTB8OS	2.339	0.019737192	Up	19.491	15.6	3
P20073	ANXA7	1.404	8.90563E-06	Up	52.739	32.6	15
P08133	ANXA6	0.508	2.87183E-07	Down	75.872	49.2	30
P38432	COIL	0.572	0.030100281	Down	62.608	18.2	9
P49748	ACADVL	1.395	1.72792E-05	Up	70.389	39.2	23
P23786	CPT2	1.325	0.029279022	Up	73.776	28.3	16
P29317	EPHA2	1.395	0.016862838	Up	108.27	26.4	21
Q9Y223	GNE	0.704	0.000409791	Down	79.274	30.5	18
Q14344	GNA13	0.677	0.000475519	Down	44.049	29.7	10
Q01167	FOXK2	0.768	0.019203808	Down	69.061	6.2	4
Q02978	SLC25A11	0.574	0.000319455	Down	34.061	22.6	6
Q9BRK5	SDF4	0.696	2.48651E-06	Down	41.806	21.3	7
Q9H5V8	CDCP1	1.856	0.01162199	Up	92.931	14.5	11
Q9Y696	CLIC4	1.399	0.004689325	Up	28.772	39.5	9
Q9NVM9	INTS13	0.731	1.87919E-05	Down	80.224	23.2	16
Q08379	GOLGA2	0.709	0.000864385	Down	113.08	16.4	14
O60244	MED14	0.708	0.01767749	Down	160.6	4.3	6
Q14573	ITPR3	1.607	0.000211934	Up	304.1	12.6	26
P39748	FEN1	0.76	0.004280755	Down	42.592	32.4	10
Q9BQ67	GRWD1	0.695	0.001103606	Down	49.419	9	4
P0DMV9	HSPA1B	1.593	1.46007E-08	Up	70.051	47.4	10
P07942	LAMB1	0.549	3.81974E-06	Down	198.04	12.1	19
Q53F19	NCBP3	0.701	0.008498914	Down	70.592	14.7	8
O95983	MBD3	0.679	0.002026494	Down	32.844	13.7	3
O00139	KIF2A	0.757	8.80355E-05	Down	79.954	31	19
Q6P179	ERAP2	1.387	0.002635971	Up	110.46	23.1	23
Q14974	KPNB1	0.707	4.25434E-09	Down	97.169	34.9	27
P52292	KPNA2	0.559	0.000754785	Down	57.861	32.7	14
P27144	AK4	1.392	0.00031072	Up	25.268	54.7	9
Q3KQU3	MAP7D1	1.373	0.004360135	Up	92.819	11.8	8
P02751	FN1	2.796	0.000402748	Up	272.32	10.8	21
P32004	L1CAM	2.499	7.49683E-06	Up	140	17.3	17
O60443	GSDME	1.614	0.037319217	Up	54.554	25.4	11
Q9H1K1	ISCU	0.674	5.983E-05	Down	17.999	37.7	6
P20810	CAST	1.484	0.018531552	Up	76.572	26.8	14
P46013	MKI67	0.727	1.66179E-18	Down	358.69	38.3	97
P42167	TMPO	0.681	1.22976E-10	Down	50.67	42.7	9
Q9H3U5	MFSD1	1.346	0.001930372	Up	51.208	4.9	2
P55010	EIF5	1.482	1.90196E-07	Up	49.222	33.4	15
C9JLW8	MCRIP1	0.706	0.027149762	Down	10.92	40.2	4
Q8TAT6	NPLOC4	0.75	3.42182E-06	Down	68.119	37.8	20
O75694	NUP155	0.743	0.002229425	Down	155.2	16.8	21
P04792	HSPB1	1.641	1.57347E-11	Up	22.782	70.7	11
Q9H074	PAIP1	0.717	0.020546247	Down	53.524	22.1	11
P31323	PRKAR2B	0.708	0.030535178	Down	46.302	10.5	2
Q8TD19	NEK9	1.418	0.005349924	Up	107.17	9.3	8
P33991	MCM4	0.701	0.00346555	Down	96.557	37.2	34
P35580	MYH10	0.741	0.003718363	Down	229	29.4	38
Q15645	TRIP13	0.768	1.47175E-06	Down	48.55	30.6	12
Q9H936	SLC25A22	1.316	0.002988914	Up	34.47	33.4	10
P30085	CMPK1	1.323	0.031174984	Up	22.222	32.1	6
P50851	LRBA	1.363	0.000816725	Up	319.1	17	45

Q8ND56	LSM14A	0.723	0.011616778	Down	50.529	25.9	10
Q96PU5	NEDD4L	1.414	0.031192706	Up	111.93	13.6	10
Q9H4A6	GOLPH3	0.71	0.001823547	Down	33.81	21.8	6
Q6WCQ1	MPRIP	0.553	2.50782E-06	Down	116.53	22	19
Q9P2K5	MYEF2	1.66	0.0045796	Up	64.121	19.8	10
Q9NVZ3	NECAP2	1.378	0.043712369	Up	28.338	42.2	8
Q9UMY1	NOL7	1.416	0.004516138	Up	29.426	14	5
Q9BUJ2	HNRNPUL1	0.695	0.0003314	Down	95.737	29.7	21
P29218	IMPA1	0.769	0.000231663	Down	30.188	39.7	11
P07203	GPX1	0.602	0.015348655	Down	22.088	60.6	9
P11717	IGF2R	1.724	8.19138E-11	Up	274.37	18.2	40
P09960	LTA4H	0.657	3.92628E-12	Down	69.284	47.3	25
Q96FZ2	HMCES	0.661	0.000369655	Down	40.574	33.9	11
Q13325	IFIT5	0.491	0.006901063	Down	55.846	12.2	4
P37268	FDFT1	1.595	0.000939898	Up	48.115	35	13
O75367	MACROH2A1	0.656	4.52888E-07	Down	39.617	44.1	14
P01130	LDLR	1.539	0.009357399	Up	95.375	18.5	15
O60341	KDM1A	0.717	0.015862257	Down	92.902	32.6	19
P29084	GTF2E2	0.761	0.00276266	Down	33.043	42.6	11
P13984	GTF2F2	0.749	0.001073573	Down	28.38	47.8	13
Q13263	TRIM28	0.745	2.6715E-09	Down	88.549	55.8	29
Q9Y6A5	TACC3	0.627	0.017896832	Down	90.359	21.5	14
Q16831	UPP1	1.624	1.37152E-06	Up	33.934	44.2	14
Q9Y613	FHOD1	1.365	0.006877502	Up	126.55	24.4	23
O94992	HEXIM1	0.574	0.008987891	Down	40.623	18.1	5
P54577	YARS1	1.454	9.8732E-10	Up	59.143	54.9	29
Q5T160	RARS2	1.452	0.019343252	Up	65.505	15.2	9
Q13426	XRCC4	0.648	0.004472819	Down	38.286	31.5	10
Q9UGI8	TES	1.396	0.001457853	Up	47.996	51.1	20
P53999	SUB1	0.693	1.35996E-06	Down	14.395	35.4	5
Q86V81	ALYREF	0.713	0.000148221	Down	26.888	35.8	7
Q12888	TP53BP1	0.678	6.30116E-11	Down	213.57	32.3	46
O75717	WDHD1	0.683	0.000343029	Down	125.97	17.9	17
Q9C0C9	UBE2O	0.671	0.034796038	Down	141.29	25.7	27
O60568	PLOD3	1.319	0.015419562	Up	84.784	17.6	11
P25205	MCM3	0.765	3.69354E-05	Down	90.98	47.9	38
Q8WX93	PALLD	1.664	0.039898325	Up	150.56	11.6	13
Q9BZQ8	NIBAN1	2.23	1.78066E-06	Up	103.13	17.7	15
P22392	NME2	0.676	4.22001E-05	Down	17.298	58.6	1
P07237	P4HB	0.742	1.11477E-25	Down	57.116	51.4	28
Q96BN8	OTULIN	0.721	0.003347768	Down	40.262	21	9
P33992	MCM5	0.723	0.000810183	Down	82.285	45	31
Q9H832	UBE2Z	0.747	2.75036E-09	Down	38.21	35.3	12
Q7Z2W4	ZC3HAV1	1.311	3.82633E-08	Up	101.43	36.7	30
P38435	GGCX	0.738	0.017496863	Down	87.56	8.6	8
O15213	WDR46	1.674	0.012217865	Up	68.07	20.5	10
P52565	ARHGDIA	0.759	0.001459736	Down	23.207	45.1	8

Table S3. Potential E3 ubiquitin ligases of NNMT identified by MS analysis.

Protein accession	Protein name	MW [kDa]	Coverage [%]	Peptides	Unique peptides	Ratio
Q86YT6	MIB1	110.13586	9.2	7	6	3.422880575
Q9BRZ2	TRIM56	81.4877	18.5	11	11	1.786284139
Q14258	TRIM25	70.9734	18.4	9	9	1.648763113

Table S4. Differentially expressed genes in *PRDX6* KO SKOV3 cells identified by RNA-seq analysis.

gene_id	log2FoldChange	pvalue	gene_name
ENSG00000169855	-12.33083185	3.14E-32	ROBO1
ENSG00000169851	-11.98577205	8.38E-30	PCDH7
ENSG00000196335	6.000443324	9.96E-28	STK31
ENSG00000166415	-11.38894424	1.15E-25	WDR72
ENSG00000196549	-6.839159666	3.25E-25	MME
ENSG00000115414	-4.641422734	3.26E-22	FN1
ENSG00000198796	-5.075410692	1.24E-21	ALPK2
ENSG00000114200	-10.76285427	1.66E-21	BCHE
ENSG00000166825	-7.796513452	4.45E-21	ANPEP
ENSG00000198774	-10.6837309	5.03E-21	RASSF9
ENSG00000261122	-4.621604521	1.15E-20	LINC02167
ENSG00000124882	-5.571055467	9.03E-20	EREG
ENSG00000184226	-4.414554338	8.41E-19	PCDH9
ENSG00000111058	-10.07685962	3.00E-17	ACSS3
ENSG00000250337	-9.983839265	9.96E-17	PURPL
ENSG00000091592	-4.024632747	3.14E-16	NLRP1
ENSG00000081803	-5.494002417	1.13E-15	CADPS2
ENSG00000099399	-4.853187076	2.92E-15	MAGEB2
ENSG00000000971	-6.712276777	1.73E-14	CFH
ENSG00000173432	-3.553450762	4.88E-14	SAA1
ENSG00000150630	-9.503776589	5.19E-14	VEGFC
ENSG00000172058	-3.825509004	8.58E-14	SERF1A
ENSG00000134339	-3.730588806	1.59E-13	SAA2
ENSG00000164825	-9.363117859	2.65E-13	DEFB1
ENSG00000253522	-4.504104724	3.19E-13	MIR3142HG
ENSG00000206532	-4.29736265	5.03E-13	AC117402.1
ENSG00000128242	-5.686588417	6.28E-13	GAL3ST1
ENSG00000215808	-9.287287421	7.79E-13	LINC01139
ENSG00000137491	-5.23002644	1.28E-12	SLCO2B1
ENSG00000080293	-6.141511224	1.94E-11	SCTR
ENSG00000171227	-3.142821702	2.50E-11	TMEM37
ENSG00000188176	-3.286392024	3.96E-11	SMTNL2
ENSG00000271503	-8.885930962	5.84E-11	CCL5
ENSG00000174607	-3.122030164	7.49E-11	UGT8
ENSG00000180318	-8.8599912	9.92E-11	ALX1
ENSG00000019582	-4.02898295	1.19E-10	CD74
ENSG00000134115	-3.183997401	1.30E-10	CNTN6
ENSG00000111885	-3.511561266	1.90E-10	MAN1A1
ENSG00000109819	-3.289963412	1.93E-10	PPARGC1A
ENSG00000170558	-3.973013392	2.23E-10	CDH2
ENSG00000137648	2.935426589	2.73E-10	TMPRSS4
ENSG00000008394	-3.196386154	3.03E-10	MGST1
ENSG00000049089	-3.899852556	5.00E-10	COL9A2
ENSG00000160179	4.942138177	6.07E-10	ABCG1
ENSG00000124762	2.865436546	8.62E-10	CDKN1A
ENSG00000137752	-8.6028008	1.25E-09	CASP1
ENSG00000089127	-2.991149198	1.29E-09	OAS1
ENSG00000168268	-3.505119292	1.87E-09	NT5DC2
ENSG00000235142	-4.989044327	1.93E-09	LINC02532
ENSG00000164761	-2.960788187	1.95E-09	TNFRSF11B
ENSG00000111863	2.725347408	2.13E-09	ADTRP
ENSG00000213401	-8.538839128	2.27E-09	MAGEA12
ENSG00000131409	-4.547477178	3.42E-09	LRRC4B
ENSG00000131831	2.883253657	4.83E-09	RAI2
ENSG00000137673	-2.543805655	5.82E-09	MMP7
ENSG00000163395	-2.49546026	9.09E-09	IGFN1
ENSG00000107159	5.395065099	9.38E-09	CA9
ENSG00000197172	-3.025906926	1.03E-08	MAGEA6

ENSG00000261742	-8.36530696	1.09E-08	LINC00922
ENSG00000174827	-2.62371367	1.85E-08	PDZK1
ENSG00000137699	2.512894753	2.08E-08	TRIM29
ENSG00000168843	-8.289594567	2.10E-08	FSTL5
ENSG00000159164	-3.272404923	2.53E-08	SV2A
ENSG00000285972	-4.080660534	2.89E-08	CERNA2
ENSG00000079215	-5.399450503	3.96E-08	SLC1A3
ENSG00000135480	2.374404487	4.01E-08	KRT7
ENSG00000162591	2.445138613	4.02E-08	MEGF6
ENSG00000133019	-8.209687657	4.11E-08	CHRM3
ENSG00000135298	-8.209687657	4.11E-08	ADGRB3
ENSG00000047457	-2.548346891	4.25E-08	CP
ENSG00000187837	2.403257265	4.42E-08	HIST1H1C
ENSG00000171004	-2.78048366	6.00E-08	HS6ST2
ENSG00000256518	3.616824977	7.14E-08	AP000812.3
ENSG00000114541	-2.822347951	7.89E-08	FRMD4B
ENSG00000169903	-3.790614818	8.40E-08	TM4SF4
ENSG00000148346	-2.358130412	8.99E-08	LCN2
ENSG00000107984	2.988907567	1.15E-07	DKK1
ENSG00000136859	8.062922585	1.17E-07	ANGPTL2
ENSG00000115919	-8.125094074	1.17E-07	KYNU
ENSG00000002587	-2.620052993	1.28E-07	HS3ST1
ENSG00000154736	2.42990874	1.46E-07	ADAMTS5
ENSG00000123977	2.99878063	1.49E-07	DAW1
ENSG00000232850	-4.176269484	1.51E-07	PTGES2-AS1
ENSG00000279516	-4.55115534	1.52E-07	FAM230C
ENSG00000100033	-3.721483417	1.60E-07	PRODH
ENSG00000131242	2.328400724	1.65E-07	RAB11FIP4
ENSG00000165379	-8.080861429	1.67E-07	LRFN5
ENSG00000176978	-2.429245932	1.67E-07	DPP7
ENSG00000158089	-2.388442578	1.87E-07	GALNT14
ENSG00000197705	-2.418168943	2.01E-07	KLHL14
ENSG00000102312	2.243963837	2.33E-07	PORCN
ENSG00000181143	7.978360621	2.40E-07	MUC16
ENSG00000148600	-3.673484045	2.49E-07	CDHR1
ENSG00000135447	-5.183282598	2.51E-07	PPP1R1A
ENSG00000115008	2.230128076	2.57E-07	IL1A
ENSG00000278817	2.996794392	2.63E-07	AC007325.4
ENSG00000196593	2.253234762	2.67E-07	ANKRD20A19P
ENSG00000273802	4.328199025	2.67E-07	HIST1H2BG
ENSG00000214814	4.328199025	2.67E-07	FER1L6
ENSG00000248588	3.085619237	3.18E-07	AC008517.1
ENSG00000272323	3.915881274	3.28E-07	AC026801.2
ENSG00000056291	2.483877588	3.31E-07	NPFFR2
ENSG00000108691	-7.988107204	3.48E-07	CCL2
ENSG00000135114	-2.426038075	3.77E-07	OASL
ENSG00000154639	-2.388069026	4.18E-07	CXADR
ENSG00000128965	2.217945866	4.74E-07	CHAC1
ENSG00000137959	-5.103375688	4.78E-07	IFI44L
ENSG00000177464	-3.24664863	4.95E-07	GPR4
ENSG00000120915	-7.939393527	5.06E-07	EPHX2
ENSG00000257671	2.54443757	5.42E-07	KRT7-AS
ENSG00000162896	-4.027769953	5.57E-07	PIGR
ENSG00000205978	-7.888977343	7.40E-07	NYNRIN
ENSG00000135374	-2.939736255	7.58E-07	ELF5
ENSG00000178662	-2.491854873	7.76E-07	CSRNP3
ENSG00000154274	-2.662892402	8.45E-07	C4orf19
ENSG00000139970	2.789824946	8.60E-07	RTN1
ENSG00000130054	-3.103783267	9.00E-07	FAM155B
ENSG00000011465	-2.630066673	9.99E-07	DCN
ENSG00000075213	2.152492185	1.01E-06	SEMA3A

ENSG00000157388	-2.329395909	1.03E-06	CACNA1D
ENSG00000253313	4.166406256	1.16E-06	C1orf210
ENSG00000172889	-2.163041698	1.21E-06	EGFL7
ENSG00000134321	-2.331544625	1.34E-06	RSAD2
ENSG00000168497	-2.196968842	1.39E-06	CAVIN2
ENSG00000162654	-4.269575505	1.58E-06	GBP4
ENSG00000212916	-7.782530243	1.61E-06	MAP10
ENSG00000250102	-7.782530243	1.61E-06	LINC02377
ENSG00000103257	2.038333876	1.73E-06	SLC7A5
ENSG00000271447	2.477797824	1.74E-06	MMP28
ENSG00000185885	-2.213705066	1.89E-06	IFITM1
ENSG00000091972	2.755809216	1.93E-06	CD200
ENSG00000125910	3.281756667	1.98E-06	S1PR4
ENSG00000131203	-3.20685519	1.98E-06	IDO1
ENSG00000284823	3.023894449	2.45E-06	AL356747.1
ENSG00000146678	-3.435218164	2.52E-06	IGFBP1
ENSG00000221867	-2.672726839	2.60E-06	MAGEA3
ENSG00000278965	2.873631458	2.73E-06	AC122713.2
ENSG00000244468	-2.409082173	2.74E-06	AC093001.1
ENSG00000131378	-2.959678739	3.15E-06	RFTN1
ENSG00000150471	-7.667598954	3.61E-06	ADGRL3
ENSG00000115155	-4.881795234	3.68E-06	OTOF
ENSG00000162493	-4.881795234	3.68E-06	PDPN
ENSG00000259674	-3.790276112	3.89E-06	AC092868.1
ENSG00000254290	-3.790276112	3.89E-06	AC124067.4
ENSG00000163762	-2.809202236	4.33E-06	TM4SF18
ENSG00000049130	-2.026725809	4.50E-06	KITLG
ENSG00000255860	2.819947833	4.59E-06	AP000812.2
ENSG00000204262	2.022830347	4.95E-06	COL5A2
ENSG00000153071	-7.606506887	5.45E-06	DAB2
ENSG00000170962	-7.606506887	5.45E-06	PDGFD
ENSG00000203727	-2.229048092	5.77E-06	SAMD5
ENSG00000237330	3.144596889	6.75E-06	RNF223
ENSG00000010379	-2.295340178	6.88E-06	SLC6A13
ENSG00000237438	-2.288756405	7.77E-06	CECR7
ENSG00000112541	2.022965028	7.89E-06	PDE10A
ENSG00000148143	-7.542712994	8.28E-06	ZNF462
ENSG00000277701	-2.493628985	8.51E-06	AC159540.2
ENSG00000185745	-2.015142659	9.18E-06	IFIT1
ENSG00000172062	1.938055466	9.33E-06	SMN1
ENSG00000276600	-2.766864367	9.46E-06	RAB7B
ENSG00000226578	-2.716270761	9.47E-06	AL132657.1
ENSG00000156453	1.943982114	1.04E-05	PCDH1
ENSG00000117228	-4.060842367	1.06E-05	GBP1
ENSG00000147155	2.016869114	1.06E-05	EBP
ENSG00000227234	-4.730423356	1.09E-05	SPANXB1
ENSG00000258932	-4.730423356	1.09E-05	AL390334.1
ENSG00000156140	-2.460122646	1.14E-05	ADAMTS3
ENSG00000168427	2.954953791	1.17E-05	KLHL30
ENSG00000134198	-2.477452531	1.17E-05	TSPAN2
ENSG00000111335	-1.993709392	1.20E-05	OAS2
ENSG00000118308	7.460013574	1.27E-05	LRMP
ENSG00000187546	-7.475967196	1.27E-05	AGMO
ENSG00000270880	7.460013574	1.27E-05	AC128687.1
ENSG00000116711	-7.475967196	1.27E-05	PLA2G4A
ENSG00000206538	-7.475967196	1.27E-05	VGLL3
ENSG00000133640	-7.475967196	1.27E-05	LRRIQ1
ENSG00000164099	-2.023510033	1.32E-05	PRSS12
ENSG00000104870	-2.324204101	1.32E-05	FCGRT
ENSG00000179583	-2.127020251	1.39E-05	CIITA
ENSG00000135905	-2.799566519	1.46E-05	DOCK10

ENSG00000136574	2.245119077	1.51E-05	GATA4
ENSG00000198121	-2.033795512	1.57E-05	LPAR1
ENSG00000271743	4.505093263	1.58E-05	AF287957.1
ENSG00000149131	-2.061022301	1.73E-05	SERPING1
ENSG00000101255	1.825808019	1.76E-05	TRIB3
ENSG00000165300	-2.840386042	1.86E-05	SLITRK5
ENSG00000137731	-1.989490953	1.91E-05	FXYD2
ENSG00000116761	1.850916712	1.94E-05	CTH
ENSG00000228741	7.396255635	1.96E-05	SPATA13
ENSG00000231013	-7.405982997	1.96E-05	AC013275.1
ENSG00000203685	-7.405982997	1.96E-05	STUM
ENSG00000274641	2.711157967	2.01E-05	HIST1H2BO
ENSG00000116299	-2.616938408	2.11E-05	KIAA1324
ENSG00000121236	-1.977270793	2.13E-05	TRIM6
ENSG00000180596	2.058410688	2.14E-05	HIST1H2BC
ENSG00000259230	4.452875358	2.30E-05	LINC02323
ENSG00000127329	1.808850471	2.78E-05	PTPRB
ENSG00000197355	-1.851069835	2.86E-05	UAP1L1
ENSG00000158406	1.967895744	2.94E-05	HIST1H4H
ENSG00000006210	-2.326509886	3.00E-05	CX3CL1
ENSG00000073067	-7.332430058	3.05E-05	CYP2W1
ENSG00000147168	7.329549194	3.05E-05	IL2RG
ENSG00000102349	7.329549194	3.05E-05	KLF8
ENSG00000187736	7.329549194	3.05E-05	NHEJ1
ENSG00000273507	-7.332430058	3.05E-05	AL354809.1
ENSG00000041982	-1.922462455	3.06E-05	TNC
ENSG00000169429	1.769567631	3.30E-05	CXCL8
ENSG00000271737	4.398696233	3.37E-05	AC008608.2
ENSG00000155254	-2.43501424	3.71E-05	MARVELD1
ENSG00000103355	2.426996746	3.75E-05	PRSS33
ENSG00000167306	1.777865118	3.75E-05	MYO5B
ENSG00000113212	-2.96131733	3.89E-05	PCDHB7
ENSG00000135697	-2.96131733	3.89E-05	BCO1
ENSG00000173237	-3.868958281	4.06E-05	C11orf86
ENSG00000110002	-3.868958281	4.06E-05	VWA5A
ENSG00000258088	-3.077726988	4.11E-05	AC078820.1
ENSG00000165949	-1.860104525	4.26E-05	IFI27
ENSG00000128536	-3.234831111	4.27E-05	CDHR3
ENSG00000223760	-2.35954336	4.41E-05	MED15P9
ENSG00000103316	-2.071084025	4.43E-05	CRYM
ENSG00000250584	1.810529627	4.43E-05	LINC01511
ENSG00000139209	-7.254924762	4.78E-05	SLC38A4
ENSG00000173227	-7.254924762	4.78E-05	SYT12
ENSG00000198930	-7.254924762	4.78E-05	CSAG1
ENSG00000259129	-7.254924762	4.78E-05	LINC00648
ENSG00000261115	-2.432158376	4.87E-05	TMEM178B
ENSG00000241935	-2.249057658	4.93E-05	HOGA1
ENSG00000181126	1.846575369	5.19E-05	HLA-V
ENSG00000124657	2.893778086	5.36E-05	OR2B6
ENSG00000104369	1.951499233	5.51E-05	JPH1
ENSG00000198795	3.634085058	5.75E-05	ZNF521
ENSG00000227014	-3.460198764	5.89E-05	AC007285.1
ENSG00000153233	1.861331306	6.11E-05	PTPRR
ENSG00000133106	-1.965675955	6.18E-05	EPSTI1
ENSG00000263155	1.773798534	6.37E-05	MYZAP
ENSG00000267128	-2.365882839	6.37E-05	RNF157-AS1
ENSG00000172458	2.321013666	6.53E-05	IL17D
ENSG00000005108	1.990305333	6.57E-05	THSD7A
ENSG00000029534	1.698161727	7.19E-05	ANK1
ENSG00000061918	-1.798080023	7.20E-05	GUCY1B1
ENSG00000285694	4.283823317	7.35E-05	AL162718.2

ENSG00000226887	-4.436401025	7.35E-05	ERVMER34-1
ENSG00000205133	-4.436401025	7.35E-05	TRIQK
ENSG00000203883	-4.436401025	7.35E-05	SOX18
ENSG00000262898	7.186103117	7.55E-05	AC139099.2
ENSG00000169245	-7.173018112	7.55E-05	CXCL10
ENSG00000143341	-2.017008847	8.07E-05	HMCN1
ENSG00000221970	3.217712085	8.10E-05	OR2A1
ENSG00000231574	-3.762511181	8.17E-05	LINC02015
ENSG00000260953	3.58369133	8.17E-05	AC009093.4
ENSG00000042062	-2.857278318	8.24E-05	RIPOR3
ENSG00000244274	2.798119677	9.35E-05	DBNDD2
ENSG00000101443	-1.813886352	9.42E-05	WFDC2
ENSG00000254166	-1.704620823	9.86E-05	CASC19
ENSG00000166147	1.762466166	0.000100203	FBN1
ENSG00000175874	1.875285276	0.000103513	CREG2
ENSG00000174837	-1.793994882	0.000104426	ADGRE1
ENSG00000106927	-2.225468646	0.000105987	AMBP
ENSG00000118513	-4.369655227	0.000109596	MYB
ENSG00000137878	1.672323891	0.000109882	GCOM1
ENSG00000132530	-3.706189724	0.000116677	XAF1
ENSG00000279971	7.108650889	0.000120499	AC020910.6
ENSG00000271380	7.108650889	0.000120499	AL451085.2
ENSG00000273062	-7.086179965	0.000120499	AL449106.1
ENSG00000273056	7.108650889	0.000120499	AL354694.1
ENSG00000183785	-7.086179965	0.000120499	TUBA8
ENSG00000151364	-7.086179965	0.000120499	KCTD14
ENSG00000169760	-7.086179965	0.000120499	NLGN1
ENSG00000105929	-7.086179965	0.000120499	ATP6V0A4
ENSG00000124429	-7.086179965	0.000120499	POF1B
ENSG00000178235	-7.086179965	0.000120499	SLITRK1
ENSG00000170899	-2.922107686	0.000121298	GSTA4
ENSG00000145287	-2.090670514	0.000125296	PLAC8
ENSG00000126709	-1.690770922	0.000130597	IFI6
ENSG00000125872	-1.688729928	0.000131301	LRRN4
ENSG00000117525	1.604912934	0.000136873	F3
ENSG00000023892	-2.783502236	0.000137515	DEF6
ENSG00000249857	2.903575181	0.000137915	AC027338.2
ENSG00000248498	2.945236689	0.000137915	ASNSP1
ENSG00000163071	-3.106004883	0.000137915	SPATA18
ENSG00000149922	-2.395054164	0.000138966	TBX6
ENSG00000240694	-1.795272009	0.000140526	PNMA2
ENSG00000235781	2.250435363	0.000142756	LINC02569
ENSG00000204936	2.024116569	0.000145175	CD177
ENSG00000196611	-2.060418914	0.000145175	MMP1
ENSG00000130287	-2.182476055	0.000146896	NCAN
ENSG00000259863	2.165799823	0.000150551	SH3RF3-AS1
ENSG00000184194	1.770997662	0.000150863	GPR173
ENSG00000162511	-1.86183919	0.000151828	LAPTM5
ENSG00000206190	1.755131662	0.000151922	ATP10A
ENSG00000101280	3.124995876	0.000154712	ANGPT4
ENSG00000251685	-3.313946496	0.000154712	UGT2B27P
ENSG00000050438	1.83201061	0.00016434	SLC4A8
ENSG00000249306	-4.299671028	0.000164371	LINC01411
ENSG00000100968	-4.299671028	0.000164371	NFATC4
ENSG00000235385	3.4772943	0.000167283	LINC02154
ENSG00000081052	1.611227621	0.000168887	COL4A4
ENSG00000136689	2.229414065	0.000169373	IL1RN
ENSG00000112394	-2.364756185	0.000172494	SLC16A10
ENSG00000131016	1.583398965	0.000174626	AKAP12
ENSG00000257150	2.601671209	0.000178139	PGAM1P5
ENSG00000178814	-2.081587127	0.00018149	OPLAH

ENSG00000124635	1.700272956	0.000182673	HIST1H2BJ
ENSG00000183615	-3.060373065	0.000186219	FAM167B
ENSG00000273760	2.311645894	0.000192772	AC245041.1
ENSG00000184557	-1.613914437	0.000192843	SOCS3
ENSG00000263276	-6.993778219	0.000193974	AC020978.7
ENSG00000146221	7.026803503	0.000193974	TCTE1
ENSG00000275930	7.026803503	0.000193974	RF00019
ENSG00000175877	-6.993778219	0.000193974	TMEM270
ENSG00000235328	-6.993778219	0.000193974	AC006946.1
ENSG00000207370	-6.993778219	0.000193974	RF00019
ENSG00000198185	-6.993778219	0.000193974	ZNF334
ENSG00000196862	-6.993778219	0.000193974	RGPD4
ENSG00000164176	-6.993778219	0.000193974	EDIL3
ENSG00000278530	-6.993778219	0.000193974	CHMP1B2P
ENSG00000118596	-6.993778219	0.000193974	SLC16A7
ENSG00000118946	-6.993778219	0.000193974	PCDH17
ENSG00000140015	-6.993778219	0.000193974	KCNH5
ENSG00000187867	-6.993778219	0.000193974	PALM3
ENSG00000231741	-2.386695877	0.000204856	AL353743.3
ENSG00000281327	1.716081946	0.000206032	LINC01338
ENSG00000004799	-1.663833072	0.000207669	PDK4
ENSG00000185112	1.630347693	0.000209819	FAM43A
ENSG00000279117	-1.589940557	0.000210275	AP001972.5
ENSG00000137965	-1.863144279	0.00021354	IFI44
ENSG00000258099	3.076303165	0.000214972	ATXN2-AS
ENSG00000172828	-1.711411152	0.000218078	CES3
ENSG00000165821	-2.487779511	0.000231229	SALL2
ENSG00000223485	3.421000868	0.000240916	LINC01615
ENSG00000249631	-3.586487825	0.000240916	AC005699.1
ENSG00000106327	-3.586487825	0.000240916	TFR2
ENSG00000145850	-1.98270392	0.000243382	TIMD4
ENSG00000185052	2.033171224	0.000244362	SLC24A3
ENSG00000262678	-4.226118088	0.000248027	AC004771.5
ENSG00000186867	-4.226118088	0.000248027	QRFPR
ENSG00000183486	-4.226118088	0.000248027	MX2
ENSG00000067798	-4.226118088	0.000248027	NAV3
ENSG00000182489	2.816459366	0.000252157	XKRX
ENSG00000253972	2.816459366	0.000252157	MAL2-AS1
ENSG00000187134	-1.588290349	0.000253853	AKR1C1
ENSG00000183023	1.568023595	0.000274415	SLC8A1
ENSG00000249992	2.324505409	0.000277866	TMEM158
ENSG00000050030	2.324505409	0.000277866	NEXMIF
ENSG00000189184	-2.837514102	0.000280171	PCDH18
ENSG00000101680	2.043046427	0.000283294	LAMA1
ENSG00000146049	-2.056849728	0.000284129	KAAG1
ENSG00000144802	-1.531430319	0.000284794	NFKBIZ
ENSG00000135821	-1.821482771	0.000286087	GLUL
ENSG00000065534	-1.542388035	0.000297475	MYLK
ENSG00000231728	3.025909438	0.000299755	TMSB15B-AS1
ENSG00000180767	-3.207499395	0.000299755	CHST13
ENSG00000229867	-3.207499395	0.000299755	STEAP3-AS1
ENSG00000227799	2.529757713	0.000300744	AC012358.2
ENSG00000134516	2.529757713	0.000300744	DOCK2
ENSG00000234290	-2.665242515	0.000300744	AC116366.1
ENSG00000213759	1.719836926	0.00030116	UGT2B11
ENSG00000019186	6.94003197	0.000315208	CYP24A1
ENSG00000271857	-6.895050874	0.000315208	AL096865.1
ENSG00000270765	-6.895050874	0.000315208	GAS2L2
ENSG00000285766	6.94003197	0.000315208	AC092042.4
ENSG00000259437	6.94003197	0.000315208	AC093334.1
ENSG00000279375	-6.895050874	0.000315208	AC244517.7

ENSG00000228262	-6.895050874	0.000315208	LINC01320
ENSG00000125730	-1.510965358	0.000315681	C3
ENSG00000116717	1.561766138	0.000324428	GADD45A
ENSG00000138670	2.517171509	0.000325735	RASGEF1B
ENSG00000160111	-2.269846574	0.000332559	CPAMD8
ENSG00000100867	1.504122383	0.000341911	DHRS2
ENSG00000229368	-2.964536981	0.000342416	AC090587.2
ENSG00000169169	-2.964536981	0.000342416	CPT1C
ENSG00000106633	3.362421384	0.000348534	GCK
ENSG00000275710	-3.522693933	0.000348534	AC007485.1
ENSG00000171509	1.50605368	0.000351928	RXFP1
ENSG00000214922	1.62961404	0.000353051	HLA-F-AS1
ENSG00000139910	-1.690142226	0.000355742	NOVA1
ENSG00000248112	1.649690075	0.000359214	AC108174.1
ENSG00000157087	2.589981623	0.000372105	ATP2B2
ENSG00000166278	-2.793281457	0.000372105	C2
ENSG00000101049	-1.542817419	0.000372916	SGK2
ENSG00000073282	4.022358912	0.000376589	TP63
ENSG00000273542	4.022358912	0.000376589	HIST1H4K
ENSG00000275793	-4.148612793	0.000376589	RIMBP3
ENSG00000188626	-4.148612793	0.000376589	GOLGA8M
ENSG00000251580	2.492410463	0.000391938	LINC02482
ENSG00000091262	-2.405596044	0.000391938	ABCC6
ENSG00000138615	2.492410463	0.000391938	CILP
ENSG00000248092	1.75625904	0.000392038	NNT-AS1
ENSG00000180573	1.500468542	0.000393569	HIST1H2AC
ENSG00000166347	-1.76535092	0.000397992	CYB5A
ENSG00000130513	1.490610095	0.000402916	GDF15
ENSG00000215097	2.484845474	0.000411667	DUSP8P3
ENSG00000204767	2.973691532	0.000419448	INSYN2B
ENSG00000255176	-3.151177939	0.000419448	AP000941.1
ENSG00000205436	2.973691532	0.000419448	EXOC3L4
ENSG00000272914	-3.151177939	0.000419448	AL359532.1
ENSG00000156113	-1.625597032	0.00043268	KCNMA1
ENSG00000115616	1.597990031	0.000434191	SLC9A2
ENSG00000141448	1.575337151	0.000444156	GATA6
ENSG00000099251	-1.63233202	0.000462239	HSD17B7P2
ENSG00000153822	-1.498132772	0.000465188	KCNJ16
ENSG00000260792	-2.914120797	0.000466301	LINC02280
ENSG00000281912	2.547081166	0.000495363	LINC01144
ENSG00000277443	-1.585536401	0.000496101	MARCKS
ENSG00000265828	3.301362289	0.000506526	MIR3939
ENSG00000261633	3.301362289	0.000506526	AC018552.3
ENSG00000280639	3.301362289	0.000506526	LINC02204
ENSG00000187608	-1.507299203	0.00051066	ISG15
ENSG00000233818	2.454070649	0.000511792	AP000695.2
ENSG00000256006	-2.580648932	0.000511792	AC084117.1
ENSG00000135604	-2.362679315	0.000511792	STX11
ENSG00000277938	-2.580648932	0.000511792	AL035252.3
ENSG00000182578	6.847705649	0.000517265	CSF1R
ENSG00000264235	-6.789067794	0.000517265	AP005329.1
ENSG00000099937	6.847705649	0.000517265	SERPIND1
ENSG00000235724	6.847705649	0.000517265	AC009299.3
ENSG00000227591	-6.789067794	0.000517265	AL031316.1
ENSG00000183773	-6.789067794	0.000517265	AIFM3
ENSG00000229956	-6.789067794	0.000517265	ZRANB2-AS2
ENSG00000151789	-6.789067794	0.000517265	ZNF385D
ENSG00000205403	-6.789067794	0.000517265	CFI
ENSG00000164116	-6.789067794	0.000517265	GUCY1A1
ENSG00000152402	-6.789067794	0.000517265	GUCY1A2
ENSG00000135100	-6.789067794	0.000517265	HNF1A

ENSG00000243225	-2.298460457	0.000519431	AC007686.1
ENSG00000145536	-2.246240365	0.000521035	ADAMTS16
ENSG00000136010	2.451778485	0.000521035	ALDH1L2
ENSG00000087076	-2.246240365	0.000521035	HSD17B14
ENSG00000112715	1.452298473	0.000533959	VEGFA
ENSG00000210191	-1.884517058	0.000537939	MT-TL2
ENSG00000234685	-1.546044842	0.000559688	NUS1P2
ENSG00000210174	-1.79680144	0.000564393	MT-TR
ENSG00000154165	-1.531705534	0.000569563	GPR15
ENSG00000205444	3.948853765	0.000575412	KU-MEL-3
ENSG00000174945	-4.066706143	0.000575412	AMZ1
ENSG00000272308	-4.066706143	0.000575412	AC104113.1
ENSG00000182264	-4.066706143	0.000575412	IZUMO1
ENSG00000225075	-4.066706143	0.000575412	AL603832.1
ENSG00000138798	-4.066706143	0.000575412	EGF
ENSG00000155465	-1.478692234	0.00058052	SLC7A7
ENSG00000188051	1.761982516	0.000587561	TMEM221
ENSG00000189334	1.761982516	0.000587561	S100A14
ENSG00000225544	-3.092568107	0.000589	AC245452.2
ENSG00000115457	-3.092568107	0.000589	IGFBP2
ENSG00000102032	-3.092568107	0.000589	RENBP
ENSG00000237916	-3.092568107	0.000589	AC011753.4
ENSG00000108602	1.519031008	0.000629512	ALDH3A1
ENSG00000148965	-2.861878779	0.000636793	SAA4
ENSG00000178752	1.454330793	0.000636966	ERFE
ENSG00000168743	-1.449510556	0.000638413	NPNT
ENSG00000159217	2.327534698	0.00064876	IGF2BP1
ENSG00000182459	-1.528653108	0.000650018	TEX19
ENSG00000227199	2.502865808	0.000660977	ST7-AS1
ENSG00000272980	-2.700527232	0.000660977	Z94721.2
ENSG00000135063	1.758763035	0.000669709	FAM189A2
ENSG00000042286	-1.449843816	0.000675945	AIFM2
ENSG00000272468	1.813988404	0.000679363	AL021807.1
ENSG00000135116	1.768964501	0.00068974	HRK
ENSG00000106538	-1.492428205	0.000714476	RARRES2
ENSG00000170439	-1.702475488	0.000729229	METTL7B
ENSG00000183778	-1.461145399	0.000734795	B3GALT5
ENSG00000064932	1.414844775	0.000737444	SBNO2
ENSG00000261613	3.237604351	0.000739506	AC093525.6
ENSG00000272800	-3.385963936	0.000739506	AC021851.1
ENSG00000162006	2.161411814	0.000759269	MSLNL
ENSG00000183779	-1.526821989	0.000761088	ZNF703
ENSG00000229821	-2.132730905	0.000812684	AC103925.1
ENSG00000187889	-2.132730905	0.000812684	FYB2
ENSG00000266970	-1.958963455	0.000817574	AC061992.1
ENSG00000080573	1.524797627	0.000826012	COL5A3
ENSG00000253816	2.863218976	0.000829981	AC138866.1
ENSG00000259511	2.863218976	0.000829981	UBE2Q2L
ENSG00000156886	-1.482799506	0.000835051	ITGAD
ENSG00000197582	-1.587020434	0.000836987	GPX1P1
ENSG00000121858	-1.449138134	0.000847242	TNFSF10
ENSG00000285517	1.491930295	0.000848762	LINC00941
ENSG00000119922	-1.496923202	0.000853262	IFIT2
ENSG00000169918	1.741518108	0.000856071	OTUD7A
ENSG00000183153	1.702051027	0.000856742	GJD3
ENSG00000148704	6.749064406	0.00085757	VAX1
ENSG00000259462	6.749064406	0.00085757	CPEB1-AS1
ENSG00000272861	6.749064406	0.00085757	AC012360.2
ENSG00000241983	6.749064406	0.00085757	RN7SL566P
ENSG00000224609	-2.216276991	0.000860935	HSD52
ENSG00000271425	1.846223613	0.000871067	NBPF10

ENSG00000080561	1.492905949	0.000871315	MID2
ENSG00000139364	2.374191952	0.000877744	TMEM132B
ENSG00000066382	-2.536416287	0.000877744	MPPED2
ENSG00000130508	-1.404865793	0.000882006	PXDN
ENSG00000186994	-2.651813556	0.000883969	KANK3
ENSG00000244257	-2.651813556	0.000883969	PKD1P1
ENSG00000257964	-3.979867995	0.000884859	AC020612.3
ENSG00000120471	-3.979867995	0.000884859	TP53AIP1
ENSG00000068438	1.396793157	0.000894765	FTSJ1
ENSG00000196139	-1.399662827	0.000919183	AKR1C3
ENSG00000167123	-1.426960704	0.000930589	CERCAM
ENSG00000242071	-1.586909606	0.0009307	RPL7AP6
ENSG00000269427	1.866485045	0.000951973	AC024075.3
ENSG00000205336	1.381588735	0.000960272	ADGRG1
ENSG00000118785	-1.378130795	0.000989559	SPP1
ENSG00000074047	1.437042671	0.000996115	GLI2
ENSG00000103313	-1.835951422	0.001005496	MEFV
ENSG00000113657	1.461600874	0.001025217	DPYSL3
ENSG00000054277	-1.709464256	0.001043731	OPN3
ENSG00000188488	1.914741491	0.001049844	SERPINA5
ENSG00000132837	1.914741491	0.001049844	DMGDH
ENSG00000175556	1.450666011	0.001065526	LONRF3
ENSG00000091129	1.392569158	0.001070503	NRCAM
ENSG00000176473	-1.416180877	0.001074822	WDR25
ENSG00000213366	2.053941701	0.001082741	GSTM2
ENSG00000188629	3.17089791	0.001084588	ZNF177
ENSG00000255571	-3.312410996	0.001084588	MIR9-3HG
ENSG00000155269	-3.312410996	0.001084588	GPR78
ENSG00000146966	-2.173360261	0.001111067	DENND2A
ENSG00000115112	-1.426264135	0.001118279	TFCP2L1
ENSG00000161267	-1.476560241	0.001124841	BDH1
ENSG00000142910	1.370870215	0.001126726	TINAGL1
ENSG00000249835	-1.69281816	0.001127459	VCAN-AS1
ENSG00000246228	-1.653570927	0.001136297	CASC8
ENSG00000283199	2.102675648	0.001142968	C13orf46
ENSG00000265018	-2.967682147	0.001173602	AGAP12P
ENSG00000163633	2.804639492	0.001173602	C4orf36
ENSG00000225447	-2.967682147	0.001173602	RPS15AP10
ENSG00000198753	1.699343935	0.001185854	PLXNB3
ENSG00000274341	-2.75135224	0.001197455	AC005899.6
ENSG00000166523	-2.75135224	0.001197455	CLEC4E
ENSG00000174951	-2.75135224	0.001197455	FUT1
ENSG00000257831	1.805297788	0.001224797	AL136418.1
ENSG00000246090	-1.944657069	0.001239507	AP002026.1
ENSG00000268350	-1.944657069	0.001239507	FAM156A
ENSG00000015413	-1.944657069	0.001239507	DPEP1
ENSG00000172005	-1.396004829	0.001260387	MAL
ENSG00000142494	-2.058954824	0.001279883	SLC47A1
ENSG00000228253	-1.396094015	0.001301977	MT-ATP8
ENSG00000240065	-1.391556962	0.001350347	PSMB9
ENSG00000134256	-2.090621062	0.001356148	CD101
ENSG00000171533	1.452090742	0.001362219	MAP6
ENSG00000278936	3.789554151	0.001369576	AC244517.4
ENSG00000261140	3.789554151	0.001369576	AC093525.4
ENSG00000272366	3.789554151	0.001369576	AL158211.3
ENSG00000033122	-3.88746625	0.001369576	LRRC7
ENSG00000280387	-3.88746625	0.001369576	AL109806.1
ENSG00000261604	-3.88746625	0.001369576	AC114947.2
ENSG00000110195	1.373315322	0.001383783	FOLR1
ENSG00000265366	1.949902144	0.001400255	GLUD1P2
ENSG00000112183	2.072385781	0.00140429	RBM24

ENSG00000285820	2.072385781	0.00140429	AL157829.1
ENSG00000150627	-1.795908816	0.00142216	WDR17
ENSG00000231226	6.528905641	0.00143701	TRIM31-AS1
ENSG00000235934	6.528905641	0.00143701	AC007405.2
ENSG00000260911	6.64318055	0.00143701	AC135050.3
ENSG00000228663	6.528905641	0.00143701	PSMD10P1
ENSG00000233266	6.528905641	0.00143701	HMGB1P31
ENSG00000273013	6.64318055	0.00143701	AC117490.2
ENSG00000253302	6.64318055	0.00143701	STAU2-AS1
ENSG00000273186	-6.674677294	0.00143701	AL359091.5
ENSG00000149403	6.528905641	0.00143701	GRIK4
ENSG00000275645	6.64318055	0.00143701	AC068338.3
ENSG00000267651	6.64318055	0.00143701	AC015961.1
ENSG00000229955	6.528905641	0.00143701	Z98749.1
ENSG00000135773	6.528905641	0.00143701	CAPN9
ENSG00000274015	6.528905641	0.00143701	AL136038.5
ENSG00000230140	6.64318055	0.00143701	AC016738.2
ENSG00000273113	-6.674677294	0.00143701	AC133528.1
ENSG00000204659	6.528905641	0.00143701	CBY3
ENSG00000236324	6.64318055	0.00143701	AL035634.1
ENSG00000265724	6.528905641	0.00143701	MIR4284
ENSG00000265727	6.528905641	0.00143701	RN7SL648P
ENSG00000277738	-6.674677294	0.00143701	AC126175.1
ENSG00000143195	-6.674677294	0.00143701	ILDR2
ENSG00000275227	6.528905641	0.00143701	RF02271
ENSG00000188306	6.64318055	0.00143701	LRRIQ4
ENSG00000234197	6.64318055	0.00143701	ETV5-AS1
ENSG00000274114	6.528905641	0.00143701	ALOX15P1
ENSG00000215930	6.528905641	0.00143701	MIR942
ENSG00000226268	6.528905641	0.00143701	AC135977.1
ENSG00000256151	-6.674677294	0.00143701	ADGRD1-AS1
ENSG00000225530	-6.674677294	0.00143701	SP3P
ENSG00000137766	-6.674677294	0.00143701	UNC13C
ENSG00000278384	-6.674677294	0.00143701	AL354822.1
ENSG00000213906	1.543659609	0.001446533	LTB4R2
ENSG00000168003	1.328885743	0.001463397	SLC3A2
ENSG00000261485	1.868297335	0.0014637	PAN3-AS1
ENSG00000182010	-1.444245486	0.001478176	RTKN2
ENSG00000205832	1.957637157	0.001496038	C16orf96
ENSG00000136573	2.24541463	0.001516645	BLK
ENSG00000270419	-2.443662062	0.001516645	CAHM
ENSG00000205583	-1.408530975	0.00152287	STAG3L1
ENSG00000279342	-1.770972345	0.001550451	AP000866.6
ENSG00000260528	1.66334844	0.001551286	FAM157C
ENSG00000056998	-1.59934473	0.001575555	GYG2
ENSG00000100055	-2.549155354	0.001591547	CYTH4
ENSG00000260193	2.361456888	0.001591547	AL138781.1
ENSG00000213711	-3.234905701	0.001597933	PHBP7
ENSG00000278903	3.100956979	0.001597933	CU633906.2
ENSG00000271032	-3.234905701	0.001597933	AC020907.4
ENSG00000203650	3.100956979	0.001597933	LINC01285
ENSG00000261889	-3.234905701	0.001597933	AC108134.2
ENSG00000259088	-2.020602013	0.001608974	AL137779.2
ENSG00000105227	1.380918679	0.001625647	PRX
ENSG00000197614	1.328341793	0.00164867	MFAP5
ENSG00000276840	2.518259689	0.001648699	PMS2P10
ENSG00000174004	-2.900936349	0.001665133	NRROS
ENSG00000140470	1.394573236	0.001675159	ADAMTS17
ENSG00000197415	-1.502073693	0.001675862	VEPH1
ENSG00000198814	-1.875092487	0.001677557	GK
ENSG00000167157	-1.455278299	0.001717328	PRRX2

ENSG00000261713	1.609054304	0.001745102	SSTR5-AS1
ENSG00000091137	1.780497109	0.001766661	SLC26A4
ENSG00000213965	1.406518125	0.001779961	NUDT19
ENSG00000277075	1.869394916	0.001860883	HIST1H2AE
ENSG00000187123	-1.839218354	0.001860883	LYPD6
ENSG00000164093	-1.839218354	0.001860883	PITX2
ENSG00000170525	-1.299190374	0.001890565	PFKFB3
ENSG00000225151	1.417685881	0.001918244	GOLGA2P7
ENSG00000099284	-1.322142122	0.001926152	H2AFY2
ENSG00000145335	-1.895658578	0.00195502	SNCA
ENSG00000151150	-1.895658578	0.00195502	ANK3
ENSG00000113249	-1.327907059	0.001966689	HAVCR1
ENSG00000241769	1.532577121	0.001996463	LINC00893
ENSG00000175197	1.320696723	0.001998668	DDIT3
ENSG00000203362	-1.981201715	0.00202491	POLH-AS1
ENSG00000141449	1.336649921	0.002043208	GREB1L
ENSG00000188130	-1.7798656	0.002045087	MAPK12
ENSG00000164683	1.422763632	0.002047139	HEY1
ENSG00000278662	-1.705487042	0.002075695	GOLGA6L10
ENSG00000178773	-1.705487042	0.002075695	CPNE7
ENSG00000069812	1.452808888	0.00209876	HES2
ENSG00000186998	-1.90833768	0.002125384	EMID1
ENSG00000101955	-1.484291866	0.002126853	SRPX
ENSG00000274272	3.702782619	0.002133716	AC069281.2
ENSG00000139626	3.702782619	0.002133716	ITGB7
ENSG00000248099	3.702782619	0.002133716	INSL3
ENSG00000168126	3.702782619	0.002133716	OR2W6P
ENSG00000234949	3.702782619	0.002133716	AC104667.2
ENSG00000101230	3.702782619	0.002133716	ISM1
ENSG00000275179	3.702782619	0.002133716	AL118508.2
ENSG00000244363	-3.788738904	0.002133716	RPL7P23
ENSG00000248608	-3.788738904	0.002133716	AC133963.1
ENSG00000283016	2.31106316	0.002142368	AL139353.3
ENSG00000272078	-2.494950271	0.002142368	AL139423.1
ENSG00000221869	-1.292835621	0.002166241	CEBPD
ENSG00000178977	2.235679518	0.002205393	LINC00324
ENSG00000105696	2.235679518	0.002205393	TMEM59L
ENSG00000154258	2.235679518	0.002205393	ABCA9
ENSG00000241388	-2.006027479	0.002205393	HNF1A-AS1
ENSG00000228952	1.654767371	0.002217391	LINC02041
ENSG00000183850	-1.449193839	0.002219924	ZNF730
ENSG00000187391	1.720572673	0.002231945	MAGI2
ENSG00000135925	1.289364712	0.00223331	WNT10A
ENSG00000235974	1.329332241	0.002249001	VN2R19P
ENSG00000164344	2.461966257	0.002275868	KLKB1
ENSG00000227440	-2.631650341	0.002275868	ATP5MC1P4
ENSG00000099994	-1.576055448	0.002312478	SUSD2
ENSG00000178401	-1.352196121	0.002321619	DNAJC22
ENSG00000101928	1.343844251	0.002326143	MOSPD1
ENSG00000145358	-1.503705034	0.002328224	DDIT4L
ENSG00000179869	3.027451832	0.002364823	ABCA13
ENSG00000224848	3.027451832	0.002364823	AL589843.1
ENSG00000236830	3.027451832	0.002364823	CBR3-AS1
ENSG00000119915	-3.152999051	0.002364823	ELOVL3
ENSG00000266850	3.027451832	0.002364823	AC090912.2
ENSG00000175206	-3.152999051	0.002364823	NPPA
ENSG00000266910	-3.152999051	0.002364823	AC008507.1
ENSG00000185689	2.679822458	0.002370371	C6orf201
ENSG00000086159	-2.83095215	0.002370371	AQP6
ENSG00000267421	2.679822458	0.002370371	AC005498.2
ENSG00000207973	-2.83095215	0.002370371	MIR589

ENSG00000250510	-2.83095215	0.002370371	GPR162
ENSG00000234705	-2.83095215	0.002370371	HMGA1P4
ENSG00000175920	1.298425628	0.002385649	DOK7
ENSG00000163734	1.512591806	0.002414359	CXCL3
ENSG00000177822	-1.752446821	0.002419176	AC098864.1
ENSG00000228812	6.404794377	0.002434933	LAMA5-AS1
ENSG00000272509	-6.55042966	0.002434933	AC087752.4
ENSG00000224903	-6.55042966	0.002434933	AC005534.1
ENSG00000203706	-6.55042966	0.002434933	SERTAD4-AS1
ENSG00000237054	-6.55042966	0.002434933	PRMT5-AS1
ENSG00000267016	6.404794377	0.002434933	AC111170.1
ENSG00000279459	6.404794377	0.002434933	AP001271.2
ENSG00000244151	-6.55042966	0.002434933	AC010973.2
ENSG00000147059	6.404794377	0.002434933	SPIN2A
ENSG00000257075	6.404794377	0.002434933	RPEP6
ENSG00000241369	-6.55042966	0.002434933	LINC01192
ENSG00000213700	-6.55042966	0.002434933	RPL17P50
ENSG00000278598	6.404794377	0.002434933	MIR6775
ENSG00000163606	6.404794377	0.002434933	CD200R1
ENSG00000253030	6.404794377	0.002434933	MIR2116
ENSG00000266718	6.404794377	0.002434933	AC079336.5
ENSG00000242252	6.404794377	0.002434933	BGLAP
ENSG00000248664	-6.55042966	0.002434933	AC010273.1
ENSG00000225192	-6.55042966	0.002434933	ZNF33BP1
ENSG00000274367	-6.55042966	0.002434933	AC004233.4
ENSG00000278770	-6.55042966	0.002434933	RF00017
ENSG00000272004	6.404794377	0.002434933	FO704657.1
ENSG00000240241	-6.55042966	0.002434933	AC108752.1
ENSG00000248839	-6.55042966	0.002434933	AC021660.2
ENSG00000271795	-6.55042966	0.002434933	AC011337.1
ENSG00000271631	6.404794377	0.002434933	AL139041.1
ENSG00000165188	-6.55042966	0.002434933	RNF183
ENSG00000280339	6.404794377	0.002434933	AP001528.3
ENSG00000231738	-6.55042966	0.002434933	TSPAN19
ENSG00000252806	-6.55042966	0.002434933	RNA5SP420
ENSG00000264970	-6.55042966	0.002434933	AC131274.3
ENSG00000126878	-1.316646677	0.002526717	AIF1L
ENSG00000106113	2.121729771	0.002551039	CRHR2
ENSG00000166341	1.294306336	0.002552002	DCHS1
ENSG00000156427	1.275327842	0.002560999	FGF18
ENSG00000137486	-1.265502371	0.002565342	ARRB1
ENSG00000196562	1.269289161	0.002591114	SULF2
ENSG00000169994	1.457341068	0.002614377	MYO7B
ENSG00000274791	2.152698421	0.002639002	F8A2
ENSG00000123999	2.152698421	0.002639002	INHA
ENSG00000062038	2.152698421	0.002639002	CDH3
ENSG00000266010	2.152698421	0.002639002	GATA6-AS1
ENSG00000253404	-2.344532201	0.002639002	AC034243.1
ENSG00000132429	-2.344532201	0.002639002	POPDC3
ENSG00000174059	1.87212986	0.002641227	CD34
ENSG00000228060	1.87212986	0.002641227	AL365277.1
ENSG00000120738	1.311575228	0.002645158	EGR1
ENSG00000267904	1.575776377	0.002676994	AC024075.1
ENSG00000105549	1.609557653	0.002684397	THEG
ENSG00000273769	1.418484227	0.002721298	LCA10
ENSG00000138316	1.58545661	0.002726232	ADAMTS14
ENSG00000279821	1.664719671	0.002776837	AC145098.2
ENSG00000139278	-1.55179429	0.002778801	GLIPR1
ENSG00000198515	2.196292936	0.002817474	CNGA1
ENSG00000155962	1.770667571	0.002863026	CLIC2
ENSG00000275180	-1.438304602	0.002870513	AC048341.2

ENSG00000142627	1.245130922	0.002870745	EPHA2
ENSG00000248319	-1.42250447	0.002879452	LINC02275
ENSG00000139508	-1.472922616	0.002888858	SLC46A3
ENSG00000235523	2.258845255	0.00288962	AL135924.2
ENSG00000238076	1.616865047	0.002893945	MRPL48P1
ENSG00000253537	1.394536698	0.002926697	PCDHGA7
ENSG00000230535	-1.28951034	0.002963331	BASP1P1
ENSG00000151474	-1.496058204	0.002967311	FRMD4A
ENSG00000128268	-1.278091387	0.002995724	MGAT3
ENSG00000237167	1.521655335	0.003034831	AC128709.3
ENSG00000273619	1.488468905	0.003077813	AL121832.2
ENSG00000130821	1.271504409	0.003083664	SLC6A8
ENSG00000197043	-1.243648503	0.00310774	ANXA6
ENSG00000227063	-1.479999746	0.003113205	RPL41P1
ENSG00000163485	-2.036373391	0.003128224	ADORA1
ENSG00000151704	-2.225692445	0.003128224	KCNJ1
ENSG00000150625	-2.567856448	0.003149424	GPM6A
ENSG00000214725	-2.567856448	0.003149424	CDIPTOSP
ENSG00000153029	-1.396962671	0.003172486	MR1
ENSG00000005187	-1.360924439	0.003180268	ACSM3
ENSG00000166819	-1.899018249	0.003217043	PLIN1
ENSG00000151572	-1.506354021	0.003233442	ANO4
ENSG00000155368	-1.233195022	0.003245253	DBI
ENSG00000272398	-1.225672861	0.003290156	CD24
ENSG00000223722	1.689057442	0.00330135	AC023157.1
ENSG00000074527	1.225661923	0.003328764	NTN4
ENSG00000167767	1.22718481	0.003335562	KRT80
ENSG00000260466	3.610456297	0.003346055	AC126696.2
ENSG00000285424	3.610456297	0.003346055	AL162718.1
ENSG00000274372	3.610456297	0.003346055	AC239803.3
ENSG00000244693	-3.682755825	0.003346055	CTAGE8
ENSG00000261342	3.610456297	0.003346055	AC006538.1
ENSG00000198691	3.610456297	0.003346055	ABCA4
ENSG00000260498	3.610456297	0.003346055	AC126696.3
ENSG00000153253	-3.682755825	0.003346055	SCN3A
ENSG00000282961	-3.682755825	0.003346055	PRNCR1
ENSG00000184937	-3.682755825	0.003346055	WT1
ENSG00000092200	-3.682755825	0.003346055	RPGRIP1
ENSG00000243323	-3.682755825	0.003346055	PTPRVP
ENSG00000279022	-3.682755825	0.003346055	AL359715.4
ENSG00000245685	-2.75739921	0.003385132	FRG1-DT
ENSG00000268883	2.613116017	0.003385132	PNMA6B
ENSG00000283265	1.303404784	0.003397185	AL356234.3
ENSG00000125775	1.447424294	0.003403601	SDCBP2
ENSG00000250696	1.313773521	0.003447655	AC111000.4
ENSG00000007314	1.786808549	0.003476403	SCN4A
ENSG00000265943	2.10400571	0.003489554	AC090912.1
ENSG00000233776	2.10400571	0.003489554	LINC01251
ENSG00000140678	-1.466196654	0.003497406	ITGAX
ENSG00000196872	1.335681115	0.003508255	KIAA1211L
ENSG00000271897	2.949999604	0.003515158	AL357518.1
ENSG00000171346	2.949999604	0.003515158	KRT15
ENSG00000162620	-3.066160903	0.003515158	LRRIQ3
ENSG00000129437	2.949999604	0.003515158	KLK14
ENSG00000254154	-3.066160903	0.003515158	CRYZL2P-SEC16B
ENSG00000285884	2.949999604	0.003515158	AL022345.4
ENSG00000168124	-3.066160903	0.003515158	OR1F1
ENSG00000224805	-3.066160903	0.003515158	LINC00853
ENSG00000074181	-3.066160903	0.003515158	NOTCH3
ENSG00000172346	-3.066160903	0.003515158	CSDC2
ENSG00000224858	1.374631313	0.00352744	RPL29P11

ENSG00000279339	1.873863613	0.00355334	AC100788.2
ENSG00000280106	2.155800821	0.00360343	AC008555.8
ENSG00000074410	1.213483299	0.003628457	CA12
ENSG00000120129	1.216777349	0.003645612	DUSP1
ENSG00000168916	-1.345673693	0.003661372	ZNF608
ENSG00000242960	-1.631514823	0.003723589	FTH1P23
ENSG00000227507	1.293730637	0.003734773	LTB
ENSG00000108375	-1.601435356	0.003780774	RNF43
ENSG00000215105	1.251038732	0.003795163	TTC3P1
ENSG00000205485	1.424264412	0.003813434	AC004980.1
ENSG00000133874	-1.451714351	0.003837969	RNF122
ENSG00000121005	-1.278629427	0.00387615	CRISPLD1
ENSG00000092295	1.405082661	0.003893839	TGM1
ENSG00000260186	2.20466613	0.003904919	LINC02137
ENSG00000183479	2.20466613	0.003904919	TREX2
ENSG00000169860	-1.585848464	0.003962214	P2RY1
ENSG00000108771	-1.307355668	0.003971283	DHX58
ENSG00000100031	-1.473975702	0.003976089	GGT1
ENSG00000224195	-1.799041737	0.003980794	AC022400.1
ENSG00000198105	-1.230718478	0.003993357	ZNF248
ENSG00000179299	-1.695994633	0.004015113	NSUN7
ENSG00000107954	1.233770565	0.004025815	NEURL1
ENSG00000111644	-1.856101519	0.004060649	ACRBP
ENSG00000226644	2.046042707	0.004060649	AL121899.1
ENSG00000185275	-1.987659714	0.00406587	CD24P4
ENSG00000063438	1.317681412	0.004091456	AHRR
ENSG00000182621	-1.626322699	0.004091611	PLCB1
ENSG00000257815	1.563535961	0.004127818	LINC01481
ENSG00000173918	-1.328030789	0.004141343	C1QTNF1
ENSG00000236618	-6.414464529	0.004174171	PITPNA-AS1
ENSG00000203721	6.268992554	0.004174171	LINC00862
ENSG00000268738	6.268992554	0.004174171	HSFX2
ENSG00000224001	6.268992554	0.004174171	AL360081.1
ENSG00000260213	-6.414464529	0.004174171	AC092718.2
ENSG00000220614	6.268992554	0.004174171	AL583834.1
ENSG00000258844	6.268992554	0.004174171	AL162511.1
ENSG00000169856	-6.414464529	0.004174171	ONECUT1
ENSG00000280295	6.268992554	0.004174171	AC099811.6
ENSG00000167807	6.268992554	0.004174171	AC011511.1
ENSG00000174125	-6.414464529	0.004174171	TLR1
ENSG00000232814	-6.414464529	0.004174171	COL4A2-AS1
ENSG00000260910	6.268992554	0.004174171	LINC00565
ENSG00000276096	6.268992554	0.004174171	RF00017
ENSG00000252182	-6.414464529	0.004174171	RNA5SP413
ENSG00000267424	-6.414464529	0.004174171	AC020934.1
ENSG00000105509	6.268992554	0.004174171	HAS1
ENSG00000231871	-6.414464529	0.004174171	IPO9-AS1
ENSG00000234424	-6.414464529	0.004174171	AL353743.4
ENSG00000173947	-6.414464529	0.004174171	PIFO
ENSG00000230426	-6.414464529	0.004174171	ERVMER61-1
ENSG00000234076	-6.414464529	0.004174171	TPRG1-AS1
ENSG00000251821	-6.414464529	0.004174171	RNU6-583P
ENSG00000164418	-6.414464529	0.004174171	GRIK2
ENSG00000268902	-6.414464529	0.004174171	CSAG2
ENSG00000165092	-6.414464529	0.004174171	ALDH1A1
ENSG00000199325	6.268992554	0.004174171	RNU4-39P
ENSG00000198488	-6.414464529	0.004174171	B3GNT6
ENSG00000274156	-6.414464529	0.004174171	AC008121.2
ENSG00000273523	6.268992554	0.004174171	AL139082.1
ENSG00000285664	6.268992554	0.004174171	AL139317.5
ENSG00000100652	-6.414464529	0.004174171	SLC10A1

ENSG00000261147	-6.414464529	0.004174171	AC091167.2
ENSG00000280160	-6.414464529	0.004174171	AC135050.7
ENSG00000153976	-6.414464529	0.004174171	HS3ST3A1
ENSG00000125409	6.268992554	0.004174171	TEKT3
ENSG00000108551	6.268992554	0.004174171	RASD1
ENSG00000273091	-6.414464529	0.004174171	AP000255.1
ENSG00000272657	-6.414464529	0.004174171	AP000317.2
ENSG00000196890	1.75773936	0.004176503	HIST3H2BB
ENSG00000212123	1.306019307	0.004185493	PRR22
ENSG00000169020	-1.30110289	0.00419005	ATP5ME
ENSG00000078018	1.272745622	0.004215507	MAP2
ENSG00000167995	1.286819712	0.004218439	BEST1
ENSG00000220412	1.389771728	0.004258494	AL356234.1
ENSG00000162407	-1.234297636	0.004335747	PLPP3
ENSG00000146122	-1.758542909	0.004341912	DAAM2
ENSG00000281501	-1.758542909	0.004341912	SEPSECS-AS1
ENSG00000279569	2.342327678	0.004368548	AC020763.4
ENSG00000181908	2.342327678	0.004368548	AP003774.1
ENSG00000233013	-1.435379964	0.004370755	FAM157B
ENSG00000105963	1.229314749	0.004397684	ADAP1
ENSG00000099875	1.184972885	0.004469724	MKNK2
ENSG00000138623	1.246959659	0.004474369	SEMA7A
ENSG00000133101	1.323123517	0.004491385	CCNA1
ENSG00000170915	-1.201336603	0.004500423	PAQR8
ENSG00000180044	1.569514438	0.004529785	C3orf80
ENSG00000204257	-1.449763578	0.004547952	HLA-DMA
ENSG00000130635	1.201971186	0.004572809	COL5A1
ENSG00000234353	2.071238857	0.004613369	AP000346.2
ENSG00000226180	2.071238857	0.004613369	AC010536.1
ENSG00000248635	2.071238857	0.004613369	AC147055.1
ENSG00000272449	2.053611982	0.004621058	AL139246.5
ENSG00000164120	-2.238085101	0.004621058	HPGD
ENSG00000125731	1.214070267	0.004622996	SH2D3A
ENSG00000197444	-1.213729184	0.004710176	OGDHL
ENSG00000238042	1.542514663	0.004739774	LINC02257
ENSG00000280109	1.465747876	0.004746963	PLAC4
ENSG00000128342	1.178963926	0.004759401	LIF
ENSG00000285867	1.390875286	0.004829949	BX470102.2
ENSG00000185345	-1.35910245	0.004829949	PRKN
ENSG00000134955	1.325877659	0.004831613	SLC37A2
ENSG00000273102	2.543175086	0.004849114	AP000569.1
ENSG00000277287	2.543175086	0.004849114	AL109976.1
ENSG00000203327	-2.679893915	0.004849114	AC012358.1
ENSG00000206612	-2.679893915	0.004849114	SNORA2A
ENSG00000233672	-2.679893915	0.004849114	RNASEH2B-AS1
ENSG00000250282	-2.679893915	0.004849114	AC002401.2
ENSG00000151640	1.233493395	0.004856231	DPYSL4
ENSG00000055118	-1.48533612	0.004879682	KCNH2
ENSG00000133134	1.376753586	0.004904444	BEX2
ENSG00000106366	1.270249562	0.004913066	SERPINE1
ENSG00000122733	1.875937313	0.004913674	PHF24
ENSG00000261732	-1.760688926	0.004913674	AL031708.1
ENSG00000143110	-1.760688926	0.004913674	C1orf162
ENSG00000121797	1.22298682	0.004951464	CCRL2
ENSG00000004838	-1.581863357	0.004998675	ZMYND10
ENSG00000025708	-1.347936063	0.005132638	TYMP
ENSG00000182054	-1.180140431	0.00517822	IDH2
ENSG00000104892	1.247771101	0.005184251	KLC3
ENSG00000162645	-1.564808382	0.00522492	GBP2
ENSG00000162772	1.319650164	0.005229099	ATF3
ENSG00000183049	-1.267333341	0.005239493	CAMK1D

ENSG00000233602	2.868152218	0.005247305	ERI3-IT1
ENSG00000249958	2.868152218	0.005247305	CCT7P2
ENSG00000176387	-2.973759157	0.005247305	HSD11B2
ENSG00000254389	-2.973759157	0.005247305	RHPN1-AS1
ENSG00000228828	-2.973759157	0.005247305	TLK2P2
ENSG00000272211	-2.973759157	0.005247305	AC114760.2
ENSG00000269421	-2.973759157	0.005247305	ZNF92P3
ENSG00000232470	-2.973759157	0.005247305	AL136368.1
ENSG00000101307	-2.973759157	0.005247305	SIRPB1
ENSG00000175707	1.450003166	0.005278575	KDF1
ENSG00000253908	3.511815055	0.005281544	AC104115.2
ENSG00000131746	3.511815055	0.005281544	TNS4
ENSG00000269694	3.511815055	0.005281544	AC005197.1
ENSG00000226310	3.511815055	0.005281544	AL022157.1
ENSG00000258534	3.511815055	0.005281544	AL132712.1
ENSG00000255020	3.511815055	0.005281544	AF131216.3
ENSG00000259744	3.511815055	0.005281544	AC009269.4
ENSG00000225676	2.148372698	0.005286278	AC002378.1
ENSG00000166343	-2.318926915	0.005286278	MSS51
ENSG00000249700	-1.93724353	0.005290723	SRD5A3-AS1
ENSG00000159208	-1.593147207	0.005296084	CIART
ENSG00000183963	-1.227120856	0.005313077	SMTN
ENSG00000157240	-1.176570193	0.005324757	FZD1
ENSG00000134986	-1.208887107	0.005333578	NREP
ENSG00000130766	1.1927782	0.005376632	SESN2
ENSG00000121316	-1.437168962	0.005394739	PLBD1
ENSG00000175130	-1.191350002	0.005407897	MARCKSL1
ENSG00000170485	-1.261615578	0.005422401	NPAS2
ENSG00000237886	-1.464906394	0.005520559	NALT1
ENSG00000070669	1.583355723	0.005549857	ASNS
ENSG00000166979	-1.763216426	0.00561466	EVA1C
ENSG00000092529	-1.763216426	0.00561466	CAPN3
ENSG00000188368	1.496790163	0.005654816	PRR19
ENSG00000235720	1.749615979	0.005682197	GABPAP
ENSG00000063587	1.175983923	0.005719657	ZNF275
ENSG00000186765	1.553054462	0.005793699	FSCN2
ENSG00000146555	-1.869040609	0.005911861	SDK1
ENSG00000170423	2.027023499	0.005911861	KRT78
ENSG00000272106	1.276220725	0.005932958	AL691432.2
ENSG00000128573	1.342406591	0.006063918	FOXP2
ENSG00000136866	-1.721288629	0.006068669	ZFP37
ENSG00000189014	-2.431126451	0.006072876	SHLD2P3
ENSG00000230082	-2.431126451	0.006072876	PRRT3-AS1
ENSG00000277156	-2.431126451	0.006072876	AL589743.5
ENSG00000116774	-2.431126451	0.006072876	OLFML3
ENSG00000204962	-2.431126451	0.006072876	PCDHA8
ENSG00000146904	2.001394077	0.006127713	EPHA1
ENSG00000154479	2.001394077	0.006127713	CCDC173
ENSG00000225328	-2.181763644	0.006127713	LINC01594
ENSG00000119986	1.166334865	0.006226431	AVPI1
ENSG00000163520	-1.242246989	0.006258551	FBLN2
ENSG00000184678	1.221130848	0.006272242	HIST2H2BE
ENSG00000106078	1.18002817	0.006361481	COBL
ENSG00000177599	1.65175628	0.00637015	ZNF491
ENSG00000166444	-1.163113814	0.006412738	ST5
ENSG00000138587	-1.222262302	0.006423833	MNS1
ENSG00000120662	-1.26725587	0.006454302	MTRF1
ENSG00000142530	1.475768865	0.006469214	FAM71E1
ENSG00000250920	1.138463043	0.006476884	AC105460.1
ENSG00000183625	-1.811868874	0.00648527	CCR3
ENSG00000230359	-1.684766828	0.006493106	TPI1P2

ENSG00000174529	-1.684766828	0.006493106	TMEM81
ENSG00000227051	-1.217347846	0.006560123	C14orf132
ENSG00000102287	-1.2612326	0.006684117	GABRE
ENSG00000103740	-1.606945237	0.006686163	ACSBG1
ENSG00000204524	1.275064752	0.006698565	ZNF805
ENSG00000169129	1.193608578	0.006704305	AFAP1L2
ENSG00000157570	-1.175388604	0.00670647	TSPAN18
ENSG00000130943	1.529645497	0.006716517	PKDREJ
ENSG00000219891	1.195604125	0.006752884	ZSCAN12P1
ENSG00000132274	-1.250252853	0.00687511	TRIM22
ENSG00000137968	-1.432383316	0.006876664	SLC44A5
ENSG00000273568	1.717289943	0.006889608	AC131009.3
ENSG00000138028	-2.074320566	0.006891763	CGREF1
ENSG00000226874	-1.885001512	0.006891763	AC005154.1
ENSG00000205334	-1.885001512	0.006891763	LINC01460
ENSG00000115738	-1.173339467	0.006941374	ID2
ENSG00000110203	1.502536713	0.006955656	FOLR3
ENSG00000228705	2.46966994	0.006966163	LINC00659
ENSG00000268751	2.46966994	0.006966163	SCGB1B2P
ENSG00000268756	2.46966994	0.006966163	AC104534.1
ENSG00000243646	2.46966994	0.006966163	IL10RB
ENSG00000111275	-2.597987265	0.006966163	ALDH2
ENSG00000268812	1.390075831	0.006969265	AC004264.1
ENSG00000135636	1.573546623	0.006984897	DYSF
ENSG00000109101	1.600447608	0.006984897	FOXN1
ENSG00000104213	-1.585070587	0.006984897	PDGFRL
ENSG00000164746	1.600447608	0.006984897	C7orf57
ENSG00000118777	1.878461813	0.00701327	ABCG2
ENSG00000067064	-1.131631289	0.007059673	IDI1
ENSG00000260285	-1.384757238	0.007106768	AL133367.1
ENSG00000185739	-1.372037182	0.007107623	SRL
ENSG00000144810	-2.255133023	0.007167784	COL8A1
ENSG00000254701	-2.255133023	0.007167784	AC138866.2
ENSG00000109452	-1.501050443	0.007183936	INPP4B
ENSG00000115963	1.130713118	0.007196944	RND3
ENSG00000181773	1.235698144	0.007199964	GPR3
ENSG00000185567	1.11632872	0.007214742	AHNAK2
ENSG00000269318	6.119066595	0.007243414	AC007292.2
ENSG00000253508	-6.264339468	0.007243414	AC004080.2
ENSG00000249679	6.119066595	0.007243414	AC106897.1
ENSG00000107859	6.119066595	0.007243414	PITX3
ENSG00000171847	-6.264339468	0.007243414	FAM90A1
ENSG00000280071	-6.264339468	0.007243414	GATD3B
ENSG00000250280	6.119066595	0.007243414	AC026124.1
ENSG00000272797	6.119066595	0.007243414	AC092954.1
ENSG00000277968	-6.264339468	0.007243414	RF02038
ENSG00000232234	-6.264339468	0.007243414	AL355499.1
ENSG00000232149	6.119066595	0.007243414	FERP1
ENSG00000258935	6.119066595	0.007243414	AL096869.1
ENSG00000178081	-6.264339468	0.007243414	ULK4P3
ENSG00000266373	-6.264339468	0.007243414	AP002472.1
ENSG00000267323	6.119066595	0.007243414	SLC25A1P5
ENSG00000269403	6.119066595	0.007243414	AC008750.8
ENSG00000230002	-6.264339468	0.007243414	ALMS1-IT1
ENSG00000223576	-6.264339468	0.007243414	AL355001.1
ENSG00000187556	-6.264339468	0.007243414	NANOS3
ENSG00000285836	-6.264339468	0.007243414	AC092896.2
ENSG00000279891	6.119066595	0.007243414	FLJ42393
ENSG00000270480	-6.264339468	0.007243414	AC073413.1
ENSG00000188883	6.119066595	0.007243414	KLRG2
ENSG00000260220	6.119066595	0.007243414	CCDC187

ENSG00000235910	6.119066595	0.007243414	APOA1-AS
ENSG00000122862	-6.264339468	0.007243414	SRGN
ENSG00000257647	-6.264339468	0.007243414	AC124312.2
ENSG00000241157	-6.264339468	0.007243414	AC104763.1
ENSG00000265142	6.119066595	0.007243414	MIR133A1HG
ENSG00000196628	6.119066595	0.007243414	TCF4
ENSG00000198028	6.119066595	0.007243414	ZNF560
ENSG00000213226	-6.264339468	0.007243414	AC242426.1
ENSG00000285554	6.119066595	0.007243414	AC242988.2
ENSG00000237842	-6.264339468	0.007243414	AL157713.1
ENSG00000175538	-6.264339468	0.007243414	KCNE3
ENSG00000272507	6.119066595	0.007243414	RNU6-88P
ENSG00000207622	6.119066595	0.007243414	MIR619
ENSG00000105679	6.119066595	0.007243414	GAPDHS
ENSG00000275166	6.119066595	0.007243414	MIR6814
ENSG00000162670	-6.264339468	0.007243414	BRINP3
ENSG00000280587	-6.264339468	0.007243414	LINC01348
ENSG00000235770	-6.264339468	0.007243414	LINC00607
ENSG00000235670	6.119066595	0.007243414	RPL21P40
ENSG00000170011	-6.264339468	0.007243414	MYRIP
ENSG00000284731	6.119066595	0.007243414	AC083906.5
ENSG00000241168	-6.264339468	0.007243414	AC128685.1
ENSG00000239247	-6.264339468	0.007243414	RN7SL589P
ENSG00000250215	-6.264339468	0.007243414	CIR1P2
ENSG00000249026	6.119066595	0.007243414	CTNNA1P1
ENSG00000235368	6.119066595	0.007243414	SAPCD2P2
ENSG00000225329	-6.264339468	0.007243414	LHFPL3-AS2
ENSG00000130226	6.119066595	0.007243414	DPP6
ENSG00000196440	6.119066595	0.007243414	ARMCX4
ENSG00000204291	-6.264339468	0.007243414	COL15A1
ENSG00000255071	-6.264339468	0.007243414	SAA2-SAA4
ENSG00000156689	-6.264339468	0.007243414	GLYATL2
ENSG00000229719	6.119066595	0.007243414	MIR194-2HG
ENSG00000275675	6.119066595	0.007243414	AL161931.1
ENSG00000095585	-6.264339468	0.007243414	BLNK
ENSG00000270429	6.119066595	0.007243414	KNOP1P2
ENSG00000111052	-6.264339468	0.007243414	LIN7A
ENSG00000225131	6.119066595	0.007243414	PSME2P2
ENSG00000235420	-6.264339468	0.007243414	RPL29P30
ENSG00000260139	6.119066595	0.007243414	CSPG4P13
ENSG00000204277	-6.264339468	0.007243414	LINC01993
ENSG00000285095	-6.264339468	0.007243414	AC025887.2
ENSG00000268758	6.119066595	0.007243414	ADGRE4P
ENSG00000269139	6.119066595	0.007243414	AC010336.5
ENSG00000270479	-6.264339468	0.007243414	BNIP3P37
ENSG00000225330	-6.264339468	0.007243414	AF064860.1
ENSG00000183092	-1.378327888	0.007262119	BEGAIN
ENSG00000265972	1.11715352	0.007314012	TXNIP
ENSG00000198910	1.113527189	0.007327343	L1CAM
ENSG00000182326	-1.162972146	0.007362002	C1S
ENSG00000163704	1.212755253	0.007384668	PRRT3
ENSG00000181218	1.145377474	0.007406007	HIST3H2A
ENSG00000171451	-1.386249681	0.007430957	DSEL
ENSG00000135828	-1.341529707	0.007499565	RNASEL
ENSG00000154822	-1.301448569	0.00754702	PLCL2
ENSG00000169682	1.981410078	0.007582043	SPNS1
ENSG00000204620	1.981410078	0.007582043	AC115618.1
ENSG00000130294	-1.820326932	0.007582043	KIF1A
ENSG00000145198	1.622089303	0.007606008	VWA5B2
ENSG00000230606	1.622089303	0.007606008	AC092683.1
ENSG00000188243	-1.166290198	0.007731946	COMMD6

ENSG00000197275	-1.209505938	0.007752099	RAD54B
ENSG00000178150	-1.115537717	0.007828301	ZNF114
ENSG00000271347	-2.875031812	0.007864773	AC124312.5
ENSG00000235545	-2.875031812	0.007864773	AC103923.1
ENSG00000226088	2.781380685	0.007864773	AL158839.1
ENSG00000253174	2.781380685	0.007864773	AC009630.2
ENSG00000213221	-2.875031812	0.007864773	DNLZ
ENSG00000213492	2.781380685	0.007864773	NT5C3AP1
ENSG00000248187	2.781380685	0.007864773	AC078850.1
ENSG00000170231	-2.875031812	0.007864773	FABP6
ENSG00000280335	2.781380685	0.007864773	AC016866.3
ENSG00000224072	2.781380685	0.007864773	AL139811.1
ENSG00000114547	2.781380685	0.007864773	ROPN1B
ENSG00000256340	-2.875031812	0.007864773	ABCC6P1
ENSG00000169252	1.180782708	0.007868487	ADRB2
ENSG00000135477	1.582098124	0.007929518	KRT87P
ENSG00000242242	-1.646414017	0.007945536	NECTIN3-AS1
ENSG00000008517	1.135394462	0.008029873	IL32
ENSG00000163661	-2.123153812	0.008135331	PTX3
ENSG00000249328	1.947214952	0.008135331	AC036214.1
ENSG00000231412	1.161285112	0.008160853	AC005392.2
ENSG00000241743	1.881602047	0.008204261	XACT
ENSG00000087494	1.881602047	0.008204261	PTHLH
ENSG00000271576	-1.766237057	0.008204261	AL359504.2
ENSG00000076706	-1.476438299	0.008254561	MCAM
ENSG00000103089	1.117413278	0.008313248	FA2H
ENSG00000171017	1.684222955	0.008356463	LRRC8E
ENSG00000269693	1.684222955	0.008356463	AC010422.6
ENSG00000239620	-1.616242553	0.008356463	PRR20G
ENSG00000230266	3.405931199	0.008390282	XXYLT1-AS2
ENSG00000272072	3.291656289	0.008390282	AC004492.1
ENSG00000078053	3.291656289	0.008390282	AMPH
ENSG00000280054	-3.568365325	0.008390282	AC004241.5
ENSG00000167780	-3.568365325	0.008390282	SOAT2
ENSG00000164530	3.405931199	0.008390282	PI16
ENSG00000277548	3.291656289	0.008390282	AC018926.3
ENSG00000131484	-3.568365325	0.008390282	AC091132.1
ENSG00000196415	3.405931199	0.008390282	PRTN3
ENSG00000254689	-3.568365325	0.008390282	LINC02235
ENSG00000224713	-3.568365325	0.008390282	AC025165.1
ENSG00000233098	-3.568365325	0.008390282	CCDC144NL-AS1
ENSG00000224292	-3.568365325	0.008390282	AF196972.1
ENSG00000134376	-3.568365325	0.008390282	CRB1
ENSG00000241217	-3.568365325	0.008390282	RN7SL809P
ENSG00000156414	3.405931199	0.008390282	TDRD9
ENSG00000186462	-3.568365325	0.008390282	NAP1L2
ENSG00000253217	-3.568365325	0.008390282	AP001574.1
ENSG00000230724	3.291656289	0.008390282	LINC01001
ENSG00000163823	-3.568365325	0.008390282	CCR1
ENSG00000109625	-3.568365325	0.008390282	CPZ
ENSG00000212747	-3.568365325	0.008390282	RTL8B
ENSG00000108242	-3.568365325	0.008390282	CYP2C18
ENSG00000277053	-1.120136729	0.008424998	GTF2IP1
ENSG00000280693	2.211863298	0.008458934	SH3PXD2A-AS1
ENSG00000165168	2.211863298	0.008458934	CYBB
ENSG00000231652	2.211863298	0.008458934	AL590428.1
ENSG00000249738	2.211863298	0.008458934	AC008691.1
ENSG00000269038	-2.357573512	0.008458934	AP001462.1
ENSG00000166823	-2.357573512	0.008458934	MESP1
ENSG00000249715	-2.357573512	0.008458934	FER1L5
ENSG00000254632	-2.357573512	0.008458934	AP003119.1

ENSG00000106976	-1.160257943	0.0085074	DNM1
ENSG00000176244	-1.536695019	0.008515683	ACBD7
ENSG00000152767	-1.100432185	0.008532262	FARP1
ENSG00000173705	1.238798591	0.008590078	SUSD5
ENSG00000113296	1.2927334	0.008594401	THBS4
ENSG00000113205	1.411974973	0.008674877	PCDHB3
ENSG00000108622	1.184773106	0.008715414	ICAM2
ENSG00000140749	-1.676067051	0.008764824	IGSF6
ENSG00000259514	-1.676067051	0.008764824	AC027243.1
ENSG00000146021	1.383412931	0.008838507	KLHL3
ENSG00000196972	1.392623596	0.008869446	SMIM10L2B
ENSG00000165795	-1.388070081	0.008869446	NDRG2
ENSG00000168675	1.145837412	0.008910306	LDLRAD4
ENSG00000271833	-1.83079643	0.00898543	AL445222.1
ENSG00000278948	1.835220851	0.00898543	AL031587.5
ENSG00000253666	-2.020115484	0.00898543	AP000424.1
ENSG00000176046	1.090820642	0.009045852	NUPR1
ENSG00000064763	-1.206280209	0.009080607	FAR2
ENSG00000138172	1.591799436	0.009083827	CALHM2
ENSG00000253958	-1.33666638	0.009128294	CLDN23
ENSG00000183128	1.167907568	0.009197312	CALHM3
ENSG00000117115	-1.303367066	0.009236593	PADI2
ENSG00000120708	-1.098564333	0.009270219	TGFBI
ENSG00000078401	1.111978723	0.009323524	EDN1
ENSG00000144230	1.23342563	0.00944165	GPR17
ENSG00000165757	-1.166215699	0.009442673	JCAD
ENSG00000117013	-1.248351221	0.009456402	KCNQ4
ENSG00000100253	-1.198436488	0.009507041	MIOX
ENSG00000091656	1.126834919	0.00954427	ZFHX4
ENSG00000135454	-1.177167014	0.009571161	B4GALNT1
ENSG00000135638	1.221864264	0.009588275	EMX1
ENSG00000187957	1.518191397	0.009703921	DNER
ENSG00000273024	2.02873412	0.009732694	INTS4P2
ENSG00000226312	2.02873412	0.009732694	CFLAR-AS1
ENSG00000230409	-1.241619449	0.009785777	TCEA1P2
ENSG00000197620	1.167626743	0.009794907	CXorf40A
ENSG00000103534	-1.323269555	0.009997001	TMC5
ENSG00000214558	2.392217711	0.010033723	AL365217.1
ENSG00000179846	-2.511149117	0.010033723	NKPD1
ENSG00000128594	2.392217711	0.010033723	LRRC4
ENSG00000238000	-2.511149117	0.010033723	AC116347.1
ENSG00000180535	2.392217711	0.010033723	BHLHA15
ENSG00000219755	2.392217711	0.010033723	AL137784.1
ENSG00000100146	2.392217711	0.010033723	SOX10
ENSG00000237594	-2.511149117	0.010033723	AP000251.1
ENSG00000235554	-2.511149117	0.010033723	AC005822.1
ENSG00000130173	2.392217711	0.010033723	ANGPTL8
ENSG00000153012	-2.511149117	0.010033723	LGI2
ENSG00000112053	2.392217711	0.010033723	SLC26A8
ENSG00000163376	1.650380246	0.010138421	KBTBD8
ENSG00000165152	-1.578882972	0.010138421	TMEM246
ENSG00000224081	1.176855435	0.010264995	SLC44A3-AS1
ENSG00000204389	-1.072566436	0.010283125	HSPA1A
ENSG00000176933	1.198457206	0.010288986	TOB2P1
ENSG00000237126	-1.71911465	0.010384446	AC073254.1
ENSG00000279227	-1.71911465	0.010384446	AC009303.4
ENSG00000205838	-1.71911465	0.010384446	TTC23L
ENSG00000242375	1.837386688	0.010384446	AL590705.3
ENSG00000270011	1.457049514	0.01047367	ZNF559-ZNF177
ENSG00000130768	-1.44970113	0.01047367	SMPDL3B
ENSG00000134874	-1.44970113	0.01047367	DZIP1

ENSG00000139725	1.083187406	0.010524881	RHOF
ENSG00000152253	-1.099770217	0.010550453	SPC25
ENSG00000013016	-1.228589601	0.010563916	EHD3
ENSG00000280077	-1.345096767	0.010570774	AL353763.2
ENSG00000229619	-1.437320851	0.010695033	MBNL1-AS1
ENSG00000087074	1.073321629	0.010734505	PPP1R15A
ENSG00000270116	-2.062061745	0.010811714	AP001429.1
ENSG00000147036	1.89092152	0.010811714	LANCL3
ENSG00000279662	-2.062061745	0.010811714	AC131649.2
ENSG00000196748	-2.062061745	0.010811714	CLPSL2
ENSG00000284237	-2.062061745	0.010811714	AL356275.1
ENSG00000235568	-2.062061745	0.010811714	NFAM1
ENSG00000135406	1.363552425	0.010860269	PRPH
ENSG00000108515	-1.08596278	0.010898925	ENO3
ENSG00000214708	1.409517323	0.010901385	AC116407.1
ENSG00000121577	1.798583117	0.010958385	POPDC2
ENSG00000138685	1.057884486	0.011019264	FGF2
ENSG00000166145	1.062613097	0.011052952	SPINT1
ENSG00000127589	-1.304439727	0.01112167	TUBBP1
ENSG00000134470	-1.109344288	0.01112291	IL15RA
ENSG00000142459	1.079242363	0.011140651	EVI5L
ENSG00000273729	1.523361958	0.011159098	AC007686.3
ENSG00000215414	-1.512035625	0.011159098	PSMA6P1
ENSG00000257740	1.523361958	0.011159098	AC073896.3
ENSG00000153132	1.08285514	0.011399989	CLGN
ENSG00000170500	1.077274286	0.011433164	LONRF2
ENSG00000187017	1.186070479	0.011449756	ESPN
ENSG00000185483	1.124590673	0.011613127	ROR1
ENSG00000143344	1.275150547	0.011674865	RGL1
ENSG00000250919	-1.285288243	0.011674865	UGT2B26P
ENSG00000230896	-1.477943601	0.011696955	AL604028.1
ENSG00000163126	2.141922368	0.011803031	ANKRD23
ENSG00000281909	-2.280068216	0.011803031	HERC2P7
ENSG00000243368	-2.280068216	0.011803031	MCCC1-AS1
ENSG00000254207	-2.280068216	0.011803031	AC100797.1
ENSG00000267641	-2.280068216	0.011803031	BNIP3P16
ENSG00000169432	-2.280068216	0.011803031	SCN9A
ENSG00000270000	2.689054364	0.011832427	AC005479.2
ENSG00000186377	-2.769048732	0.011832427	CYP4X1
ENSG00000135917	-2.769048732	0.011832427	SLC19A3
ENSG00000279673	2.689054364	0.011832427	AC092919.2
ENSG00000267741	2.689054364	0.011832427	UBE2L4
ENSG00000254985	-2.769048732	0.011832427	RSF1-IT2
ENSG00000266533	-2.769048732	0.011832427	MIR3619
ENSG00000114812	-2.769048732	0.011832427	VIPR1
ENSG00000258245	-2.769048732	0.011832427	RPL10P13
ENSG00000228808	-2.769048732	0.011832427	HMGB3P4
ENSG00000124194	2.689054364	0.011832427	GDAP1L1
ENSG00000170482	-1.324071563	0.011889469	SLC23A1
ENSG00000132522	1.218132068	0.011940437	GPS2
ENSG00000101213	1.151659074	0.012006874	PTK6
ENSG00000168517	-1.205342062	0.012118943	HEXIM2
ENSG00000130045	1.129307064	0.012132499	NXNL2
ENSG00000163701	-1.540530161	0.012302827	IL17RE
ENSG00000255153	1.615724547	0.012302827	TOLLIP-AS1
ENSG00000275993	1.049189925	0.012331965	SIK1B
ENSG00000160796	-1.071663877	0.012343122	NBEAL2
ENSG00000171798	1.078513945	0.012380622	KNDC1
ENSG00000185477	-1.216877177	0.012410984	GPRIN3
ENSG00000260352	-1.71766873	0.012495401	AC092287.1
ENSG00000275395	-1.517601311	0.012652254	FCGBP

ENSG00000174939	-1.517601311	0.012652254	ASPHD1
ENSG00000257137	5.951733813	0.012730848	C12orf80
ENSG00000265750	-6.096758574	0.012730848	AC090772.3
ENSG00000271573	5.951733813	0.012730848	AL136982.5
ENSG00000279637	-6.096758574	0.012730848	AC018445.3
ENSG00000224448	5.951733813	0.012730848	AC005020.1
ENSG00000252759	5.951733813	0.012730848	RF00019
ENSG00000157404	-6.096758574	0.012730848	KIT
ENSG00000237870	-6.096758574	0.012730848	AC073130.1
ENSG00000166428	5.951733813	0.012730848	PLD4
ENSG00000161643	5.951733813	0.012730848	SIGLEC16
ENSG00000226673	5.951733813	0.012730848	LINC01108
ENSG00000277170	5.951733813	0.012730848	AC012676.3
ENSG00000150337	5.951733813	0.012730848	FCGR1A
ENSG00000115297	5.951733813	0.012730848	TLX2
ENSG00000231441	-6.096758574	0.012730848	AL512422.1
ENSG00000259402	5.951733813	0.012730848	AC090515.5
ENSG00000227527	5.951733813	0.012730848	AC096540.1
ENSG00000114455	-6.096758574	0.012730848	HHLA2
ENSG00000250842	-6.096758574	0.012730848	AC137770.1
ENSG00000237819	5.951733813	0.012730848	AC002454.1
ENSG00000267644	5.951733813	0.012730848	AC061975.7
ENSG00000248557	-6.096758574	0.012730848	AC024558.1
ENSG00000225489	-6.096758574	0.012730848	AL354707.1
ENSG00000264717	5.951733813	0.012730848	NPY4R2
ENSG00000245311	5.951733813	0.012730848	ARNTL2-AS1
ENSG00000272542	-6.096758574	0.012730848	AL137246.1
ENSG00000267607	5.951733813	0.012730848	AC011511.5
ENSG00000199150	-6.096758574	0.012730848	MIRLET7G
ENSG00000201458	5.951733813	0.012730848	RNU4-4P
ENSG00000273403	5.951733813	0.012730848	AC107294.3
ENSG00000163623	-6.096758574	0.012730848	NKX6-1
ENSG00000206989	-6.096758574	0.012730848	SNORD63
ENSG00000082293	-6.096758574	0.012730848	COL19A1
ENSG00000226193	5.951733813	0.012730848	AL049548.1
ENSG00000237927	-6.096758574	0.012730848	AL078604.2
ENSG00000269899	5.951733813	0.012730848	AC025857.2
ENSG00000275450	-6.096758574	0.012730848	AL845472.1
ENSG00000234699	5.951733813	0.012730848	AL139339.1
ENSG00000260693	-6.096758574	0.012730848	AC026150.1
ENSG00000252268	-6.096758574	0.012730848	RNA5SP417
ENSG00000276250	-6.096758574	0.012730848	AC127024.6
ENSG00000253730	5.951733813	0.012730848	AC015909.2
ENSG00000246731	-6.096758574	0.012730848	MGC16275
ENSG00000280486	-6.096758574	0.012730848	AC005329.3
ENSG00000117122	-6.096758574	0.012730848	MFAP2
ENSG00000207606	5.951733813	0.012730848	MIR554
ENSG00000237975	-6.096758574	0.012730848	FLG-AS1
ENSG00000233985	-6.096758574	0.012730848	LINC01681
ENSG00000073756	-6.096758574	0.012730848	PTGS2
ENSG00000241218	-6.096758574	0.012730848	AC063944.2
ENSG00000170516	-6.096758574	0.012730848	COX7B2
ENSG00000248632	-6.096758574	0.012730848	AC106872.5
ENSG00000277411	5.951733813	0.012730848	RF00001
ENSG00000235033	5.951733813	0.012730848	AL590999.1
ENSG00000235139	-6.096758574	0.012730848	AC003984.1
ENSG00000091482	-6.096758574	0.012730848	SMPX
ENSG00000197565	-6.096758574	0.012730848	COL4A6
ENSG00000234176	5.951733813	0.012730848	HSPA8P1
ENSG00000285987	-6.096758574	0.012730848	AL157886.1
ENSG00000237679	5.951733813	0.012730848	VDAC1P11

ENSG00000227355	-6.096758574	0.012730848	AL359644.1
ENSG00000256824	-6.096758574	0.012730848	AP000721.2
ENSG00000171561	-6.096758574	0.012730848	OR2AT4
ENSG00000274209	-6.096758574	0.012730848	ANTXRL
ENSG00000229417	-6.096758574	0.012730848	NPM1P25
ENSG00000231508	5.951733813	0.012730848	RPL34P20
ENSG00000148735	-6.096758574	0.012730848	PLEKHS1
ENSG00000257037	-6.096758574	0.012730848	RARSP1
ENSG00000246331	5.951733813	0.012730848	AC010198.1
ENSG00000258096	5.951733813	0.012730848	AC025031.2
ENSG00000140279	5.951733813	0.012730848	DUOX2
ENSG00000276807	5.951733813	0.012730848	AC022188.1
ENSG00000260086	-6.096758574	0.012730848	AC007611.1
ENSG00000277579	-6.096758574	0.012730848	AC244093.3
ENSG00000274308	-6.096758574	0.012730848	AC244093.1
ENSG00000204652	5.951733813	0.012730848	RPS26P8
ENSG00000221887	-6.096758574	0.012730848	HMSD
ENSG00000104804	-6.096758574	0.012730848	TULP2
ENSG00000099954	-6.096758574	0.012730848	CECR2
ENSG00000146674	1.032862689	0.01276863	IGFBP3
ENSG00000174899	-1.497211351	0.01296303	PQLC2L
ENSG00000231616	1.529242399	0.01296303	AL354733.2
ENSG00000105672	1.329550775	0.012997976	ETV2
ENSG00000197903	1.044905725	0.01300359	HIST1H2BK
ENSG00000142178	1.123686087	0.01303172	SIK1
ENSG00000106333	-1.242043932	0.01314646	PCOLCE
ENSG00000228906	1.791773268	0.013149317	AL353804.1
ENSG00000186952	-1.670400973	0.013149317	TMEM232
ENSG00000278385	1.791773268	0.013149317	AC121338.2
ENSG00000275532	-1.670400973	0.013149317	AC006449.2
ENSG00000229057	1.964976181	0.01323105	RPS3AP54
ENSG00000203279	1.964976181	0.01323105	AL590705.1
ENSG00000175093	1.964976181	0.01323105	SPSB4
ENSG00000273369	-2.118403026	0.01323105	AC096586.2
ENSG00000255045	-2.118403026	0.01323105	AP000866.5
ENSG00000221540	-3.444117691	0.013412089	MIR1180
ENSG00000008438	-3.444117691	0.013412089	PGLYRP1
ENSG00000106823	3.167545026	0.013412089	ECM2
ENSG00000230709	3.167545026	0.013412089	AC104024.1
ENSG00000261460	3.167545026	0.013412089	AC009690.2
ENSG00000260430	3.167545026	0.013412089	AC099518.1
ENSG00000158164	3.167545026	0.013412089	TMSB15A
ENSG00000227258	-3.444117691	0.013412089	SMIM2-AS1
ENSG00000285294	3.167545026	0.013412089	LINC00842
ENSG00000197588	3.167545026	0.013412089	KLKP1
ENSG00000148377	-3.444117691	0.013412089	IDI2
ENSG00000166596	-3.444117691	0.013412089	CFAP52
ENSG00000240087	-3.444117691	0.013412089	RPSAP12
ENSG00000167476	-3.444117691	0.013412089	JSRP1
ENSG00000281530	-3.444117691	0.013412089	AC004461.2
ENSG00000235370	3.167545026	0.013412089	DNM1P51
ENSG00000272182	-3.444117691	0.013412089	AC135507.1
ENSG00000253213	3.167545026	0.013412089	AC010306.1
ENSG00000260448	-3.444117691	0.013412089	LCMT1-AS1
ENSG00000056487	3.167545026	0.013412089	PHF21B
ENSG00000227082	-3.444117691	0.013412089	AC244021.1
ENSG00000285588	-3.444117691	0.013412089	AL160254.1
ENSG00000273199	3.167545026	0.013412089	AP000692.2
ENSG00000273117	-1.462523513	0.013488043	AC144652.1
ENSG00000277150	1.229043798	0.013557267	F8A3
ENSG00000256120	-1.184852479	0.013699481	SOX5-AS1

ENSG00000140465	1.75692161	0.013705036	CYP1A1
ENSG00000172650	-1.630435234	0.013705036	AGAP5
ENSG00000217130	-1.630435234	0.013705036	AL139100.1
ENSG00000267201	1.714021153	0.013705036	LINC01775
ENSG00000261305	-1.630435234	0.013705036	AC005586.2
ENSG00000263847	-1.447645623	0.013710255	AP005899.1
ENSG00000223829	1.095310759	0.013993508	AC004870.2
ENSG00000144355	-1.421751137	0.01408992	DLX1
ENSG00000085514	-1.382994689	0.01425241	PILRA
ENSG00000107719	-1.079147052	0.014352693	PALD1
ENSG00000272808	-1.998267853	0.014380224	AC015712.6
ENSG00000230882	1.832342036	0.014380224	AC005077.4
ENSG00000119508	1.832342036	0.014380224	NR4A3
ENSG00000270379	1.832342036	0.014380224	HEATR9
ENSG00000086506	-1.373562912	0.014399462	HBQ1
ENSG00000016402	2.310370326	0.014485426	IL20RA
ENSG00000116741	-2.418747372	0.014485426	RGS2
ENSG00000241313	2.310370326	0.014485426	WWTR1-AS1
ENSG00000266876	-2.418747372	0.014485426	AC104996.2
ENSG00000174527	2.310370326	0.014485426	MYO1H
ENSG00000162032	2.310370326	0.014485426	SPSB3
ENSG00000141753	2.310370326	0.014485426	IGFBP4
ENSG00000241570	-2.418747372	0.014485426	PAQR9-AS1
ENSG00000256982	2.310370326	0.014485426	AC135782.1
ENSG00000237976	-2.418747372	0.014485426	AL391069.3
ENSG00000272910	-2.418747372	0.014485426	AC090425.2
ENSG00000233030	-2.418747372	0.014485426	AC243772.2
ENSG00000151617	1.630711436	0.01458372	EDNRA
ENSG00000234626	1.32468599	0.014653994	AL021937.3
ENSG00000272631	1.124627147	0.014658678	AC067750.1
ENSG00000019144	-1.020989708	0.014666943	PHLDB1
ENSG00000227232	1.112090643	0.014749201	WASH7P
ENSG00000072571	-1.044747188	0.014763449	HMMR
ENSG00000013588	1.022101431	0.014773025	GPRC5A
ENSG00000164197	1.190236965	0.014783309	RNF180
ENSG00000101882	1.053941913	0.014800722	NKAP
ENSG00000129474	1.013519524	0.014820181	AJUBA
ENSG00000260401	1.162160884	0.014914409	AP002761.4
ENSG00000114805	-1.077803036	0.014988656	PLCH1
ENSG00000163449	-1.135959068	0.01513802	TMEM169
ENSG00000185432	-1.273103746	0.015188732	METTL7A
ENSG00000146038	-1.007750749	0.015198649	DCDC2
ENSG00000261488	1.535989746	0.015229728	TBILA
ENSG00000160404	-1.062926038	0.015249557	TOR2A
ENSG00000158691	1.110010683	0.015301244	ZSCAN12
ENSG00000107518	-1.715865141	0.015305394	ATRNL1
ENSG00000229915	1.728823821	0.015305394	AC016999.1
ENSG00000251577	1.282315271	0.015313558	AC105460.2
ENSG00000240204	1.282315271	0.015313558	SMKR1
ENSG00000180592	-1.246429166	0.015313558	SKIDA1
ENSG00000160932	-1.01406675	0.015334723	LY6E
ENSG00000132801	1.270325999	0.015368674	ZSWIM3
ENSG00000105499	1.258992039	0.015419516	PLA2G4C
ENSG00000173727	-1.088828279	0.015469651	AP000769.1
ENSG00000155961	1.496916364	0.015487289	RAB39B
ENSG00000277639	-1.461691489	0.015487289	AC007906.2
ENSG00000168679	-1.232033022	0.015587243	SLC16A4
ENSG00000130707	-1.006687983	0.015637966	ASS1
ENSG00000038427	-1.001648356	0.015674729	VCAN
ENSG00000265763	-1.445106135	0.015709919	ZNF488
ENSG00000118523	1.151364753	0.015957837	CTGF

ENSG00000021826	1.039796043	0.01597507	CPS1
ENSG00000276715	-1.663463648	0.016051556	YWHAEP7
ENSG00000108821	-1.416697683	0.01607015	COL1A1
ENSG00000099812	1.003088065	0.016183759	MISP
ENSG00000188706	1.013084798	0.016257036	ZDHHC9
ENSG00000279369	2.068417221	0.016492881	AC046185.3
ENSG00000132010	-2.198161566	0.016492881	ZNF20
ENSG00000199719	2.068417221	0.016492881	RN7SKP74
ENSG00000144130	2.068417221	0.016492881	NT5DC4
ENSG00000280739	-2.198161566	0.016492881	EIF1B-AS1
ENSG00000272721	2.068417221	0.016492881	AC131235.3
ENSG00000275383	2.068417221	0.016492881	AC126773.4
ENSG00000177359	2.068417221	0.016492881	AC024940.1
ENSG00000108684	-2.198161566	0.016492881	ASIC2
ENSG00000078237	2.068417221	0.016492881	TIGAR
ENSG00000171217	-2.198161566	0.016492881	CLDN20
ENSG00000228526	-2.198161566	0.016492881	MIR34AHG
ENSG00000196811	2.068417221	0.016492881	CHRNG
ENSG00000254577	-2.198161566	0.016492881	AC087276.1
ENSG00000114251	-2.198161566	0.016492881	WNT5A
ENSG00000173175	-1.619984789	0.016654638	ADCY5
ENSG00000113532	-1.619984789	0.016654638	ST8SIA4
ENSG00000235280	1.744670479	0.016654638	MCF2L-AS1
ENSG00000196951	-1.619984789	0.016654638	SCOC-AS1
ENSG00000126970	1.037836623	0.016664031	ZC4H2
ENSG00000152439	1.04336333	0.016769388	ZNF773
ENSG00000124313	1.018405347	0.016788601	IQSEC2
ENSG00000254438	1.270940943	0.016825845	AC022240.1
ENSG00000061656	-1.266720774	0.016825845	SPAG4
ENSG00000253106	-1.262780737	0.016874371	AC090198.1
ENSG00000071282	-1.255632621	0.016953164	LMCD1
ENSG00000100626	-1.224364836	0.017055548	GALNT16
ENSG00000254300	1.221807446	0.017098491	LINC01111
ENSG00000160469	-1.213374747	0.017121806	BRSK1
ENSG00000260231	1.178915319	0.017122612	KDM7A-DT
ENSG00000276523	1.669805794	0.017143019	AC025287.3
ENSG00000263603	-1.583312827	0.017143019	AC127024.2
ENSG00000185010	1.062702591	0.017146402	F8
ENSG00000172985	1.022953765	0.017505433	SH3RF3
ENSG00000158813	1.648458056	0.017539069	EDA
ENSG00000259683	1.648458056	0.017539069	AC243562.2
ENSG00000175155	-1.102494461	0.017780244	YPEL2
ENSG00000235499	1.592371621	0.017860441	AC073046.1
ENSG00000167077	1.592371621	0.017860441	MEI1
ENSG00000265656	2.590413122	0.017862218	AC106037.2
ENSG00000169896	2.590413122	0.017862218	ITGAM
ENSG00000144550	2.590413122	0.017862218	CPNE9
ENSG00000257718	-2.654658232	0.017862218	CPNE8-AS1
ENSG00000011677	-2.654658232	0.017862218	GABRA3
ENSG00000280273	2.590413122	0.017862218	AF131216.4
ENSG00000234593	-2.654658232	0.017862218	KAZN-AS1
ENSG00000164037	-2.654658232	0.017862218	SLC9B1
ENSG00000285662	2.590413122	0.017862218	AL731733.1
ENSG00000242391	-2.654658232	0.017862218	AC099677.4
ENSG00000207547	-2.654658232	0.017862218	MIR25
ENSG00000223551	-2.654658232	0.017862218	TMSB4XP4
ENSG00000196408	-2.654658232	0.017862218	NOXO1
ENSG00000213903	1.02809965	0.01787743	LTB4R
ENSG00000183682	-2.044850086	0.018003167	BMP8A
ENSG00000180739	-2.044850086	0.018003167	S1PR5
ENSG00000262198	-1.04117121	0.018034431	AL157931.1

ENSG00000231340	1.543811051	0.018121122	ACTG1P10
ENSG00000271452	-1.460623251	0.018121122	AC005034.5
ENSG00000272630	-1.460623251	0.018121122	AL731563.3
ENSG00000139200	-1.460623251	0.018121122	PIANP
ENSG00000104967	1.055672448	0.018338013	NOVA2
ENSG00000279041	-1.1453307	0.018451844	AC102945.2
ENSG00000186451	1.463849375	0.018502711	SPATA12
ENSG00000114854	1.463849375	0.018502711	TNNC1
ENSG00000235510	1.430515054	0.018639691	BX842568.4
ENSG00000264920	-1.397111584	0.018748976	AC018521.5
ENSG00000115423	-1.397111584	0.018748976	DNAH6
ENSG00000168646	-1.198996156	0.018821439	AXIN2
ENSG00000267681	1.373789042	0.018835279	AC135721.1
ENSG00000086570	-1.385071285	0.018835279	FAT2
ENSG00000184100	-1.355044696	0.018991473	BRD7P2
ENSG00000186007	-1.355044696	0.018991473	LEMD1
ENSG00000128284	-1.231355354	0.018992235	APOL3
ENSG00000254815	-1.233976183	0.019009984	AP006284.1
ENSG00000116254	-1.233976183	0.019009984	CHD5
ENSG00000259342	-1.31923675	0.019018094	AC025580.1
ENSG00000283236	-1.312503817	0.019035303	AC074141.1
ENSG00000125735	-1.312503817	0.019035303	TNFSF14
ENSG00000244649	-1.239740856	0.01903659	LINC02086
ENSG00000183054	-1.295138606	0.019044269	RGPD6
ENSG00000197813	-1.300506288	0.019047235	AC011450.1
ENSG00000100234	1.771282942	0.019137366	TIMP3
ENSG00000225613	1.771282942	0.019137366	LINCMD1
ENSG00000271314	1.771282942	0.019137366	AL161729.2
ENSG00000269044	1.118482984	0.019544006	AC024075.2
ENSG00000236438	1.082120595	0.019716935	FAM157A
ENSG00000234281	-1.654773074	0.019987884	LANCL1-AS1
ENSG00000141540	1.672530389	0.019987884	TTYH2
ENSG00000109927	1.672530389	0.019987884	TECTA
ENSG00000131477	-1.844092128	0.019987884	RAMP2
ENSG00000231566	1.672530389	0.019987884	LINC02595
ENSG00000101331	-1.654773074	0.019987884	CCM2L
ENSG00000215146	-1.006317012	0.020812945	BX322639.1
ENSG00000115232	-1.182899523	0.02085069	ITGA4
ENSG00000157399	-1.010488013	0.02093427	ARSE
ENSG00000227449	2.223598793	0.020952111	FGF7P6
ENSG00000183508	-2.320020027	0.020952111	TENT5C
ENSG00000232742	2.223598793	0.020952111	RHOQP2
ENSG00000180846	-2.320020027	0.020952111	CSNK1G2-AS1
ENSG00000231993	-2.320020027	0.020952111	EP300-AS1
ENSG00000132000	2.223598793	0.020952111	PODNL1
ENSG00000284828	2.223598793	0.020952111	AC012020.2
ENSG00000285679	2.223598793	0.020952111	AC097626.1
ENSG00000154096	2.223598793	0.020952111	THY1
ENSG00000234134	-2.320020027	0.020952111	AL158835.2
ENSG00000226469	-2.320020027	0.020952111	ADAM1B
ENSG00000197558	2.223598793	0.020952111	SSPO
ENSG00000257542	2.223598793	0.020952111	OR7E47P
ENSG00000176124	-1.070067923	0.021047195	DLEU1
ENSG00000279744	-1.567742771	0.021096335	AC132938.5
ENSG00000236991	1.695977768	0.021096335	EDRF1-AS1
ENSG00000179698	1.695977768	0.021096335	WDR97
ENSG00000168071	1.038005695	0.021354195	CCDC88B
ENSG00000137880	-1.53459915	0.021443929	GCHFR
ENSG00000270030	-1.53459915	0.021443929	AC136475.7
ENSG00000275759	-3.308152559	0.021566548	AC026367.3
ENSG00000260369	3.031743203	0.021566548	AC120024.1

ENSG00000234155	-3.308152559	0.021566548	LINC02535
ENSG00000263280	-3.308152559	0.021566548	AC003965.1
ENSG00000128713	-3.308152559	0.021566548	HOXD11
ENSG00000276368	3.031743203	0.021566548	HIST1H2AJ
ENSG00000237641	3.031743203	0.021566548	AC073476.1
ENSG00000172940	3.031743203	0.021566548	SLC22A13
ENSG00000248161	-3.308152559	0.021566548	AC098487.1
ENSG00000225726	-3.308152559	0.021566548	AC007000.2
ENSG00000253771	3.031743203	0.021566548	TPTE2P1
ENSG00000265100	-3.308152559	0.021566548	AC005332.1
ENSG00000102001	3.031743203	0.021566548	CACNA1F
ENSG00000167800	3.031743203	0.021566548	TBX10
ENSG00000279641	-3.308152559	0.021566548	AC120057.3
ENSG00000277501	3.031743203	0.021566548	AC243571.2
ENSG00000217442	-3.308152559	0.021566548	SYCE3
ENSG00000162746	-3.308152559	0.021566548	FCRLB
ENSG00000238005	3.031743203	0.021566548	AL391832.2
ENSG00000170959	3.031743203	0.021566548	DCDC1
ENSG00000283611	3.031743203	0.021566548	AL357140.4
ENSG00000244265	3.031743203	0.021566548	SIAH2-AS1
ENSG00000227344	3.031743203	0.021566548	HAUS6P1
ENSG00000254710	-3.308152559	0.021566548	AP001970.1
ENSG00000179111	-3.308152559	0.021566548	HES7
ENSG00000226780	-3.308152559	0.021566548	AC244035.1
ENSG00000273416	3.031743203	0.021566548	AL732292.2
ENSG00000283782	3.031743203	0.021566548	AC116366.3
ENSG00000252355	3.031743203	0.021566548	RN7SKP287
ENSG00000260668	-3.308152559	0.021566548	AC093536.1
ENSG00000206762	-3.308152559	0.021566548	RNU6-418P
ENSG00000232166	3.031743203	0.021566548	AL122008.2
ENSG00000264324	-3.308152559	0.021566548	AC006030.1
ENSG00000175772	3.031743203	0.021566548	LINC01106
ENSG00000255145	-3.308152559	0.021566548	STX17-AS1
ENSG00000263033	3.031743203	0.021566548	AC007220.1
ENSG00000272079	1.563896092	0.02169529	AC004233.3
ENSG00000174514	1.563896092	0.02169529	MFSD4A
ENSG00000228022	1.606796549	0.02169529	HCG20
ENSG00000270084	-1.50632397	0.02169529	GAS5-AS1
ENSG00000130643	-1.285596434	0.021782468	CALY
ENSG00000015285	1.55298504	0.021872151	WAS
ENSG00000157890	-1.437680878	0.021872151	MEGF11
ENSG00000285763	1.55298504	0.021872151	AL358777.1
ENSG00000163531	-1.437680878	0.021872151	NFASC
ENSG00000228589	-1.064655334	0.021912667	SPCS2P4
ENSG00000105427	-1.3265425	0.021919674	CNFN
ENSG00000084734	1.299884393	0.021979843	GCKR
ENSG00000188649	-1.418946397	0.021991065	CC2D2B
ENSG00000172935	-1.418946397	0.021991065	MRGPRF
ENSG00000173825	1.320951581	0.02203199	TIGD3
ENSG00000167798	-1.343192537	0.02203199	C3P1
ENSG00000196350	-1.019233416	0.022054178	ZNF729
ENSG00000235865	1.465825334	0.022064739	GSN-AS1
ENSG00000274849	1.344122065	0.022073746	AC023043.4
ENSG00000244479	-1.36325875	0.022102148	OR2A1-AS1
ENSG00000162552	1.398189019	0.022113468	WNT4
ENSG00000153975	-1.000066211	0.022284351	ZUP1
ENSG00000246859	-1.161868609	0.022421406	STARD4-AS1
ENSG00000256806	1.155901628	0.022653858	C17orf100
ENSG00000264859	5.762414759	0.022676824	DSG2-AS1
ENSG00000266258	5.762414759	0.022676824	LINC01909
ENSG00000273777	-5.907121764	0.022676824	CEACAM20

ENSG00000283064	5.762414759	0.022676824	AL353759.1
ENSG00000174130	-5.907121764	0.022676824	TLR6
ENSG00000278635	-5.907121764	0.022676824	AC141557.2
ENSG00000271259	5.762414759	0.022676824	AC010201.1
ENSG00000260765	5.762414759	0.022676824	CES1P2
ENSG00000254780	5.762414759	0.022676824	AC023232.1
ENSG00000250892	-5.907121764	0.022676824	AC108475.1
ENSG00000279511	-5.907121764	0.022676824	AL356274.2
ENSG00000271002	5.762414759	0.022676824	AC129492.5
ENSG00000080572	5.762414759	0.022676824	PIH1D3
ENSG00000280129	-5.907121764	0.022676824	AL132780.5
ENSG00000278009	-5.907121764	0.022676824	AL139099.3
ENSG00000006638	5.762414759	0.022676824	TBXA2R
ENSG00000259946	5.762414759	0.022676824	BX005019.1
ENSG00000270528	5.762414759	0.022676824	AC021171.1
ENSG00000227245	5.762414759	0.022676824	AC092042.1
ENSG00000222389	5.762414759	0.022676824	RNU2-28P
ENSG00000245067	-5.907121764	0.022676824	IGFBP7-AS1
ENSG00000264448	5.762414759	0.022676824	AC084346.2
ENSG00000234537	-5.907121764	0.022676824	AL354751.1
ENSG00000254480	-5.907121764	0.022676824	AC015689.1
ENSG00000255538	-5.907121764	0.022676824	OR10V2P
ENSG00000270926	-5.907121764	0.022676824	AC008013.3
ENSG00000201208	-5.907121764	0.022676824	RF00019
ENSG00000258608	-5.907121764	0.022676824	DNAJC19P9
ENSG00000244056	5.762414759	0.022676824	RN7SL417P
ENSG00000226835	5.762414759	0.022676824	AC097059.1
ENSG00000280294	-5.907121764	0.022676824	AC011008.2
ENSG00000174885	5.762414759	0.022676824	NLRP6
ENSG00000189431	-5.907121764	0.022676824	RASSF10
ENSG00000183230	5.762414759	0.022676824	CTNNA3
ENSG00000010610	5.762414759	0.022676824	CD4
ENSG00000214203	-5.907121764	0.022676824	RPS4XP1
ENSG00000258789	-5.907121764	0.022676824	AL162171.1
ENSG00000262481	5.762414759	0.022676824	TMEM256-PLSCR3
ENSG00000269404	5.762414759	0.022676824	SPIB
ENSG00000268307	-5.907121764	0.022676824	LINC02560
ENSG00000236754	-5.907121764	0.022676824	AC007666.1
ENSG00000221643	5.762414759	0.022676824	SNORA77
ENSG00000143632	-5.907121764	0.022676824	ACTA1
ENSG00000232059	-5.907121764	0.022676824	AL451007.1
ENSG00000273240	-5.907121764	0.022676824	AC013468.1
ENSG00000227542	-5.907121764	0.022676824	AC092614.1
ENSG00000235904	-5.907121764	0.022676824	RBMS3-AS3
ENSG00000168356	5.762414759	0.022676824	SCN11A
ENSG00000243715	-5.907121764	0.022676824	CACNA2D3-AS1
ENSG00000232615	5.762414759	0.022676824	AC026412.2
ENSG00000091010	5.762414759	0.022676824	POU4F3
ENSG00000285269	5.762414759	0.022676824	AL160269.1
ENSG00000233515	5.762414759	0.022676824	LINC01518
ENSG00000257443	-5.907121764	0.022676824	AC068305.2
ENSG00000277945	5.762414759	0.022676824	AC107308.1
ENSG00000244060	5.762414759	0.022676824	RPS2P41
ENSG00000275485	-5.907121764	0.022676824	AL512652.1
ENSG00000153495	5.762414759	0.022676824	TEX29
ENSG00000198535	-5.907121764	0.022676824	C2CD4A
ENSG00000279415	5.762414759	0.022676824	AC099494.2
ENSG00000262141	-5.907121764	0.022676824	AC040162.2
ENSG00000234203	-5.907121764	0.022676824	AC004771.2
ENSG00000161940	-5.907121764	0.022676824	BCL6B
ENSG00000214553	-5.907121764	0.022676824	LRRC37A11P

ENSG00000060558	5.762414759	0.022676824	GNA15
ENSG00000281383	5.762414759	0.022676824	FP671120.4
ENSG00000284879	5.762414759	0.022676824	AC133644.3
ENSG00000224087	-5.907121764	0.022676824	AC018865.1
ENSG00000245322	-5.907121764	0.022676824	AC097460.1
ENSG00000271849	-5.907121764	0.022676824	AC012603.1
ENSG00000249526	-5.907121764	0.022676824	AC008667.1
ENSG00000229214	5.762414759	0.022676824	LINC00242
ENSG00000283431	5.762414759	0.022676824	AC099654.6
ENSG00000284292	5.762414759	0.022676824	AC004922.1
ENSG00000106278	-5.907121764	0.022676824	PTPRZ1
ENSG00000254923	5.762414759	0.022676824	AC130366.1
ENSG00000282886	-5.907121764	0.022676824	AL691447.3
ENSG00000182782	-5.907121764	0.022676824	HCAR2
ENSG00000226359	-5.907121764	0.022676824	ACTG1P24
ENSG00000125398	-5.907121764	0.022676824	SOX9
ENSG00000150656	5.762414759	0.022676824	CNDP1
ENSG00000283142	5.762414759	0.022676824	AL049767.1
ENSG00000236527	5.762414759	0.022676824	ARF4P2
ENSG00000186204	5.762414759	0.022676824	CYP4F12
ENSG00000269040	5.762414759	0.022676824	BNIP3P24
ENSG00000224927	-5.907121764	0.022676824	NDUFA5P10
ENSG00000234232	5.762414759	0.022676824	AC243772.3
ENSG00000236484	5.762414759	0.022676824	RRM2P2
ENSG00000081277	5.762414759	0.022676824	PKP1
ENSG00000261000	-5.907121764	0.022676824	AC244034.2
ENSG00000282418	-5.907121764	0.022676824	AC092811.2
ENSG00000236391	-5.907121764	0.022676824	AC092573.2
ENSG00000227107	5.762414759	0.022676824	AC104667.1
ENSG00000224232	5.762414759	0.022676824	AC005104.2
ENSG00000210181	5.762414759	0.022676824	RNU6ATAC4P
ENSG00000270059	5.762414759	0.022676824	AC121493.1
ENSG00000228221	-5.907121764	0.022676824	LINC00578
ENSG00000273375	5.762414759	0.022676824	AC055764.2
ENSG00000282742	5.762414759	0.022676824	AC093323.3
ENSG00000206629	5.762414759	0.022676824	RNU1-63P
ENSG00000164128	-5.907121764	0.022676824	NPY1R
ENSG00000249776	5.762414759	0.022676824	AC124854.1
ENSG00000249175	5.762414759	0.022676824	AC008534.1
ENSG00000175749	-5.907121764	0.022676824	EIF3KP1
ENSG00000270174	5.762414759	0.022676824	AL022097.1
ENSG00000199851	5.762414759	0.022676824	RF00012
ENSG00000213542	5.762414759	0.022676824	AC007000.1
ENSG00000237818	5.762414759	0.022676824	RPS3AP29
ENSG00000164604	-5.907121764	0.022676824	GPR85
ENSG00000229591	5.762414759	0.022676824	AC006017.1
ENSG00000279601	5.762414759	0.022676824	AC005052.1
ENSG00000189420	5.762414759	0.022676824	ZFP92
ENSG00000284957	5.762414759	0.022676824	AF131216.5
ENSG00000253648	-5.907121764	0.022676824	AC018437.2
ENSG00000254305	-5.907121764	0.022676824	MRPL9P1
ENSG00000254364	-5.907121764	0.022676824	AP000424.2
ENSG00000153707	-5.907121764	0.022676824	PTPRD
ENSG00000148053	-5.907121764	0.022676824	NTRK2
ENSG00000254900	5.762414759	0.022676824	AC091053.2
ENSG00000180745	-5.907121764	0.022676824	CLRN3
ENSG00000171840	5.762414759	0.022676824	NINJ2
ENSG00000134532	-5.907121764	0.022676824	SOX5
ENSG00000173157	-5.907121764	0.022676824	ADAMTS20
ENSG00000258345	5.762414759	0.022676824	AC034102.7
ENSG00000139767	5.762414759	0.022676824	SRRM4

ENSG00000235438	-5.907121764	0.022676824	ESRRAP2
ENSG00000134873	-5.907121764	0.022676824	CLDN10
ENSG00000225083	-5.907121764	0.022676824	GRTP1-AS1
ENSG00000139915	-5.907121764	0.022676824	MDGA2
ENSG00000258510	-5.907121764	0.022676824	AC007954.1
ENSG00000222990	5.762414759	0.022676824	RNU4-22P
ENSG00000259052	5.762414759	0.022676824	AL157871.6
ENSG00000137860	5.762414759	0.022676824	SLC28A2
ENSG00000259750	-5.907121764	0.022676824	AC092868.3
ENSG00000166104	-5.907121764	0.022676824	AC126323.1
ENSG00000137809	5.762414759	0.022676824	ITGA11
ENSG00000277602	5.762414759	0.022676824	AC005363.2
ENSG00000198106	-5.907121764	0.022676824	SNX29P2
ENSG00000103490	-5.907121764	0.022676824	PYCARD
ENSG00000182557	-5.907121764	0.022676824	SPNS3
ENSG00000233090	-5.907121764	0.022676824	AC015922.1
ENSG00000264932	5.762414759	0.022676824	AC115989.1
ENSG00000230258	-5.907121764	0.022676824	AC005208.1
ENSG00000272746	-5.907121764	0.022676824	AP005131.7
ENSG00000228835	-5.907121764	0.022676824	AC012123.1
ENSG00000221806	5.762414759	0.022676824	RNU6ATAC34P
ENSG00000174898	5.762414759	0.022676824	CATSPERD
ENSG00000267672	5.762414759	0.022676824	AC010632.2
ENSG00000284654	-5.907121764	0.022676824	AP000553.7
ENSG00000224598	-5.907121764	0.022676824	RPS5P2
ENSG00000278198	5.762414759	0.022676824	AC011043.2
ENSG00000278023	-1.059727847	0.022732719	RDM1
ENSG00000268362	-1.105552625	0.022887841	AC092279.1
ENSG00000224383	1.123801706	0.023001377	PRR29
ENSG00000173261	-2.111323418	0.023070854	PLAC8L1
ENSG00000232748	1.990964993	0.023070854	AC135050.1
ENSG00000204613	1.990964993	0.023070854	TRIM10
ENSG00000263680	-2.111323418	0.023070854	AC007639.1
ENSG00000242082	-2.111323418	0.023070854	SLC5A4-AS1
ENSG00000234789	-2.111323418	0.023070854	AL590369.1
ENSG00000249996	1.990964993	0.023070854	AC106786.2
ENSG00000253141	-2.111323418	0.023070854	AC008632.1
ENSG00000221500	-2.111323418	0.023070854	SNORD100
ENSG00000264577	-1.164275544	0.023163897	AC010761.1
ENSG00000137825	1.053989356	0.023165665	ITPKA
ENSG00000113916	-1.054153729	0.023165665	BCL6
ENSG00000238058	-1.164756149	0.023301549	AL355574.1
ENSG00000226287	1.077067661	0.023339508	TMEM191A
ENSG00000229950	1.062004922	0.023451823	TFAP2A-AS1
ENSG00000179630	-1.101322957	0.0234926	LACC1
ENSG00000260877	1.220184515	0.023589839	AP005233.2
ENSG00000273142	1.122260956	0.024051958	AC073335.2
ENSG00000260757	-1.191048145	0.024056027	AC093520.1
ENSG00000064270	-1.967344791	0.024510598	ATP2C2
ENSG00000267160	1.828328809	0.024510598	AC091152.2
ENSG00000268601	1.828328809	0.024510598	AC115522.1
ENSG00000214145	1.828328809	0.024510598	LINC00887
ENSG00000075043	-1.967344791	0.024510598	KCNQ2
ENSG00000259563	-1.967344791	0.024510598	AC025430.1
ENSG00000162946	-1.249985377	0.024563218	DISC1
ENSG00000178695	-1.022141558	0.024652225	KCTD12
ENSG00000106258	-1.258177655	0.024922583	CYP3A5
ENSG00000169442	-1.144629514	0.025050771	CD52
ENSG00000107282	1.154795655	0.025239518	APBA1
ENSG00000279347	1.707525003	0.025475297	AC021945.1
ENSG00000235560	-1.861537855	0.025475297	AC002310.1

ENSG00000279110	1.707525003	0.025475297	AL022323.4
ENSG00000164049	-1.861537855	0.025475297	FBXW12
ENSG00000181585	1.271941304	0.025485094	TMIE
ENSG00000175768	-1.304199545	0.025485094	TOMM5
ENSG00000236603	-1.304199545	0.025485094	RANP1
ENSG00000164056	-1.304199545	0.025485094	SPRY1
ENSG00000254838	1.313832198	0.025864487	GVINP1
ENSG00000279738	-1.319659355	0.025864487	AL022311.1
ENSG00000102452	1.102075088	0.025977013	NALCN
ENSG00000001626	-1.328592432	0.02605029	CFTR
ENSG00000230844	1.08540329	0.026101511	ZNF674-AS1
ENSG00000128340	-1.780298236	0.026105331	RAC2
ENSG00000273724	-1.780298236	0.026105331	AC106782.5
ENSG00000253304	-1.590979182	0.026105331	TMEM200B
ENSG00000170264	-1.000286937	0.026138647	FAM161A
ENSG00000162733	-1.349562597	0.026398783	DDR2
ENSG00000179104	-1.065913204	0.02646279	TMTC2
ENSG00000244668	-1.548532359	0.026497687	SNRPCP3
ENSG00000139540	-1.548532359	0.026497687	SLC39A5
ENSG00000233589	1.468123987	0.026682384	AL138789.1
ENSG00000143028	1.468123987	0.026682384	SYPL2
ENSG00000229932	1.468123987	0.026682384	YWHAZP3
ENSG00000172426	1.468123987	0.026682384	RSPH9
ENSG00000275560	-1.376029667	0.026682384	AC008115.3
ENSG00000213977	1.468123987	0.026682384	TAX1BP3
ENSG00000225422	-1.376029667	0.026682384	RBMS1P1
ENSG00000144668	-1.376029667	0.026682384	ITGA9
ENSG00000223522	-1.376029667	0.026682384	AC093690.1
ENSG00000247595	-1.376029667	0.026682384	SPTY2D1OS
ENSG00000257851	-1.513537688	0.026720022	HNRNPA3P10
ENSG00000231742	-1.513537688	0.026720022	LINC01273
ENSG00000285106	1.512492925	0.026780029	AC016831.6
ENSG00000100311	1.512492925	0.026780029	PDGFB
ENSG00000230982	1.577089585	0.026820067	DSTNP1
ENSG00000240405	1.577089585	0.026820067	SAMMSON
ENSG00000171812	1.577089585	0.026820067	COL8A2
ENSG00000232940	1.577089585	0.026820067	HCG25
ENSG00000218358	1.577089585	0.026820067	RAET1K
ENSG00000137133	1.519680733	0.026831883	HINT2
ENSG00000270673	1.519680733	0.026831883	YTHDF3-AS1
ENSG00000206077	1.519680733	0.026831883	ZDHHC11B
ENSG00000141837	1.519680733	0.026831883	CACNA1A
ENSG00000205702	1.519680733	0.026831883	CYP2D7
ENSG00000135436	-1.459201563	0.026831883	FAM186B
ENSG00000169330	-1.459201563	0.026831883	KIAA1024
ENSG00000243678	-1.061865402	0.0269941	NME2
ENSG00000278396	2.484529265	0.02704271	AL122023.1
ENSG00000281386	2.484529265	0.02704271	AP003500.1
ENSG00000229167	2.484529265	0.02704271	AC114488.1
ENSG00000201563	2.484529265	0.02704271	RF00019
ENSG00000255052	2.484529265	0.02704271	FAM66D
ENSG00000270890	2.484529265	0.02704271	AL049844.2
ENSG00000259322	2.484529265	0.02704271	AC090607.1
ENSG00000257298	2.484529265	0.02704271	AC008147.2
ENSG00000260714	2.484529265	0.02704271	AC133552.1
ENSG00000258593	2.484529265	0.02704271	AL583810.1
ENSG00000253485	-1.167634336	0.02704513	PCDHGA5
ENSG00000113739	1.0005004	0.027194327	STC2
ENSG00000227946	1.182310924	0.027595171	AC007383.2
ENSG00000250303	-1.169069937	0.027595171	AP002884.1
ENSG00000165591	1.010407169	0.027651091	FAAH2

ENSG00000163637	-1.069713906	0.027736032	PRICKLE2
ENSG00000223573	1.039475033	0.027781112	TINCR
ENSG00000227354	1.137544806	0.027813734	RBM26-AS1
ENSG00000184227	-1.22701695	0.028192521	ACOT1
ENSG00000273314	1.077420064	0.028227965	AC005229.4
ENSG00000129535	1.237068925	0.028840875	NRL
ENSG00000131730	1.237068925	0.028840875	CKMT2
ENSG00000233901	-1.144239539	0.028988998	LINC01503
ENSG00000124374	-1.144239539	0.028988998	PAIP2B
ENSG00000134317	1.025965263	0.02910436	GRHL1
ENSG00000185674	1.161395934	0.029317327	LYG2
ENSG00000136383	1.243446286	0.029542889	ALPK3
ENSG00000258077	-1.273251606	0.029542889	AC078923.1
ENSG00000237149	-1.040585529	0.030243749	ZNF503-AS2
ENSG00000164743	-1.285806521	0.030298791	C8orf48
ENSG00000248238	1.282892721	0.030298791	LINC02438
ENSG00000130653	-1.052427771	0.030316257	PNPLA7
ENSG00000054219	-1.052427771	0.030316257	LY75
ENSG00000268262	2.131272471	0.030348148	AC011445.1
ENSG00000206075	2.131272471	0.030348148	SERPINB5
ENSG00000171522	-2.214036947	0.030348148	PTGER4
ENSG00000253496	2.131272471	0.030348148	AC011586.2
ENSG00000160408	-2.214036947	0.030348148	ST6GALNAC6
ENSG00000226370	2.131272471	0.030348148	LINC00375
ENSG00000258388	2.131272471	0.030348148	PPT2-EGFL8
ENSG00000232415	2.131272471	0.030348148	ELN-AS1
ENSG00000259881	2.131272471	0.030348148	AC092384.2
ENSG00000140527	-2.214036947	0.030348148	WDR93
ENSG00000183444	2.131272471	0.030348148	OR7E38P
ENSG00000200356	2.131272471	0.030348148	RNU6-833P
ENSG00000150275	2.131272471	0.030348148	PCDH15
ENSG00000185615	2.131272471	0.030348148	PDIA2
ENSG00000141028	-2.214036947	0.030348148	CDRT15P1
ENSG00000265033	2.131272471	0.030348148	RN7SL262P
ENSG00000144834	-2.214036947	0.030348148	TAGLN3
ENSG00000147573	-2.214036947	0.030348148	TRIM55
ENSG00000240399	-2.214036947	0.030348148	AC004801.2
ENSG00000255339	2.131272471	0.030348148	AL133352.1
ENSG00000184860	-2.214036947	0.030348148	SDR42E1
ENSG00000006747	-1.00703187	0.030369868	SCIN
ENSG00000159403	-1.009462569	0.030387595	C1R
ENSG00000214182	-1.05064821	0.030599236	PTMAP5
ENSG00000274026	-1.293072571	0.030695741	FAM27E3
ENSG00000253598	-1.293072571	0.030695741	SLC10A5
ENSG00000213862	-1.293072571	0.030695741	AC044787.1
ENSG00000181524	-1.008547771	0.030944281	RPL24P4
ENSG00000150510	-1.301143875	0.031103696	FAM124A
ENSG00000258633	1.359842254	0.031520396	AL049870.2
ENSG00000010319	-1.310162299	0.031520396	SEMA3G
ENSG00000137090	1.392073302	0.031942348	DMRT1
ENSG00000165555	-1.320305155	0.031942348	NOXRED1
ENSG00000197140	-1.320305155	0.031942348	ADAM32
ENSG00000210077	-1.320305155	0.031942348	MT-TV
ENSG00000272752	1.392073302	0.031942348	STAG3L5P-PVRIG2P-PILRB
ENSG00000108387	-1.199598171	0.032118678	4-Sep
ENSG00000245614	1.152139603	0.032228494	DDX11-AS1
ENSG00000007402	-1.122903386	0.032228494	CACNA2D2
ENSG00000155093	1.13254582	0.032228494	PTPRN2
ENSG00000240373	1.909117607	0.032292239	SEC62-AS1
ENSG00000186603	-2.018921673	0.032292239	HPDL
ENSG00000225339	1.909117607	0.032292239	AL354740.1

ENSG00000261584	-2.018921673	0.032292239	AL513548.1
ENSG00000201772	-2.018921673	0.032292239	SNORA5C
ENSG00000284948	1.909117607	0.032292239	AC107959.4
ENSG00000135094	-2.018921673	0.032292239	SDS
ENSG00000250602	1.428737406	0.032364314	AC093535.1
ENSG00000152292	-1.331797022	0.032364314	SH2D6
ENSG00000157017	1.428737406	0.032364314	GHRL
ENSG00000112137	1.428737406	0.032364314	PHACTR1
ENSG00000170345	1.083760279	0.032467048	FOS
ENSG00000230797	1.018301863	0.032524508	YY2
ENSG00000265982	1.089659198	0.03287854	AC103810.3
ENSG00000188818	1.053741804	0.032924312	ZDHHC11
ENSG00000241549	-1.233707901	0.033121091	GUSBP2
ENSG00000221955	1.183759391	0.033121091	SLC12A8
ENSG00000225234	1.474067313	0.033173684	TRAPPC12-AS1
ENSG00000275494	1.474067313	0.033173684	AC133552.5
ENSG00000228420	-1.410487887	0.033173684	LINC01768
ENSG00000226330	1.047269444	0.033250706	AL606489.1
ENSG00000270061	-1.885438141	0.033376343	AC068790.5
ENSG00000255566	1.754823662	0.033376343	AC135279.1
ENSG00000267274	1.754823662	0.033376343	AC008770.3
ENSG00000232273	1.754823662	0.033376343	FTH1P1
ENSG00000234857	1.754823662	0.033376343	HNRNPUL2-BSCL2
ENSG00000225762	1.754823662	0.033376343	LINC01389
ENSG00000078795	-1.885438141	0.033376343	PKD2L2
ENSG00000206192	-1.885438141	0.033376343	ANKRD20A9P
ENSG00000237356	1.754823662	0.033376343	AL365295.1
ENSG00000268655	1.754823662	0.033376343	AC008687.4
ENSG00000257681	-1.885438141	0.033376343	AC025265.1
ENSG00000249937	-1.885438141	0.033376343	LINC02223
ENSG00000226284	-1.885438141	0.033376343	ARPC3P1
ENSG00000130775	-1.431940948	0.033533086	THEMIS2
ENSG00000261386	-1.431940948	0.033533086	AC027682.4
ENSG00000213693	1.528396874	0.033533086	SEC14L1P1
ENSG00000257433	-1.431940948	0.033533086	AC004241.1
ENSG00000164404	1.160414404	0.033641772	GDF9
ENSG00000144642	-1.120531547	0.033641772	RBMS3
ENSG00000261072	1.593366135	0.033832332	AC084783.1
ENSG00000130558	-1.457216232	0.033832332	OLFM1
ENSG00000229018	1.593366135	0.033832332	PMS2P7
ENSG00000154898	1.640818562	0.033910222	CCDC144CP
ENSG00000213018	1.640818562	0.033910222	AL590762.1
ENSG00000226763	1.640818562	0.033910222	SRRM5
ENSG00000271714	1.640818562	0.033910222	AC010501.1
ENSG00000274902	-1.787984916	0.033910222	AC004241.2
ENSG00000214243	1.640818562	0.033910222	AC004980.2
ENSG00000231663	1.55289181	0.034088382	AL355472.1
ENSG00000225880	1.55289181	0.034088382	LINC00115
ENSG00000254461	1.55289181	0.034088382	AP001107.4
ENSG00000256050	1.55289181	0.034088382	AL583722.1
ENSG00000241494	-1.713552437	0.034088382	AL355032.1
ENSG00000267192	1.55289181	0.034088382	AC006116.4
ENSG00000260645	-1.524233383	0.034088382	AL359715.2
ENSG00000081181	-1.241625207	0.034237201	ARG2
ENSG00000176894	-1.084877453	0.034238954	PXMP2
ENSG00000135338	1.036548093	0.034570186	LCA5
ENSG00000225506	1.231925548	0.034842559	CYP4A22-AS1
ENSG00000258334	1.231925548	0.034842559	AC125611.4
ENSG00000259659	-1.246154209	0.034842559	AC009996.1
ENSG00000260103	-1.246154209	0.034842559	AC012435.1
ENSG00000267980	2.881817243	0.034866602	AC007292.1

ENSG00000267115	-3.158027498	0.034866602	AC022148.2
ENSG00000250995	2.881817243	0.034866602	AL391280.1
ENSG00000234509	2.881817243	0.034866602	AP000253.1
ENSG00000207008	2.881817243	0.034866602	SNORA54
ENSG00000169884	-3.158027498	0.034866602	WNT10B
ENSG00000146090	-3.158027498	0.034866602	RASGEF1C
ENSG00000273473	-3.158027498	0.034866602	BX649601.1
ENSG00000176009	-3.158027498	0.034866602	ASCL3
ENSG00000285697	-3.158027498	0.034866602	AC002059.2
ENSG00000117643	-3.158027498	0.034866602	MAN1C1
ENSG00000237080	-3.158027498	0.034866602	EHMT2-AS1
ENSG00000232821	2.881817243	0.034866602	AC003986.2
ENSG00000255186	-3.158027498	0.034866602	AC087277.2
ENSG00000229994	-3.158027498	0.034866602	RPL5P4
ENSG00000240057	2.881817243	0.034866602	AC078785.1
ENSG00000151846	-3.158027498	0.034866602	PABPC3
ENSG00000234377	2.881817243	0.034866602	RNF219-AS1
ENSG00000175344	2.881817243	0.034866602	CHRNA7
ENSG00000264012	2.881817243	0.034866602	AC091588.1
ENSG00000227850	-3.158027498	0.034866602	SEPT2P1
ENSG00000236047	-3.158027498	0.034866602	AC073410.1
ENSG00000281593	2.881817243	0.034866602	AC006978.2
ENSG00000285581	2.881817243	0.034866602	AC091196.1
ENSG00000210127	2.881817243	0.034866602	MT-TA
ENSG00000242396	-3.158027498	0.034866602	AC096536.2
ENSG00000126890	-3.158027498	0.034866602	CTAG2
ENSG00000254317	2.881817243	0.034866602	AC022973.4
ENSG00000259986	-3.158027498	0.034866602	AC103876.1
ENSG00000266980	-3.158027498	0.034866602	AC087289.1
ENSG00000267147	-3.158027498	0.034866602	LINC01842
ENSG00000283438	2.881817243	0.034866602	RF00017
ENSG00000210176	-1.024714393	0.034958896	MT-TH
ENSG00000285373	-1.102578032	0.035267165	LINC02478
ENSG00000247516	1.01229785	0.035421272	MIR4458HG
ENSG00000273108	1.25127516	0.035481962	AL121929.2
ENSG00000235313	1.25127516	0.035481962	HM13-IT1
ENSG00000167614	1.067106831	0.035558915	TTYH1
ENSG00000156222	1.112682258	0.035823429	SLC28A1
ENSG00000276846	-1.256656071	0.036157187	AC016590.3
ENSG00000283498	-1.006100646	0.036187098	MIR1244-2
ENSG00000165985	1.296623622	0.036869772	C1QL3
ENSG00000259033	1.323437471	0.037620819	AL356804.1
ENSG00000224621	1.323437471	0.037620819	AL451042.1
ENSG00000259116	-1.269655687	0.037620819	AL049869.3
ENSG00000231171	-1.009320937	0.037659758	LINC01098
ENSG00000069424	-1.009320937	0.037659758	KCNAB2
ENSG00000232611	1.13876211	0.037689711	AL683813.1
ENSG00000100077	1.003127135	0.038290572	GRK3
ENSG00000270194	1.148662634	0.038387234	AC097359.2
ENSG00000230979	1.388245291	0.039238489	AC079250.1
ENSG00000205863	1.05536134	0.039576581	C1QTNF9B
ENSG00000210154	-1.079260462	0.039811039	MT-TD
ENSG00000101353	1.1120717	0.03991628	MROH8
ENSG00000143001	1.1120717	0.03991628	TMEM61
ENSG00000226015	-1.344926654	0.040101413	CCT8P1
ENSG00000217416	-1.344926654	0.040101413	ISCA1P1
ENSG00000236283	-1.344926654	0.040101413	AC019197.1
ENSG00000140022	-1.077156458	0.040564395	STON2
ENSG00000158882	-1.143304739	0.040756224	TOMM40L
ENSG00000258952	5.544445142	0.040964585	SALRNA1
ENSG00000228577	5.544445142	0.040964585	AC010731.2

ENSG00000259216	5.544445142	0.040964585	AC084757.1
ENSG00000213640	-5.688730633	0.040964585	EEF1DP4
ENSG00000277661	5.544445142	0.040964585	AL662791.2
ENSG00000274024	5.544445142	0.040964585	AL590282.1
ENSG00000269699	-5.688730633	0.040964585	ZIM2
ENSG00000212493	-5.688730633	0.040964585	SNORD19
ENSG00000228212	-5.688730633	0.040964585	OFD1P17
ENSG00000184486	-5.688730633	0.040964585	POU3F2
ENSG00000240751	-5.688730633	0.040964585	AC026348.1
ENSG00000271880	5.544445142	0.040964585	AGAP11
ENSG00000250432	-5.688730633	0.040964585	FAM242C
ENSG00000269444	5.544445142	0.040964585	AC011491.2
ENSG00000271989	-5.688730633	0.040964585	AL139424.1
ENSG00000238199	5.544445142	0.040964585	UBE2V2P3
ENSG00000229388	-5.688730633	0.040964585	LINC01715
ENSG00000273004	5.544445142	0.040964585	AL078644.1
ENSG00000272656	5.544445142	0.040964585	AC024933.1
ENSG00000250321	-5.688730633	0.040964585	AC079140.2
ENSG00000250483	5.544445142	0.040964585	PPM1AP1
ENSG00000283787	-5.688730633	0.040964585	PRR33
ENSG00000261775	-5.688730633	0.040964585	AC012435.2
ENSG00000267117	5.544445142	0.040964585	AC010525.1
ENSG00000187144	-5.688730633	0.040964585	SPATA21
ENSG00000234667	5.544445142	0.040964585	ACTBP13
ENSG00000188817	5.544445142	0.040964585	SNTN
ENSG00000272660	-5.688730633	0.040964585	AC090425.1
ENSG00000225981	5.544445142	0.040964585	AC102953.1
ENSG00000284606	-5.688730633	0.040964585	AC105233.5
ENSG00000259062	5.544445142	0.040964585	ACTN1-AS1
ENSG00000123700	-5.688730633	0.040964585	KCNJ2
ENSG00000274364	-5.688730633	0.040964585	AL110115.1
ENSG00000268536	5.544445142	0.040964585	AC005523.1
ENSG00000197380	5.544445142	0.040964585	DACT3
ENSG00000239670	-5.688730633	0.040964585	AL355864.2
ENSG00000224019	-5.688730633	0.040964585	RPL21P32
ENSG00000274997	5.544445142	0.040964585	HIST1H2AH
ENSG00000225885	5.544445142	0.040964585	AC023590.1
ENSG00000176236	5.544445142	0.040964585	C10orf111
ENSG00000234806	-5.688730633	0.040964585	RPL26P29
ENSG00000236756	-5.688730633	0.040964585	DNAJC9-AS1
ENSG00000121318	-5.688730633	0.040964585	TAS2R10
ENSG00000199477	5.544445142	0.040964585	SNORA31
ENSG00000280392	5.544445142	0.040964585	AC007496.3
ENSG00000261673	-5.688730633	0.040964585	AC009075.1
ENSG00000239246	5.544445142	0.040964585	AC008026.1
ENSG00000265204	5.544445142	0.040964585	AC090772.2
ENSG00000268442	-5.688730633	0.040964585	HAVCR1P1
ENSG00000204941	-5.688730633	0.040964585	PSG5
ENSG00000285530	-5.688730633	0.040964585	AL445928.2
ENSG00000200033	5.544445142	0.040964585	RNU6-403P
ENSG00000264157	-5.688730633	0.040964585	MIR3127
ENSG00000271778	5.544445142	0.040964585	AC080013.3
ENSG00000251175	5.544445142	0.040964585	AC008243.1
ENSG00000207497	5.544445142	0.040964585	RF00019
ENSG00000251221	-5.688730633	0.040964585	LINC01337
ENSG00000259869	-5.688730633	0.040964585	AL022344.2
ENSG00000264572	-5.688730633	0.040964585	MIR4296
ENSG00000278472	5.544445142	0.040964585	AC009268.2
ENSG00000183044	-5.688730633	0.040964585	ABAT
ENSG00000185710	-5.688730633	0.040964585	SMG1P4
ENSG00000264958	-5.688730633	0.040964585	ALOX12P1

ENSG00000279570	-5.688730633	0.040964585	AC099804.1
ENSG00000101342	-5.688730633	0.040964585	TLDC2
ENSG00000265407	5.544445142	0.040964585	MIR4324
ENSG00000254760	5.544445142	0.040964585	AC008750.1
ENSG00000284070	5.544445142	0.040964585	AP000356.2
ENSG00000234614	-5.688730633	0.040964585	AL450992.2
ENSG00000268154	-5.688730633	0.040964585	RF00017
ENSG00000198715	-5.688730633	0.040964585	GLMP
ENSG00000231536	5.544445142	0.040964585	AC226101.1
ENSG00000264354	-5.688730633	0.040964585	MIR3134
ENSG00000241219	5.544445142	0.040964585	AC078785.2
ENSG00000220581	-5.688730633	0.040964585	VN1R12P
ENSG00000217004	-5.688730633	0.040964585	Z97832.1
ENSG00000232491	-5.688730633	0.040964585	SAPCD2P3
ENSG00000170775	-5.688730633	0.040964585	GPR37
ENSG00000270988	5.544445142	0.040964585	AC019257.2
ENSG00000253923	5.544445142	0.040964585	AP002981.1
ENSG00000136881	-5.688730633	0.040964585	BAAT
ENSG00000119457	5.544445142	0.040964585	SLC46A2
ENSG00000188710	5.544445142	0.040964585	QRFP
ENSG00000215349	-5.688730633	0.040964585	MRPL3P1
ENSG00000100433	-5.688730633	0.040964585	KCNK10
ENSG00000259414	-5.688730633	0.040964585	AC242376.1
ENSG00000251760	-5.688730633	0.040964585	RNA5SP418
ENSG00000087250	5.544445142	0.040964585	MT3
ENSG00000161905	-5.688730633	0.040964585	ALOX15
ENSG00000279660	5.544445142	0.040964585	AC005703.6
ENSG00000263098	-5.688730633	0.040964585	AC068014.1
ENSG00000185198	5.544445142	0.040964585	PRSS57
ENSG00000252408	5.544445142	0.040964585	RF00212
ENSG00000213303	-5.688730633	0.040964585	AC008481.1
ENSG00000232401	5.544445142	0.040964585	LINC00112
ENSG00000131914	5.544445142	0.040964585	LIN28A
ENSG00000223583	5.544445142	0.040964585	AL513365.1
ENSG00000132698	5.544445142	0.040964585	RAB25
ENSG00000213729	5.544445142	0.040964585	AC098828.1
ENSG00000226185	5.544445142	0.040964585	TRIM64FP
ENSG00000163815	5.544445142	0.040964585	CLEC3B
ENSG00000164161	5.544445142	0.040964585	HHIP
ENSG00000273294	5.544445142	0.040964585	C1QTNF3-AMACR
ENSG00000249664	5.544445142	0.040964585	AC027338.1
ENSG00000249791	-5.688730633	0.040964585	AC008494.2
ENSG00000113721	-5.688730633	0.040964585	PDGFRB
ENSG00000217862	5.544445142	0.040964585	HIST1H4PS1
ENSG00000236475	5.544445142	0.040964585	TRIM26BP
ENSG00000226291	5.544445142	0.040964585	AC091729.2
ENSG00000149970	-5.688730633	0.040964585	CNKSR2
ENSG00000168263	-5.688730633	0.040964585	KCNV2
ENSG00000239593	5.544445142	0.040964585	AL513122.2
ENSG00000230911	-5.688730633	0.040964585	PPIHP1
ENSG00000254842	5.544445142	0.040964585	LINC02551
ENSG00000165449	-5.688730633	0.040964585	SLC16A9
ENSG00000257221	-5.688730633	0.040964585	AC007569.1
ENSG00000277595	-5.688730633	0.040964585	AC007546.1
ENSG00000267868	5.544445142	0.040964585	AL356740.1
ENSG00000212302	5.544445142	0.040964585	RF00588
ENSG00000259251	-5.688730633	0.040964585	AC104590.1
ENSG00000206168	-5.688730633	0.040964585	Z69890.1
ENSG00000275371	-5.688730633	0.040964585	AC012645.4
ENSG00000279841	5.544445142	0.040964585	AC092135.3
ENSG00000262358	-5.688730633	0.040964585	AC116914.1

ENSG00000206859	-5.688730633	0.040964585	RNU6-767P
ENSG00000263860	5.544445142	0.040964585	AC011840.3
ENSG00000141748	-5.688730633	0.040964585	ARL5C
ENSG00000198336	-5.688730633	0.040964585	MYL4
ENSG00000213246	-5.688730633	0.040964585	SUPT4H1
ENSG00000172794	-5.688730633	0.040964585	RAB37
ENSG00000262870	5.544445142	0.040964585	CYCSP40
ENSG00000267808	5.544445142	0.040964585	AC018755.1
ENSG00000252098	-5.688730633	0.040964585	RF00019
ENSG00000278966	-5.688730633	0.040964585	AL031602.1
ENSG00000227278	-5.688730633	0.040964585	AL603839.1
ENSG00000134240	-5.688730633	0.040964585	HMGCS2
ENSG00000143631	-5.688730633	0.040964585	FLG
ENSG00000200755	5.544445142	0.040964585	RNA5SP68
ENSG00000285638	-5.688730633	0.040964585	AL138927.1
ENSG00000134365	-5.688730633	0.040964585	CFHR4
ENSG00000212205	5.544445142	0.040964585	RF00019
ENSG00000225449	5.544445142	0.040964585	RAB6C-AS1
ENSG00000186825	5.544445142	0.040964585	C2orf27B
ENSG00000231903	-5.688730633	0.040964585	AC079354.3
ENSG00000228513	5.544445142	0.040964585	AC023271.1
ENSG00000227479	-5.688730633	0.040964585	AC124861.1
ENSG00000202071	-5.688730633	0.040964585	RF00019
ENSG00000239440	-5.688730633	0.040964585	LINC02008
ENSG00000207002	-5.688730633	0.040964585	RF00392
ENSG00000251129	-5.688730633	0.040964585	LINC02506
ENSG00000169248	-5.688730633	0.040964585	CXCL11
ENSG00000246876	-5.688730633	0.040964585	LINC02466
ENSG00000279481	5.544445142	0.040964585	AC104791.2
ENSG00000249012	5.544445142	0.040964585	AC104819.1
ENSG00000248429	5.544445142	0.040964585	FAM198B-AS1
ENSG00000249500	-5.688730633	0.040964585	LINC01179
ENSG00000249650	-5.688730633	0.040964585	AC106772.1
ENSG00000040731	-5.688730633	0.040964585	CDH10
ENSG00000112964	-5.688730633	0.040964585	GHR
ENSG00000250889	-5.688730633	0.040964585	LINC01336
ENSG00000255647	5.544445142	0.040964585	AC093510.1
ENSG00000250551	-5.688730633	0.040964585	MIR583HG
ENSG00000253798	-5.688730633	0.040964585	AC008694.1
ENSG00000204287	-5.688730633	0.040964585	HLA-DRA
ENSG00000223169	5.544445142	0.040964585	RNA5SP209
ENSG00000236166	5.544445142	0.040964585	AL021408.1
ENSG00000218776	-5.688730633	0.040964585	MTATP6P31
ENSG00000272537	-5.688730633	0.040964585	AC005014.3
ENSG00000242258	-5.688730633	0.040964585	LINC00996
ENSG00000171433	5.544445142	0.040964585	GLOD5
ENSG00000228125	-5.688730633	0.040964585	AKIRIN1P2
ENSG00000207359	5.544445142	0.040964585	RNU6-925P
ENSG00000261678	5.544445142	0.040964585	SCRT1
ENSG00000251733	-5.688730633	0.040964585	SCARNA8
ENSG00000224648	5.544445142	0.040964585	LINC01627
ENSG00000284116	5.544445142	0.040964585	AL772307.1
ENSG00000207563	5.544445142	0.040964585	MIR23B
ENSG00000234502	5.544445142	0.040964585	FYTTD1P1
ENSG00000070985	5.544445142	0.040964585	TRPM5
ENSG00000166840	-5.688730633	0.040964585	GLYATL1
ENSG00000255118	5.544445142	0.040964585	AP003306.2
ENSG00000132744	-5.688730633	0.040964585	ACY3
ENSG00000202522	-5.688730633	0.040964585	RF00019
ENSG00000254459	-5.688730633	0.040964585	AP002812.2
ENSG00000204397	-5.688730633	0.040964585	CARD16

ENSG00000254854	-5.688730633	0.040964585	AP003390.1
ENSG00000260209	5.544445142	0.040964585	AP000842.3
ENSG00000150051	5.544445142	0.040964585	MKX
ENSG00000226163	-5.688730633	0.040964585	AL513185.1
ENSG00000107831	5.544445142	0.040964585	FGF8
ENSG00000242912	-5.688730633	0.040964585	RN7SL384P
ENSG00000274659	5.544445142	0.040964585	LINC02371
ENSG00000048540	-5.688730633	0.040964585	LMO3
ENSG00000255652	-5.688730633	0.040964585	AC140847.2
ENSG00000257925	5.544445142	0.040964585	AC008083.1
ENSG00000257951	5.544445142	0.040964585	AC126177.6
ENSG00000230641	5.544445142	0.040964585	USP12-AS2
ENSG00000279149	-5.688730633	0.040964585	AL356750.1
ENSG00000180138	5.544445142	0.040964585	CSNK1A1L
ENSG00000120658	-5.688730633	0.040964585	ENOX1
ENSG00000236577	-5.688730633	0.040964585	SNRPGP14
ENSG00000223404	-5.688730633	0.040964585	LINC00397
ENSG00000279237	5.544445142	0.040964585	AL161773.1
ENSG00000187483	5.544445142	0.040964585	SERPINA13P
ENSG00000202542	-5.688730633	0.040964585	RF00019
ENSG00000259228	-5.688730633	0.040964585	HNRNPA1P62
ENSG00000182397	-5.688730633	0.040964585	DNM1P46
ENSG00000261442	-5.688730633	0.040964585	AC023830.1
ENSG00000177238	5.544445142	0.040964585	TRIM72
ENSG00000252624	-5.688730633	0.040964585	RNA5SP409
ENSG00000252673	-5.688730633	0.040964585	RNA5SP421
ENSG00000260865	5.544445142	0.040964585	AC010287.1
ENSG00000260185	5.544445142	0.040964585	AC009097.1
ENSG00000237328	-5.688730633	0.040964585	RAI1-AS1
ENSG00000228000	-5.688730633	0.040964585	RPL7AP65
ENSG00000259928	5.544445142	0.040964585	AC011840.2
ENSG00000231870	5.544445142	0.040964585	KRT17P3
ENSG00000264196	5.544445142	0.040964585	AC011120.1
ENSG00000167851	-5.688730633	0.040964585	CD300A
ENSG00000267624	-5.688730633	0.040964585	AC087645.1
ENSG00000252034	5.544445142	0.040964585	RNY4P37
ENSG00000266905	5.544445142	0.040964585	AC114684.1
ENSG00000235065	5.544445142	0.040964585	RPL24P2
ENSG00000207053	-5.688730633	0.040964585	RNU6-937P
ENSG00000249840	5.544445142	0.040964585	GAPDHP76
ENSG00000241464	-5.688730633	0.040964585	RPL39P38
ENSG00000273189	-5.688730633	0.040964585	AC010619.2
ENSG00000233471	5.544445142	0.040964585	KRT18P62
ENSG00000100336	-5.688730633	0.040964585	APOL4
ENSG00000273096	5.544445142	0.040964585	AL021707.8
ENSG00000159307	-5.688730633	0.040964585	SCUBE1
ENSG00000226954	5.544445142	0.040964585	AL023802.1
ENSG00000280191	-5.688730633	0.040964585	LINC01669
ENSG00000249493	-5.688730633	0.040964585	ANKRD20A18P
ENSG00000278867	1.426964524	0.040993521	AC090616.6
ENSG00000271133	1.426964524	0.040993521	AC004130.1
ENSG00000248740	-1.360071702	0.040993521	LINC02428
ENSG00000261594	-1.360071702	0.040993521	TPBGL
ENSG00000186051	2.370254356	0.041032031	TAL2
ENSG00000228201	2.370254356	0.041032031	AL022341.1
ENSG00000271893	2.370254356	0.041032031	AC064834.1
ENSG00000043039	2.370254356	0.041032031	BARX2
ENSG00000099957	2.370254356	0.041032031	P2RX6
ENSG00000254873	2.370254356	0.041032031	AP001267.1
ENSG00000186642	2.246143093	0.041032031	PDE2A
ENSG00000122735	-2.530410598	0.041032031	DNAI1

ENSG00000213706	2.246143093	0.041032031	AL590762.2
ENSG00000275017	-2.530410598	0.041032031	AL353748.2
ENSG00000149212	2.370254356	0.041032031	SESN3
ENSG00000257475	2.246143093	0.041032031	AC068888.2
ENSG00000274765	-2.530410598	0.041032031	AC018926.1
ENSG00000263674	2.370254356	0.041032031	AC026620.1
ENSG00000285646	2.370254356	0.041032031	AL021155.2
ENSG00000250410	2.246143093	0.041032031	AC112722.1
ENSG00000255303	2.246143093	0.041032031	OR5BA1P
ENSG00000165805	2.246143093	0.041032031	C12orf50
ENSG00000272525	2.246143093	0.041032031	AC099522.2
ENSG00000237301	-2.530410598	0.041032031	AL121992.1
ENSG00000183873	-2.530410598	0.041032031	SCN5A
ENSG00000264520	2.246143093	0.041032031	AC005154.4
ENSG00000280149	2.246143093	0.041032031	AC004877.2
ENSG00000104055	2.370254356	0.041032031	TGM5
ENSG00000226653	-2.530410598	0.041032031	OR13Z1P
ENSG00000243819	2.246143093	0.041032031	RN7SL832P
ENSG00000254503	-2.530410598	0.041032031	AC010319.1
ENSG00000242866	-2.530410598	0.041032031	STRC
ENSG00000232750	2.370254356	0.041032031	AL139132.1
ENSG00000236393	2.370254356	0.041032031	AC091806.1
ENSG00000253223	-2.530410598	0.041032031	AC110998.1
ENSG00000276032	2.370254356	0.041032031	AL627230.4
ENSG00000258443	2.246143093	0.041032031	AC005225.1
ENSG00000162614	2.246143093	0.041032031	NEXN
ENSG00000270716	2.246143093	0.041032031	BNIP3P15
ENSG00000272834	-2.530410598	0.041032031	AL022238.3
ENSG00000215244	-1.215620343	0.041535125	AL137145.2
ENSG00000160145	-1.215620343	0.041535125	KALRN
ENSG00000188859	1.132463051	0.041652925	FAM78B
ENSG00000225032	1.072386292	0.041706837	AL162586.1
ENSG00000285533	1.02875402	0.041897242	AP001362.2
ENSG00000272518	-1.377735865	0.041903977	AC036214.2
ENSG00000153093	1.478003147	0.041903977	ACOXL
ENSG00000007952	1.478003147	0.041903977	NOX1
ENSG00000258940	1.478003147	0.041903977	AL132639.2
ENSG00000254634	1.478003147	0.041903977	SMG1P6
ENSG00000275880	1.238877576	0.042571852	AL139385.1
ENSG00000108947	-1.219296489	0.042571852	EFNB3
ENSG00000218418	1.143914578	0.042612006	AL591135.1
ENSG00000180176	1.539187011	0.042814164	TH
ENSG00000259287	1.539187011	0.042814164	AC010809.1
ENSG00000175003	-1.3986064	0.042814164	SLC22A1
ENSG00000182057	1.539187011	0.042814164	OGFRP1
ENSG00000138080	1.065559851	0.04334011	EMILIN1
ENSG00000142347	-1.173735165	0.043639812	MYO1F
ENSG00000271895	-1.223390766	0.043684225	AL109811.3
ENSG00000110900	-1.223390766	0.043684225	TSPAN11
ENSG00000246339	-1.223390766	0.043684225	EXTL3-AS1
ENSG00000253837	2.032631229	0.043991311	AC090197.1
ENSG00000106336	2.032631229	0.043991311	FBXO24
ENSG00000213901	2.032631229	0.043991311	SLC23A3
ENSG00000137843	2.032631229	0.043991311	PAK6
ENSG00000186105	-2.099646447	0.043991311	LRRC70
ENSG00000229298	2.032631229	0.043991311	TUBB8P1
ENSG00000095587	-2.099646447	0.043991311	TLL2
ENSG00000132965	-2.099646447	0.043991311	ALOX5AP
ENSG00000269653	-2.099646447	0.043991311	AC011479.3
ENSG00000217644	2.032631229	0.043991311	AL355864.1
ENSG00000217385	2.032631229	0.043991311	PSMC1P11

ENSG00000215347	2.032631229	0.043991311	SLC25A5P1
ENSG00000177096	2.032631229	0.043991311	PHETA2
ENSG00000213694	-2.099646447	0.043991311	S1PR3
ENSG00000173401	-2.099646447	0.043991311	GLIPR1L1
ENSG00000274307	2.032631229	0.043991311	AC023449.2
ENSG00000144837	-2.099646447	0.043991311	PLA1A
ENSG00000254477	-2.099646447	0.043991311	AP000640.1
ENSG00000241889	-2.099646447	0.043991311	AC079944.2
ENSG00000146857	-2.099646447	0.043991311	STRA8
ENSG00000267257	-2.099646447	0.043991311	AC105105.1
ENSG00000235036	1.489133872	0.044489089	AL035456.1
ENSG00000104826	1.489133872	0.044489089	LHB
ENSG00000108176	-1.643568238	0.044489089	DNAJC12
ENSG00000248923	-1.643568238	0.044489089	MTND5P11
ENSG00000095932	-1.643568238	0.044489089	SMIM24
ENSG00000231154	1.140824157	0.044743519	MORF4L2-AS1
ENSG00000173421	-1.227978833	0.044879015	CCDC36
ENSG00000259953	-1.227978833	0.044879015	AL138756.1
ENSG00000272473	1.570877631	0.045117465	AC006273.1
ENSG00000274737	1.570877631	0.045117465	AC004466.2
ENSG00000187010	1.570877631	0.045117465	RHD
ENSG00000213790	1.570877631	0.045117465	OLA1P1
ENSG00000206113	1.570877631	0.045117465	CFAP99
ENSG00000284681	-1.71047962	0.045117465	AC007240.1
ENSG00000106003	-1.71047962	0.045117465	LFNG
ENSG00000267416	-1.71047962	0.045117465	AC025048.4
ENSG00000260796	-1.71047962	0.045117465	AC145285.3
ENSG00000132330	1.079088332	0.045180411	SCLY
ENSG00000088827	1.822346074	0.045202178	SIGLEC1
ENSG00000279880	-1.920194328	0.045202178	AC134407.3
ENSG00000255933	1.822346074	0.045202178	AC117500.2
ENSG00000231050	1.822346074	0.045202178	AL109917.1
ENSG00000224897	1.822346074	0.045202178	POT1-AS1
ENSG00000273271	-1.920194328	0.045202178	AP000254.1
ENSG00000229447	-1.920194328	0.045202178	AC114495.2
ENSG00000102924	-1.920194328	0.045202178	CBLN1
ENSG00000143217	1.822346074	0.045202178	NECTIN4
ENSG00000275393	-1.920194328	0.045202178	AC018695.6
ENSG00000265625	1.822346074	0.045202178	AC104564.5
ENSG00000265752	1.822346074	0.045202178	AC010754.1
ENSG00000170044	-1.920194328	0.045202178	ZPLD1
ENSG00000128254	1.822346074	0.045202178	C22orf24
ENSG00000271797	-1.920194328	0.045202178	AC008494.3
ENSG00000187122	1.822346074	0.045202178	SLIT1
ENSG00000162738	-1.920194328	0.045202178	VANGL2
ENSG00000258957	-1.920194328	0.045202178	AL359317.2
ENSG00000242268	1.677371434	0.045435959	LINC02082
ENSG00000279063	1.677371434	0.045435959	AC008735.5
ENSG00000144712	1.677371434	0.045435959	CAND2
ENSG00000250081	1.677371434	0.045435959	AC025176.1
ENSG00000181408	-1.798599993	0.045435959	UTS2R
ENSG00000183150	1.677371434	0.045435959	GPR19
ENSG00000109743	1.677371434	0.045435959	BST1
ENSG00000263818	-1.798599993	0.045435959	RDM1P5
ENSG00000118307	1.677371434	0.045435959	CASC1
ENSG00000285077	-1.798599993	0.045435959	ARHGAP11B
ENSG00000178404	1.154420252	0.045931264	CEP295NL
ENSG00000280913	1.314346906	0.046163285	CTSLP2
ENSG00000104889	1.314346906	0.046163285	RNASEH2A
ENSG00000147255	1.086520092	0.046190383	IGSF1
ENSG00000281195	-1.115354747	0.046223025	AC007878.1

ENSG00000160471	1.16936851	0.047212297	COX6B2
ENSG00000267102	1.16936851	0.047212297	AC060766.1
ENSG00000102575	-1.047920031	0.047267958	ACP5
ENSG00000250616	1.106529235	0.047409691	AC012645.1
ENSG00000234028	1.106529235	0.047409691	AC062029.1
ENSG00000284693	1.346583783	0.04754413	AL928921.2
ENSG00000110328	1.346583783	0.04754413	GALNT18
ENSG00000267395	-1.286165205	0.04754413	DM1-AS
ENSG00000210164	-1.012797116	0.04760398	MT-TG
ENSG00000102290	1.185882136	0.048597149	PCDH11X
ENSG00000279520	-1.179203843	0.048597149	AC093525.8
ENSG00000103269	-1.179203843	0.048597149	RHBDL1
ENSG00000279148	1.089601456	0.048686192	AC126474.1
ENSG00000179397	1.338102182	0.049028188	CATSPERE
ENSG00000269974	-1.296212977	0.049028188	AC091057.4
ENSG00000238273	-1.296212977	0.049028188	AC108058.1
ENSG00000232713	-1.296212977	0.049028188	AC010733.1
ENSG00000158571	1.338102182	0.049028188	PFKFB1
ENSG00000280303	1.338102182	0.049028188	ERICD
ENSG00000267419	-1.296212977	0.049028188	AC011477.1
ENSG00000215417	-1.040840144	0.049653713	MIR17HG

Table S5. Differentially expressed genes in *NNMT* KD SKOV3 cells identified by RNA-seq analysis.

gene_id	log2FoldChange	pvalue	gene_name
ENSG00000166741	-3.856526581	1.10E-16	NNMT
ENSG00000097021	-2.671207016	1.51E-09	ACOT7
ENSG00000224858	3.101602691	4.93E-09	RPL29P11
ENSG00000242779	2.527348444	1.88E-07	ZNF702P
ENSG00000107833	-2.55818291	3.24E-07	NPM3
ENSG00000111275	-2.252815462	1.24E-06	ALDH2
ENSG00000142507	-2.03975504	2.92E-06	PSMB6
ENSG00000112299	-2.183928931	3.68E-06	VNN1
ENSG00000110848	-2.979500505	9.35E-06	CD69
ENSG00000115009	-2.230982637	9.51E-06	CCL20
ENSG00000230387	3.935229984	1.06E-05	AL118508.1
ENSG00000167460	1.855608533	1.20E-05	TPM4
ENSG00000115226	-1.917315733	2.92E-05	FNDC4
ENSG00000128422	1.994320446	3.94E-05	KRT17
ENSG00000125753	-1.737501005	4.94E-05	VASP
ENSG00000172348	-2.190865411	6.24E-05	RCAN2
ENSG00000081041	-1.726547808	7.17E-05	CXCL2
ENSG00000224063	4.335057086	7.35E-05	AC007319.1
ENSG00000216906	7.166057172	7.55E-05	AL355312.1
ENSG00000183111	-1.71106764	9.67E-05	ARHGEF37
ENSG00000171631	1.755454743	9.86E-05	P2RY6
ENSG00000125538	-1.6587636	1.02E-04	IL1B
ENSG00000023445	-1.658512896	1.08E-04	BIRC3
ENSG00000100033	3.241618727	1.12E-04	PRODH
ENSG00000119899	-1.681752276	1.17E-04	SLC17A5
ENSG00000271826	-7.112014528	1.20E-04	PLS3-AS1
ENSG00000160200	-1.717875691	1.49E-04	CBS
ENSG00000280231	4.204562234	1.64E-04	AL031719.2
ENSG00000090339	-2.247316327	1.65E-04	ICAM1
ENSG00000114745	-1.640672674	1.69E-04	GORASP1
ENSG00000112303	-1.980585708	1.70E-04	VNN2
ENSG00000163283	-1.699687485	1.83E-04	ALPP
ENSG00000179362	-2.591460632	1.93E-04	HMGN2P46
ENSG00000168386	6.997387326	1.94E-04	FILIP1L
ENSG00000105186	-1.574445834	2.35E-04	ANKRD27
ENSG00000152689	2.501308947	2.46E-04	RASGRP3
ENSG00000134339	-1.831946502	2.52E-04	SAA2
ENSG00000159958	-2.943160185	2.52E-04	TNFRSF13C
ENSG00000092969	2.413378581	2.58E-04	TGFB2
ENSG00000110651	-1.532936822	2.68E-04	CD81
ENSG00000160188	3.090287056	3.00E-04	RSPH1
ENSG00000248498	2.840944836	3.42E-04	ASNSP1
ENSG00000267370	3.416785766	3.49E-04	AC008752.3
ENSG00000100292	1.512446864	3.53E-04	HMOX1
ENSG00000273010	4.061079313	3.77E-04	AL360270.3
ENSG00000184371	-1.48659645	3.97E-04	CSF1
ENSG00000174827	2.346460875	4.12E-04	PDZK1
ENSG00000172893	-1.484776138	4.17E-04	DHCR7
ENSG00000118503	-1.491910988	4.38E-04	TNFAIP3
ENSG00000134198	1.585733383	4.67E-04	TSPAN2
ENSG00000206075	1.560163119	4.85E-04	SERPINB5
ENSG00000242419	3.353013279	5.07E-04	PCDHGC4
ENSG00000130477	-1.769680551	5.07E-04	UNC13A
ENSG00000145107	1.986014532	5.44E-04	TM4SF19
ENSG00000198948	-1.459492696	5.53E-04	MFAP3L
ENSG00000249740	3.983605614	5.75E-04	OSMR-AS1
ENSG00000226179	3.983605614	5.75E-04	LINC00685
ENSG00000162591	-1.463631369	5.78E-04	MEGF6

ENSG00000172594	-1.994721006	6.62E-04	SMPDL3A
ENSG00000181029	2.450351377	6.70E-04	TRAPPC5
ENSG00000176387	-1.765539785	7.11E-04	HSD11B2
ENSG00000163661	-1.693605501	7.15E-04	PTX3
ENSG00000136514	-2.103898765	7.33E-04	RTP4
ENSG00000105929	3.286290914	7.40E-04	ATP6V0A4
ENSG00000232859	2.08514177	7.59E-04	LYRM9
ENSG00000161921	-1.547130601	8.42E-04	CXCL16
ENSG00000283408	-6.72819678	8.58E-04	MIR548A1HG
ENSG00000233231	6.700430421	8.58E-04	HNRNPA1P49
ENSG00000254634	6.700430421	8.58E-04	SMG1P6
ENSG00000003056	-1.428115562	8.59E-04	M6PR
ENSG00000232450	2.527704054	8.84E-04	AL133517.1
ENSG00000152128	2.019691707	8.91E-04	TMEM163
ENSG00000157111	-1.490041975	9.18E-04	TMEM171
ENSG00000260287	2.095853791	9.74E-04	TBC1D3G
ENSG00000138347	1.527936564	1.02E-03	MYPN
ENSG00000104951	-1.448335706	1.05E-03	IL4I1
ENSG00000144230	2.202525889	1.07E-03	GPR17
ENSG00000145623	-1.376888513	1.07E-03	OSMR
ENSG00000087494	1.83930465	1.11E-03	PTHLH
ENSG00000102098	-1.473759913	1.17E-03	SCML2
ENSG00000268751	2.860128456	1.17E-03	SCGB1B2P
ENSG00000146535	-1.36520446	1.23E-03	GNA12
ENSG00000049249	-1.588764036	1.27E-03	TNFRSF9
ENSG00000104081	-1.52669009	1.35E-03	BMF
ENSG00000270757	3.814935768	1.37E-03	HSPE1-MOB4
ENSG00000231887	3.814935768	1.37E-03	PRH1
ENSG00000155189	-1.344787212	1.39E-03	AGPAT5
ENSG00000243766	-6.613853094	1.44E-03	HOTTIP
ENSG00000254703	6.586108754	1.44E-03	SENCR
ENSG00000279901	6.586108754	1.44E-03	AC092117.2
ENSG00000278341	-6.613853094	1.44E-03	AC138028.6
ENSG00000255121	1.680755602	1.52E-03	AP003392.4
ENSG00000166387	-1.657623045	1.55E-03	PPFIBP2
ENSG00000106244	-1.337517789	1.57E-03	PDAP1
ENSG00000204556	-2.483858407	1.59E-03	AL450124.1
ENSG00000141580	-1.325081018	1.60E-03	WDR45B
ENSG00000250033	1.930656219	1.60E-03	SLC7A11-AS1
ENSG00000233024	1.876275808	1.81E-03	AC126755.2
ENSG00000158747	-1.306903236	1.83E-03	NBL1
ENSG00000172936	-1.41612367	1.84E-03	MYD88
ENSG00000069702	-1.419055935	1.86E-03	TGFBR3
ENSG00000230409	1.457234639	1.93E-03	TCEA1P2
ENSG00000103044	-1.346520834	1.95E-03	HAS3
ENSG00000126216	-1.3318134	1.96E-03	TUBGCP3
ENSG00000165323	2.270490389	2.00E-03	FAT3
ENSG00000157680	1.749284485	2.06E-03	DGKI
ENSG00000166707	3.722578933	2.13E-03	ZCCHC18
ENSG00000237575	3.722578933	2.13E-03	PYY2
ENSG00000101017	-1.314638686	2.17E-03	CD40
ENSG00000173432	-1.32483589	2.18E-03	SAA1
ENSG00000205583	-1.415842485	2.20E-03	STAG3L1
ENSG00000253159	2.520527001	2.28E-03	PCDHGA12
ENSG00000274849	2.520527001	2.28E-03	AC023043.4
ENSG00000231999	-3.119558668	2.36E-03	LRRC8C-DT
ENSG00000143603	3.065334294	2.36E-03	KCNN3
ENSG00000187479	3.065334294	2.36E-03	C11orf96
ENSG00000275198	3.065334294	2.36E-03	AL512791.2
ENSG00000260618	3.065334294	2.36E-03	AC025917.1
ENSG00000263624	3.065334294	2.36E-03	AC055811.1

ENSG00000279762	3.065334294	2.36E-03	AC005899.8
ENSG00000279133	-3.119558668	2.36E-03	AC018628.1
ENSG00000105711	-1.330888736	2.37E-03	SCN1B
ENSG00000169429	-1.271713911	2.37E-03	CXCL8
ENSG00000166578	-1.304553479	2.40E-03	IQCD
ENSG00000139874	1.72587356	2.41E-03	SSTR1
ENSG00000116990	-1.801300493	2.42E-03	MYCL
ENSG00000170801	6.46194233	2.43E-03	HTRA3
ENSG00000248308	6.46194233	2.43E-03	AC138938.1
ENSG00000049540	-6.489660693	2.43E-03	ELN
ENSG00000270427	6.46194233	2.43E-03	NRBF2P5
ENSG00000188649	6.46194233	2.43E-03	CC2D2B
ENSG00000012779	-1.296694033	2.44E-03	ALOX5
ENSG00000183317	-1.308025761	2.59E-03	EPHA10
ENSG00000279821	1.588877011	2.64E-03	AC145098.2
ENSG00000177990	2.042622984	2.82E-03	DPY19L2
ENSG00000210112	2.042622984	2.82E-03	MT-TM
ENSG00000156973	-1.302696263	2.84E-03	PDE6D
ENSG00000263257	1.922353339	2.89E-03	AC040173.1
ENSG00000197385	1.770332912	2.90E-03	ZNF860
ENSG00000204682	1.335056084	3.01E-03	CASC10
ENSG00000100522	-1.273085568	3.03E-03	GNPNAT1
ENSG00000171236	-1.469430436	3.14E-03	LRG1
ENSG00000272543	2.459454565	3.15E-03	MIR4787
ENSG00000160111	-1.285958667	3.17E-03	CPAMD8
ENSG00000188681	-1.914043597	3.23E-03	TEKT4P2
ENSG00000007001	3.623902858	3.35E-03	UPP2
ENSG00000128710	-3.676606734	3.35E-03	HOXD10
ENSG00000224424	-3.676606734	3.35E-03	PRKAR2A-AS1
ENSG00000128564	3.623902858	3.35E-03	VGF
ENSG00000236908	-3.676606734	3.35E-03	AC005865.1
ENSG00000167772	1.291317495	3.45E-03	ANGPTL4
ENSG00000197332	2.171386379	3.49E-03	AC008543.1
ENSG00000113578	2.983462931	3.52E-03	FGF1
ENSG00000129007	2.983462931	3.52E-03	CALML4
ENSG00000261770	-3.037676016	3.52E-03	AC006504.1
ENSG00000121104	-1.321812432	3.55E-03	FAM117A
ENSG00000257365	-1.331597436	3.57E-03	FNTB
ENSG00000073670	-1.484218535	3.60E-03	ADAM11
ENSG00000108797	-1.270379434	3.63E-03	CNTNAP1
ENSG00000091879	-1.215513704	3.78E-03	ANGPT2
ENSG00000154874	1.572542915	3.78E-03	CCDC144B
ENSG00000163874	-1.206105951	3.87E-03	ZC3H12A
ENSG00000197594	-1.305181735	4.06E-03	ENPP1
ENSG00000274386	-2.107728619	4.07E-03	TMEM269
ENSG00000148677	2.052270065	4.07E-03	ANKRD1
ENSG00000277053	-1.236152835	4.16E-03	GTF2IP1
ENSG00000173662	6.326074454	4.17E-03	TAS1R1
ENSG00000118292	6.326074454	4.17E-03	C1orf54
ENSG00000279897	6.326074454	4.17E-03	BIRC6-AS2
ENSG00000240602	6.326074454	4.17E-03	AADACP1
ENSG00000285865	6.326074454	4.17E-03	AC010285.3
ENSG00000250483	-6.353761709	4.17E-03	PPM1AP1
ENSG00000230445	-6.353761709	4.17E-03	LRRC37A6P
ENSG00000121318	6.326074454	4.17E-03	TAS2R10
ENSG00000257175	-6.353761709	4.17E-03	CR383656.1
ENSG00000259000	6.326074454	4.17E-03	DOCK11P1
ENSG00000270705	-6.353761709	4.17E-03	AL161669.2
ENSG00000264175	-6.353761709	4.17E-03	MIR3189
ENSG00000136167	1.225365938	4.32E-03	LCP1
ENSG00000228626	2.395682078	4.37E-03	AC245100.3

ENSG00000163131	-1.206506769	4.40E-03	CTSS
ENSG00000204611	1.284061799	4.42E-03	ZNF616
ENSG00000163071	1.671640436	4.47E-03	SPATA18
ENSG00000117122	1.846656025	4.50E-03	MFAP2
ENSG00000188916	1.846656025	4.50E-03	INSYN2
ENSG00000082074	1.574012505	4.53E-03	FYB1
ENSG00000214145	2.119158717	4.62E-03	LINC00887
ENSG00000108342	-2.174524113	4.62E-03	CSF3
ENSG00000123610	-2.640912835	4.85E-03	TNFAIP6
ENSG00000182162	-2.640912835	4.85E-03	P2RY8
ENSG00000179583	-1.342427726	4.95E-03	CIITA
ENSG00000145685	-1.186928281	5.00E-03	LHFPL2
ENSG00000230882	-1.444980631	5.03E-03	AC005077.4
ENSG00000135677	-1.169032569	5.04E-03	GNS
ENSG00000168542	1.393556612	5.07E-03	COL3A1
ENSG00000103257	-1.164788573	5.09E-03	SLC7A5
ENSG00000087586	-1.177932964	5.23E-03	AURKA
ENSG00000273002	2.896664448	5.25E-03	AL355388.2
ENSG00000131730	2.896664448	5.25E-03	CKMT2
ENSG00000166349	2.896664448	5.25E-03	RAG1
ENSG00000148735	-2.950864843	5.25E-03	PLEKHS1
ENSG00000272173	2.896664448	5.25E-03	U47924.2
ENSG00000237515	2.896664448	5.25E-03	SHISA9
ENSG00000236782	-3.570663838	5.28E-03	AL391650.1
ENSG00000228035	3.517978863	5.28E-03	NGF-AS1
ENSG00000228238	3.517978863	5.28E-03	AL096803.1
ENSG00000183281	-3.570663838	5.28E-03	PLGLB1
ENSG00000222014	-3.570663838	5.28E-03	RAB6C
ENSG00000226055	3.517978863	5.28E-03	PAICSP1
ENSG00000225614	3.517978863	5.28E-03	ZNF469
ENSG00000270264	3.517978863	5.28E-03	NDUFB8P2
ENSG00000152292	2.207286219	5.29E-03	SH2D6
ENSG00000214652	2.207286219	5.29E-03	ZNF727
ENSG00000152137	1.602757093	5.30E-03	HSPB8
ENSG00000125398	1.734184703	5.31E-03	SOX9
ENSG00000049130	1.24363215	5.49E-03	KITLG
ENSG00000258711	1.608341446	5.64E-03	AL358334.2
ENSG00000132128	-1.233590009	5.79E-03	LRRC41
ENSG00000179909	1.909872724	5.91E-03	ZNF154
ENSG00000155959	-1.17272253	5.99E-03	VBP1
ENSG00000248027	2.328959713	6.07E-03	AP001351.1
ENSG00000049089	-1.887812442	6.49E-03	COL9A2
ENSG00000196547	-1.142982338	6.51E-03	MAN2A2
ENSG00000242242	1.394777171	6.53E-03	NECTIN3-AS1
ENSG00000280287	1.324568859	6.73E-03	AC131212.3
ENSG00000008311	-2.008616338	6.89E-03	AASS
ENSG00000135929	2.508676984	6.97E-03	CYP27A1
ENSG00000227954	2.508676984	6.97E-03	TARID
ENSG00000268350	-2.563429028	6.97E-03	FAM156A
ENSG00000136573	2.508676984	6.97E-03	BLK
ENSG00000272141	-6.203717299	7.24E-03	AL390719.2
ENSG00000260063	6.176067971	7.24E-03	AL512408.1
ENSG00000283973	6.176067971	7.24E-03	AC099795.1
ENSG00000270110	6.176067971	7.24E-03	AL353593.3
ENSG00000260698	-6.203717299	7.24E-03	AL591848.3
ENSG00000242136	6.176067971	7.24E-03	AC093904.2
ENSG00000163421	6.176067971	7.24E-03	PROK2
ENSG00000240057	6.176067971	7.24E-03	AC078785.1
ENSG00000272989	6.176067971	7.24E-03	LINC02012
ENSG00000248449	6.176067971	7.24E-03	PCDHGB8P
ENSG00000225945	6.176067971	7.24E-03	AL121574.1

ENSG00000207588	6.176067971	7.24E-03	MIR593
ENSG00000279347	6.176067971	7.24E-03	AC021945.1
ENSG00000176029	6.176067971	7.24E-03	C11orf16
ENSG00000200485	6.176067971	7.24E-03	RF00019
ENSG00000225418	6.176067971	7.24E-03	AKR1C5P
ENSG00000151303	6.176067971	7.24E-03	AL136982.1
ENSG00000139915	6.176067971	7.24E-03	MDGA2
ENSG00000137868	-6.203717299	7.24E-03	STRA6
ENSG00000186481	6.176067971	7.24E-03	ANKRD20A5P
ENSG00000130173	-6.203717299	7.24E-03	ANGPTL8
ENSG00000099977	6.176067971	7.24E-03	DDT
ENSG00000258791	1.615475824	7.26E-03	LINC00520
ENSG00000120708	1.127276233	7.43E-03	TGFBI
ENSG00000221930	-1.201297465	0.007570904	FAM45BP
ENSG00000235180	1.395575483	0.007601955	LINC00601
ENSG00000118777	1.319790267	0.007622966	ABCG2
ENSG00000071242	-1.133339737	0.007647212	RPS6KA2
ENSG00000130513	-1.114655302	0.007671128	GDF15
ENSG00000251455	2.804307613	0.007864773	AC092611.1
ENSG00000284981	-2.858493641	0.007864773	AC093668.3
ENSG00000198758	-1.162503034	0.007944746	EPS8L3
ENSG00000164690	2.008664317	0.008135331	SHH
ENSG00000186462	2.008664317	0.008135331	NAP1L2
ENSG00000281026	2.008664317	0.008135331	N4BP2L2-IT2
ENSG00000146411	-1.675555506	0.008356463	SLC2A12
ENSG00000277701	3.403657196	0.008390282	AC159540.2
ENSG00000163075	-3.456320152	0.008390282	CFAP221
ENSG00000231177	-3.456320152	0.008390282	LINC00852
ENSG00000253966	-3.456320152	0.008390282	AC008514.2
ENSG00000179388	3.403657196	0.008390282	EGR3
ENSG00000206579	3.403657196	0.008390282	XKR4
ENSG00000164794	3.403657196	0.008390282	KCNV1
ENSG00000225706	3.403657196	0.008390282	PTPRD-AS1
ENSG00000107317	-3.456320152	0.008390282	PTGDS
ENSG00000282787	3.403657196	0.008390282	AL157888.1
ENSG00000265791	3.403657196	0.008390282	AC127024.4
ENSG00000283108	-3.456320152	0.008390282	AC011451.3
ENSG00000236123	-3.456320152	0.008390282	CEACAMP11
ENSG00000279699	-3.456320152	0.008390282	AC004264.2
ENSG00000229598	-3.456320152	0.008390282	PRDX3P1
ENSG00000232692	3.403657196	0.008390282	AP001596.1
ENSG00000271452	2.259001276	0.008458934	AC005034.5
ENSG00000093134	-2.314044362	0.008458934	VNN3
ENSG00000179304	-2.314044362	0.008458934	FAM156B
ENSG00000053438	2.259001276	0.008458934	NNAT
ENSG00000158445	2.259001276	0.008458934	KCNB1
ENSG00000168672	-1.097996853	0.008623853	FAM84B
ENSG00000184110	1.114356873	0.008670514	EIF3C
ENSG00000281344	1.42673266	0.008674877	HELLPAR
ENSG00000210191	1.365219321	0.008684563	MT-TL2
ENSG00000248092	1.582404924	0.008734897	NNT-AS1
ENSG00000108839	-1.52874473	0.008781446	ALOX12
ENSG00000081052	-1.10722327	0.008793983	COL4A4
ENSG00000015532	-1.095613068	0.008871645	XYLT2
ENSG00000126012	-1.088594949	0.008979564	KDM5C
ENSG00000280109	1.900938393	0.00898543	PLAC4
ENSG00000232810	-1.311687893	0.009236593	TNF
ENSG00000272449	1.671364909	0.009270117	AL139246.5
ENSG00000154493	1.271191994	0.009450037	C10orf90
ENSG00000134070	-1.100604024	0.0095489	IRAK2
ENSG00000133805	1.624909714	0.009726153	AMPD3

ENSG00000198944	-1.869575891	0.009730816	SOWAHA
ENSG00000116237	-1.075234306	0.00989054	ICMT
ENSG00000125457	-1.093125363	0.009939606	MIF4GD
ENSG00000130956	1.10815355	0.009942122	HABP4
ENSG00000115041	-1.143593039	0.010002315	KCNIP3
ENSG00000238287	-2.481546376	0.010033723	AL603839.3
ENSG00000110446	-2.481546376	0.010033723	SLC15A3
ENSG00000265190	2.426805621	0.010033723	ANXA8
ENSG00000102683	2.426805621	0.010033723	SGCG
ENSG00000280020	2.426805621	0.010033723	AC116407.3
ENSG00000163082	-1.076480462	0.010149669	SGPP2
ENSG00000114115	1.190478349	0.010178021	RBP1
ENSG00000228589	-1.29136553	0.010190195	SPCS2P4
ENSG00000196562	-1.072615473	0.010193514	SULF2
ENSG00000245317	1.742392343	0.010384446	AC008393.1
ENSG00000106003	-1.60429353	0.010511842	LFNG
ENSG00000138650	1.173902804	0.010517622	PCDH10
ENSG00000100197	1.516783348	0.010850785	CYP2D6
ENSG00000113328	-1.068935145	0.010856414	CCNG1
ENSG00000227063	1.24996394	0.010891877	RPL41P1
ENSG00000084636	-1.201461151	0.010896339	COL16A1
ENSG00000184508	-1.288381745	0.010923125	HDDC3
ENSG00000184100	1.682191153	0.010958385	BRD7P2
ENSG00000163297	-1.073694108	0.011054069	ANTXR2
ENSG00000117758	1.092444615	0.011103686	STX12
ENSG00000249485	1.488363057	0.011159098	RBBP4P1
ENSG00000227234	1.243744253	0.011201183	SPANXB1
ENSG00000171462	1.214110754	0.011440195	DLK2
ENSG00000243902	1.630866928	0.011463391	ELFN2
ENSG00000006118	-1.050042027	0.011772822	TMEM132A
ENSG00000272153	-2.240510774	0.011803031	AL365330.1
ENSG00000250327	-2.240510774	0.011803031	RPSAP70
ENSG00000266235	-2.240510774	0.011803031	MIR3176
ENSG00000214401	-2.240510774	0.011803031	KANSL1-AS1
ENSG00000227375	2.705631538	0.011832427	DLG1-AS1
ENSG00000213492	-2.759801165	0.011832427	NT5C3AP1
ENSG00000185532	2.705631538	0.011832427	PRKG1
ENSG00000122375	-2.759801165	0.011832427	OPN4
ENSG00000266171	-2.759801165	0.011832427	AP001020.3
ENSG00000058091	1.104302447	0.011834838	CDK14
ENSG00000248319	1.241260225	0.012036012	LINC02275
ENSG00000215146	1.153876454	0.012080163	BX322639.1
ENSG00000249839	-1.603628401	0.012302827	AC011330.1
ENSG00000068001	-1.056968048	0.012345	HYAL2
ENSG00000243566	1.137441136	0.012377617	UPK3B
ENSG00000285155	1.763657987	0.012495401	AC092153.1
ENSG00000099399	1.374380288	0.012697517	MAGEB2
ENSG00000202415	-6.036236919	0.012730848	RN7SKP269
ENSG00000270276	-6.036236919	0.012730848	HIST2H4B
ENSG00000228917	6.008634853	0.012730848	AL591806.1
ENSG00000158869	6.008634853	0.012730848	FCER1G
ENSG00000225920	6.008634853	0.012730848	RIMKLBP2
ENSG00000227496	6.008634853	0.012730848	AC099066.2
ENSG00000237685	6.008634853	0.012730848	AL139039.3
ENSG00000232311	-6.036236919	0.012730848	AL512303.1
ENSG00000228521	-6.036236919	0.012730848	AC099552.3
ENSG00000278998	-6.036236919	0.012730848	AC099552.4
ENSG00000147003	-6.036236919	0.012730848	CLTRN
ENSG00000276575	6.008634853	0.012730848	MIR6895
ENSG00000237807	6.008634853	0.012730848	AC022034.1
ENSG00000236896	6.008634853	0.012730848	AL354726.1

ENSG00000256464	6.008634853	0.012730848	YWHABP2
ENSG00000285737	6.008634853	0.012730848	AL138920.1
ENSG00000236671	6.008634853	0.012730848	PRKG1-AS1
ENSG00000243517	6.008634853	0.012730848	AC024940.2
ENSG00000257875	6.008634853	0.012730848	AC089998.3
ENSG00000183032	6.008634853	0.012730848	SLC25A21
ENSG00000259275	-6.036236919	0.012730848	AC087477.2
ENSG00000263235	6.008634853	0.012730848	AC006111.2
ENSG00000258150	-6.036236919	0.012730848	AC133555.3
ENSG00000261838	6.008634853	0.012730848	AC092718.6
ENSG00000166396	-6.036236919	0.012730848	SERPINB7
ENSG00000283078	6.008634853	0.012730848	AL137077.2
ENSG00000268870	-6.036236919	0.012730848	AC008758.5
ENSG00000185837	-6.036236919	0.012730848	HDHD5-AS1
ENSG00000056487	6.008634853	0.012730848	PHF21B
ENSG00000284194	6.008634853	0.012730848	SCO2
ENSG00000228817	6.008634853	0.012730848	BACH1-IT2
ENSG00000237664	-6.036236919	0.012730848	LINC00316
ENSG00000123689	-1.033946543	0.012933962	G0S2
ENSG00000198133	-1.750847962	0.013149317	TMEM229B
ENSG00000177340	-2.070936009	0.01323105	FLJ13224
ENSG00000259341	2.015718931	0.01323105	AC015660.1
ENSG00000112769	1.321641041	0.013256717	LAMA4
ENSG00000137941	1.215764165	0.013277012	TTLL7
ENSG00000139278	1.078655837	0.013305682	GLIPR1
ENSG00000236498	3.279490772	0.013412089	AC107081.2
ENSG00000279191	3.279490772	0.013412089	AC068491.4
ENSG00000114812	-3.332127751	0.013412089	VIPR1
ENSG00000145088	-3.332127751	0.013412089	EAF2
ENSG00000248256	3.279490772	0.013412089	OCIAD1-AS1
ENSG00000182574	3.279490772	0.013412089	Z97985.1
ENSG00000225125	3.279490772	0.013412089	RANP4
ENSG00000165023	3.279490772	0.013412089	DIRAS2
ENSG00000205409	3.279490772	0.013412089	OR52E6
ENSG00000123360	3.279490772	0.013412089	PDE1B
ENSG00000259318	3.279490772	0.013412089	AL356801.1
ENSG00000139988	3.279490772	0.013412089	RDH12
ENSG00000051180	3.279490772	0.013412089	RAD51
ENSG00000266601	-3.332127751	0.013412089	AC018521.6
ENSG00000277287	3.279490772	0.013412089	AL109976.1
ENSG00000141837	3.279490772	0.013412089	CACNA1A
ENSG00000127903	3.279490772	0.013412089	ZNF835
ENSG00000159363	-1.031079004	0.013442734	ATP13A2
ENSG00000281420	-1.693580313	0.013705036	AP001052.1
ENSG00000136155	1.079552209	0.013729422	SCEL
ENSG00000142178	-1.132390556	0.013749441	SIK1
ENSG00000019549	1.036724131	0.013797076	SNAI2
ENSG00000163870	-1.024201226	0.013891559	TPRA1
ENSG00000205572	-1.277277924	0.013926096	SERF1B
ENSG00000078018	1.092886539	0.01403356	MAP2
ENSG00000131979	-1.092384771	0.01407184	GCH1
ENSG00000116299	1.372488857	0.01408992	KIAA1324
ENSG00000107859	-1.428315453	0.01408992	PITX3
ENSG00000254413	-1.644848225	0.01417872	CHKB-CPT1B
ENSG00000129946	-1.15027618	0.014215212	SHC2
ENSG00000225151	-1.247725486	0.014252619	GOLGA2P7
ENSG00000142235	1.145067439	0.014337442	LMTK3
ENSG00000224536	1.889000117	0.014380224	AC096677.1
ENSG00000230006	1.889000117	0.014380224	ANKRD36BP2
ENSG00000175600	1.889000117	0.014380224	SUGCT
ENSG00000257831	1.889000117	0.014380224	AL136418.1

ENSG00000259562	1.889000117	0.014380224	AC090607.2
ENSG00000261253	1.889000117	0.014380224	AC137932.2
ENSG00000267390	1.889000117	0.014380224	AC036176.1
ENSG00000249846	-2.394735203	0.014485426	LINC02021
ENSG00000273447	2.340007138	0.014485426	AC004067.1
ENSG00000216819	2.340007138	0.014485426	TUBB2BP1
ENSG00000242950	2.340007138	0.014485426	ERVW-1
ENSG00000272899	-2.394735203	0.014485426	ATP6V1FNB
ENSG00000055118	-2.394735203	0.014485426	KCNH2
ENSG00000269176	-2.394735203	0.014485426	AP001160.3
ENSG00000173338	2.340007138	0.014485426	KCNK7
ENSG00000279900	2.340007138	0.014485426	AP001767.4
ENSG00000272476	-1.602853185	0.01458372	AL024507.2
ENSG00000259891	-1.602853185	0.01458372	AC107375.1
ENSG00000084073	-1.024681645	0.014686904	ZMPSTE24
ENSG00000157107	-1.031022522	0.014943591	FCHO2
ENSG00000119471	1.022101541	0.015026463	HSDL2
ENSG00000106236	-1.135738326	0.01513802	NPTX2
ENSG00000130768	-1.534116162	0.015229728	SMPDL3B
ENSG00000260948	1.790443993	0.015305394	AL390195.2
ENSG00000173421	1.790443993	0.015305394	CCDC36
ENSG00000158560	1.790443993	0.015305394	DYNC1I1
ENSG00000185630	1.449865641	0.015487289	PBX1
ENSG00000154277	1.072255242	0.015549874	UCHL1
ENSG00000084731	-1.017638611	0.015604154	KIF3C
ENSG00000147650	-1.017200038	0.015614979	LRP12
ENSG00000163697	-1.035382259	0.015696436	APBB2
ENSG00000135414	-1.029896569	0.015710828	GDF11
ENSG00000203791	-1.214071145	0.015736746	EEF1AKMT2
ENSG00000284707	1.711430325	0.016051556	AC079781.5
ENSG00000254815	1.711430325	0.016051556	AP006284.1
ENSG00000187624	1.711430325	0.016051556	C17orf97
ENSG00000266554	1.711430325	0.016051556	LINC01443
ENSG00000164307	-1.025672429	0.01613323	ERAP1
ENSG00000204839	-1.400899363	0.016342315	MROH6
ENSG00000281376	1.329425752	0.016452689	ABALON
ENSG00000168280	2.108003093	0.016492881	KIF5C
ENSG00000065325	2.108003093	0.016492881	GLP2R
ENSG00000269473	-1.702142773	0.016654638	AC012313.8
ENSG00000007402	-1.075317041	0.016665466	CACNA2D2
ENSG00000162654	-1.291530172	0.016874371	GBP4
ENSG00000204604	1.003435652	0.016906063	ZNF468
ENSG00000231924	1.260150153	0.016953164	PSG1
ENSG00000254122	1.251452263	0.016984831	PCDHGB7
ENSG00000132718	1.171615757	0.017109639	SYT11
ENSG00000128536	1.546292169	0.017539069	CDHR3
ENSG00000119938	1.546292169	0.017539069	PPP1R3C
ENSG00000259456	1.546292169	0.017539069	ADNP-AS1
ENSG00000160145	1.085186721	0.017587391	KALRN
ENSG00000197472	1.506674527	0.017860441	ZNF695
ENSG00000235961	1.506674527	0.017860441	PNMA6A
ENSG00000232762	-2.653858269	0.017862218	AL355483.3
ENSG00000172215	2.599707543	0.017862218	CXCR6
ENSG00000010327	-2.653858269	0.017862218	STAB1
ENSG00000244151	2.599707543	0.017862218	AC010973.2
ENSG00000245522	2.599707543	0.017862218	AC026250.1
ENSG00000156968	-2.653858269	0.017862218	MPV17L
ENSG00000267340	2.599707543	0.017862218	AC060780.2
ENSG00000179673	2.599707543	0.017862218	RPRML
ENSG00000104901	2.599707543	0.017862218	DKKL1
ENSG00000231993	2.599707543	0.017862218	EP300-AS1

ENSG00000198435	1.11191207	0.017991567	NRARP
ENSG00000174500	1.945760493	0.018003167	GCSAM
ENSG00000164400	-2.000969329	0.018003167	CSF2
ENSG00000180447	-2.000969329	0.018003167	GAS1
ENSG00000100505	-2.000969329	0.018003167	TRIM9
ENSG00000231595	1.945760493	0.018003167	AC005224.1
ENSG00000102935	1.472220954	0.018121122	ZNF423
ENSG00000226696	1.472220954	0.018121122	LENG8-AS1
ENSG00000112276	-1.031538058	0.018129142	BVES
ENSG00000205084	-1.058499333	0.018663282	TMEM231
ENSG00000151090	1.096263582	0.018762708	THRB
ENSG00000128645	-1.406411119	0.018835279	HOXD1
ENSG00000267904	-1.388903723	0.018902448	AC024075.1
ENSG00000050030	1.231587289	0.018992235	NEXMIF
ENSG00000259590	1.278114951	0.019047235	LINC02244
ENSG00000259342	-1.286775856	0.019047235	AC025580.1
ENSG00000134470	-1.00094369	0.019127018	IL15RA
ENSG00000108448	1.019528121	0.019191123	TRIM16L
ENSG00000184307	-1.019032455	0.019702129	ZDHHC23
ENSG00000126561	-1.055933899	0.019875295	STAT5A
ENSG00000259605	-1.787281855	0.019987884	AC074212.1
ENSG00000277196	-1.157808763	0.020060243	AC007325.2
ENSG00000101333	1.002604685	0.02010665	PLCB4
ENSG00000169129	1.043421875	0.020298794	AFAP1L2
ENSG00000008300	-1.186812354	0.020634313	CELSR3
ENSG00000196951	-2.302364001	0.020952111	SCOC-AS1
ENSG00000256742	2.247650303	0.020952111	AC145422.1
ENSG00000280211	-2.302364001	0.020952111	AC106886.4
ENSG00000285367	2.247650303	0.020952111	AC087564.1
ENSG00000268947	-2.302364001	0.020952111	AC002128.1
ENSG00000178460	1.596177607	0.021096335	MCMDC2
ENSG00000166473	1.596177607	0.021096335	PKD1L2
ENSG00000235750	-1.040579633	0.021200137	KIAA0040
ENSG00000132561	-1.051612531	0.021381169	MATN2
ENSG00000270571	1.545237206	0.021443929	AC007681.1
ENSG00000231574	-1.600841479	0.021443929	LINC02015
ENSG00000276931	1.545237206	0.021443929	AC009041.4
ENSG00000185739	-1.600841479	0.021443929	SRL
ENSG00000267102	1.545237206	0.021443929	AC060766.1
ENSG00000103723	-1.246529335	0.021473972	AP3B2
ENSG00000279069	-1.246529335	0.021473972	AC015813.5
ENSG00000237624	3.143622896	0.021566548	OXCT2P1
ENSG00000272654	3.143622896	0.021566548	AL358472.2
ENSG00000280374	-3.196228767	0.021566548	AC019080.5
ENSG00000229912	3.143622896	0.021566548	AC128709.1
ENSG00000249279	3.143622896	0.021566548	LINC02057
ENSG00000184408	3.143622896	0.021566548	KCND2
ENSG00000228906	3.143622896	0.021566548	AL353804.1
ENSG00000176076	-3.196228767	0.021566548	KCNE5
ENSG00000178125	3.143622896	0.021566548	PPP1R42
ENSG00000121361	3.143622896	0.021566548	KCNJ8
ENSG00000167588	3.143622896	0.021566548	GPD1
ENSG00000173401	3.143622896	0.021566548	GLIPR1L1
ENSG00000257219	3.143622896	0.021566548	LINC02407
ENSG00000257191	3.143622896	0.021566548	AC090709.1
ENSG00000196917	3.143622896	0.021566548	HCAR1
ENSG00000272948	-3.196228767	0.021566548	AP001412.1
ENSG00000270069	1.254704026	0.021631495	MIR222HG
ENSG00000105499	-1.271523361	0.021708179	PLA2G4C
ENSG00000100628	-1.146159279	0.02180232	ASB2
ENSG00000197008	1.26269589	0.021853375	ZNF138

ENSG00000268883	1.432828823	0.021991065	PNMA6B
ENSG00000231683	1.404623132	0.022064739	AL033397.1
ENSG00000267858	-1.460349817	0.022064739	MZF1-AS1
ENSG00000188676	-1.375466605	0.022073746	IDO2
ENSG00000253210	1.319669232	0.022073746	AC040970.1
ENSG00000163491	1.37969242	0.022102993	NEK10
ENSG00000237517	-1.41326062	0.022113468	DGCR5
ENSG00000169554	-1.034115465	0.022299498	ZEB2
ENSG00000111863	1.008850449	0.022475181	ADTRP
ENSG00000268154	-5.846728851	0.022676824	RF00017
ENSG00000237737	5.819187314	0.022676824	DCTN1-AS1
ENSG00000233673	5.819187314	0.022676824	ANAPC1P1
ENSG00000239462	5.819187314	0.022676824	AC091212.1
ENSG00000248590	5.819187314	0.022676824	GLDCP1
ENSG00000171199	5.819187314	0.022676824	OPRPN
ENSG00000251303	5.819187314	0.022676824	CAB39P1
ENSG00000145700	5.819187314	0.022676824	ANKRD31
ENSG00000278685	5.819187314	0.022676824	IQCA1L
ENSG00000248690	5.819187314	0.022676824	HAS2-AS1
ENSG00000231381	5.819187314	0.022676824	RNF2P1
ENSG00000229029	5.819187314	0.022676824	CDCA4P1
ENSG00000269929	-5.846728851	0.022676824	AL158152.1
ENSG00000159712	5.819187314	0.022676824	ANKRD18CP
ENSG00000184956	5.819187314	0.022676824	MUC6
ENSG00000232139	5.819187314	0.022676824	LINC00867
ENSG00000258096	5.819187314	0.022676824	AC025031.2
ENSG00000276390	-5.846728851	0.022676824	AC004241.3
ENSG00000257534	5.819187314	0.022676824	AC023794.4
ENSG00000165899	5.819187314	0.022676824	OTOGL
ENSG00000235097	-5.846728851	0.022676824	LINC00330
ENSG00000102524	5.819187314	0.022676824	TNFSF13B
ENSG00000260046	5.819187314	0.022676824	AL162632.3
ENSG00000252469	-5.846728851	0.022676824	RNU7-160P
ENSG00000260792	5.819187314	0.022676824	LINC02280
ENSG00000259772	-5.846728851	0.022676824	AC012236.1
ENSG00000212766	-5.846728851	0.022676824	EWSAT1
ENSG00000258527	5.819187314	0.022676824	ASB9P1
ENSG00000099769	5.819187314	0.022676824	IGFALS
ENSG00000274505	5.819187314	0.022676824	RF00017
ENSG00000257403	-5.846728851	0.022676824	AC008740.1
ENSG00000261783	-5.846728851	0.022676824	AC009054.2
ENSG00000267659	5.819187314	0.022676824	LINC01482
ENSG00000220008	-5.846728851	0.022676824	LINGO3
ENSG00000267255	-5.846728851	0.022676824	AC011498.3
ENSG00000245598	-5.846728851	0.022676824	DACT3-AS1
ENSG00000185186	-5.846728851	0.022676824	LINC00313
ENSG00000266993	2.02613173	0.023070854	AL050343.1
ENSG00000138741	2.02613173	0.023070854	TRPC3
ENSG00000276840	-2.081144315	0.023070854	PMS2P10
ENSG00000078237	-2.081144315	0.023070854	TIGAR
ENSG00000279765	2.02613173	0.023070854	AC013394.1
ENSG00000243646	-2.081144315	0.023070854	IL10RB
ENSG00000157765	-1.185500256	0.023163897	SLC34A2
ENSG00000254535	1.126456185	0.023239237	PABPC4L
ENSG00000258130	-1.194757465	0.023443495	AC106782.1
ENSG00000233184	1.184472824	0.023589839	AC093157.1
ENSG00000124102	-1.020553405	0.023705297	PI3
ENSG00000103313	-1.14114274	0.024204032	MEFV
ENSG00000223797	-1.927435742	0.024510598	ENTPD3-AS1
ENSG00000237529	-1.927435742	0.024510598	AL137847.2
ENSG00000019505	1.87223601	0.024510598	SYT13

ENSG00000214562	-1.927435742	0.024510598	NUTM2D
ENSG00000250299	1.87223601	0.024510598	MRPS31P4
ENSG00000110318	1.047666262	0.02475408	CEP126
ENSG00000151117	-1.046130169	0.02487568	TMEM86A
ENSG00000238058	1.235792315	0.025107634	AL355574.1
ENSG00000225868	1.24716147	0.025295443	AC016582.1
ENSG00000181798	1.758505265	0.025475297	LINC00471
ENSG00000242268	1.758505265	0.025475297	LINC02082
ENSG00000149256	-1.813833971	0.025475297	TENM4
ENSG00000100100	-1.166422712	0.025639165	PIK3IP1
ENSG00000077935	1.273128326	0.025675336	SMC1B
ENSG00000285278	1.304530072	0.02605029	TFAP2A-AS2
ENSG00000184486	-1.726203138	0.026105331	POU3F2
ENSG00000251893	1.670779793	0.026105331	RF00156
ENSG00000203706	1.366278058	0.02655207	SERTAD4-AS1
ENSG00000213963	1.392330841	0.026682384	AC019080.1
ENSG00000182308	1.392330841	0.026682384	DCAF4L1
ENSG00000116544	-1.599503425	0.026720022	DLGAP3
ENSG00000275494	-1.599503425	0.026720022	AC133552.5
ENSG00000279806	1.543949944	0.026720022	AC018629.1
ENSG00000151687	1.422099768	0.026780029	ANKAR
ENSG00000272221	-1.47777166	0.026780029	AL645933.2
ENSG00000124374	-1.55213629	0.026820067	PAIP2B
ENSG00000268912	-1.55213629	0.026820067	AC012313.5
ENSG00000103269	-1.512088067	0.026831883	RHBDL1
ENSG00000272121	2.485385876	0.02704271	AC006058.3
ENSG00000224614	2.485385876	0.02704271	TNK2-AS1
ENSG00000245067	2.485385876	0.02704271	IGFBP7-AS1
ENSG00000205838	2.485385876	0.02704271	TTC23L
ENSG00000254701	-2.539514583	0.02704271	AC138866.2
ENSG00000266852	-2.539514583	0.02704271	MIR4482
ENSG00000280278	2.485385876	0.02704271	FLJ30679
ENSG00000267141	2.485385876	0.02704271	AC012615.4
ENSG00000271122	-1.194301673	0.02704513	AC018647.2
ENSG00000213801	-1.194301673	0.02704513	ZNF321P
ENSG00000128573	1.215663278	0.028192521	FOXP2
ENSG00000268941	1.1295823	0.028375462	LINC01711
ENSG00000250697	1.225327598	0.02851012	AC010343.3
ENSG00000270504	1.225327598	0.02851012	AL391422.4
ENSG00000177483	1.198916555	0.02851012	RBM44
ENSG00000118507	-1.038279701	0.028596346	AKAP7
ENSG00000248593	1.090615798	0.029332871	DSTNP2
ENSG00000254109	-1.285707631	0.029542889	RBPMS-AS1
ENSG00000163689	1.061094343	0.030048234	C3orf67
ENSG00000133110	1.008260246	0.030067782	POSTN
ENSG00000230424	-2.203671524	0.030348148	AL035413.1
ENSG00000260855	-2.203671524	0.030348148	AL591848.4
ENSG00000229816	2.148974228	0.030348148	DDX50P1
ENSG00000279170	-2.203671524	0.030348148	AL137784.3
ENSG00000218305	-2.203671524	0.030348148	CDC14C
ENSG00000231920	2.148974228	0.030348148	NEBL-AS1
ENSG00000183150	2.148974228	0.030348148	GPR19
ENSG00000255524	-2.203671524	0.030348148	NPIPB8
ENSG00000183018	-2.203671524	0.030348148	SPNS2
ENSG00000239282	-2.203671524	0.030348148	CASTOR1
ENSG00000144642	1.167610268	0.030798047	RBMS3
ENSG00000159753	-1.176281456	0.030798047	CARMIL2
ENSG00000196557	-1.026435985	0.031316925	CACNA1H
ENSG00000115718	1.071564823	0.031698887	PROC
ENSG00000213906	-1.382607523	0.031942348	LTB4R2
ENSG00000159450	1.939333247	0.032292239	TCHH

ENSG00000235092	-1.994333142	0.032292239	ID2-AS1
ENSG00000224184	-1.994333142	0.032292239	MIR3681HG
ENSG00000237732	-1.994333142	0.032292239	AC010980.1
ENSG00000179299	1.939333247	0.032292239	NSUN7
ENSG00000164669	1.939333247	0.032292239	INTS4P1
ENSG00000258311	-1.994333142	0.032292239	BLOC1S1-RDH5
ENSG00000260884	1.939333247	0.032292239	AC009120.3
ENSG00000131477	1.939333247	0.032292239	RAMP2
ENSG00000101331	1.939333247	0.032292239	CCM2L
ENSG00000099338	-1.994333142	0.032292239	CATSPERG
ENSG00000186854	1.07647612	0.032467048	TRABD2A
ENSG00000114739	-1.026317869	0.032556376	ACVR2B
ENSG00000102003	-1.227862164	0.032606579	SYP
ENSG00000106123	-1.236271362	0.033121091	EPHB6
ENSG00000167315	1.409338222	0.033173684	ACAA2
ENSG00000204588	-1.849951934	0.033376343	LINC01123
ENSG00000198237	1.794762311	0.033376343	AC131392.1
ENSG00000229413	1.794762311	0.033376343	AC018638.1
ENSG00000188487	1.794762311	0.033376343	INSC
ENSG00000174600	1.794762311	0.033376343	CMKLR1
ENSG00000157306	-1.849951934	0.033376343	ZFHX2-AS1
ENSG00000197568	1.033346444	0.033490385	HHLA3
ENSG00000228109	1.446133197	0.033533086	MELTF-AS1
ENSG00000166407	-1.501729198	0.033533086	LMO1
ENSG00000158315	1.489760316	0.033832332	RHBDL2
ENSG00000163485	1.688546828	0.033910222	ADORA1
ENSG00000213225	1.688546828	0.033910222	NOC2LP1
ENSG00000280399	1.688546828	0.033910222	AC022497.1
ENSG00000226823	1.688546828	0.033910222	SUGT1P1
ENSG00000137133	1.688546828	0.033910222	HINT2
ENSG00000240476	1.607007307	0.034088382	LINC00973
ENSG00000165272	-1.662423803	0.034088382	AQP3
ENSG00000101162	-1.662423803	0.034088382	TUBB1
ENSG00000250731	-1.662423803	0.034088382	TPM3P6
ENSG00000230061	1.607007307	0.034088382	TRPM2-AS
ENSG00000141294	1.199594738	0.034237201	LRRC46
ENSG00000163013	1.05510045	0.034598295	FBXO41
ENSG00000182359	-1.152084253	0.034718451	KBTBD3
ENSG00000234367	2.993616413	0.034866602	PFN1P3
ENSG00000143199	2.993616413	0.034866602	ADCY10
ENSG00000241635	2.993616413	0.034866602	UGT1A1
ENSG00000246214	-3.046184358	0.034866602	AC022113.1
ENSG00000164512	-3.046184358	0.034866602	ANKRD55
ENSG00000171643	-3.046184358	0.034866602	S100Z
ENSG00000282988	2.993616413	0.034866602	AL031777.3
ENSG00000219470	-3.046184358	0.034866602	AL355802.1
ENSG00000251768	2.993616413	0.034866602	RNA5SP217
ENSG00000232082	-3.046184358	0.034866602	RPS6KA2-IT1
ENSG00000242798	2.993616413	0.034866602	AC073842.2
ENSG00000235806	2.993616413	0.034866602	AF241728.1
ENSG00000269904	2.993616413	0.034866602	MAP2K4P1
ENSG00000253632	2.993616413	0.034866602	AC084026.2
ENSG00000198846	-3.046184358	0.034866602	TOX
ENSG00000254352	2.993616413	0.034866602	AC100854.1
ENSG00000187024	2.993616413	0.034866602	PTRH1
ENSG00000282556	2.993616413	0.034866602	AC068733.3
ENSG00000251194	-3.046184358	0.034866602	AL133330.1
ENSG00000279459	2.993616413	0.034866602	AP001271.2
ENSG00000280367	2.993616413	0.034866602	AP002364.1
ENSG00000120051	-3.046184358	0.034866602	CFAP58
ENSG00000258539	-3.046184358	0.034866602	AC068896.1

ENSG00000236991	2.993616413	0.034866602	EDRF1-AS1
ENSG00000276809	-3.046184358	0.034866602	AL138955.1
ENSG00000126231	-3.046184358	0.034866602	PROZ
ENSG00000139914	2.993616413	0.034866602	FITM1
ENSG00000258708	2.993616413	0.034866602	SLC25A21-AS1
ENSG00000169181	2.993616413	0.034866602	GSG1L
ENSG00000260060	2.993616413	0.034866602	AC009088.1
ENSG00000126861	2.993616413	0.034866602	OMG
ENSG00000172782	2.993616413	0.034866602	FADS6
ENSG00000275542	2.993616413	0.034866602	AC027601.3
ENSG00000273247	1.22264742	0.035481962	AC097376.2
ENSG00000049769	-1.001371985	0.035811734	PPP1R3F
ENSG00000198835	1.061443453	0.036075427	GJC2
ENSG00000185985	-1.081361495	0.036075427	SLITRK2
ENSG00000263826	1.23602327	0.036157187	AC112907.3
ENSG00000250696	1.23602327	0.036157187	AC111000.4
ENSG00000268713	1.23602327	0.036157187	AC005261.3
ENSG00000234155	1.137837837	0.036582079	LINC02535
ENSG00000047936	1.120402249	0.0391281	ROS1
ENSG00000204947	-1.363449992	0.039238489	ZNF425
ENSG00000141854	-1.363449992	0.039238489	MISP3
ENSG00000162415	-1.042207864	0.039302593	ZSWIM5
ENSG00000241484	-1.224468423	0.039663735	ARHGAP8
ENSG00000197837	-1.387921643	0.040101413	HIST4H4
ENSG00000246582	1.178234514	0.040567706	AC100861.1
ENSG00000147234	1.000441145	0.040725658	FRMPD3
ENSG00000049247	-5.628508527	0.040964585	UTS2
ENSG00000228549	-5.628508527	0.040964585	BX284668.2
ENSG00000237950	5.601047283	0.040964585	AL357079.1
ENSG00000231346	-5.628508527	0.040964585	LINC01160
ENSG00000265107	-5.628508527	0.040964585	GJA5
ENSG00000235121	5.601047283	0.040964585	AL645504.1
ENSG00000256671	-5.628508527	0.040964585	LIMS4
ENSG00000182177	-5.628508527	0.040964585	ASB18
ENSG00000225057	-5.628508527	0.040964585	AC012485.1
ENSG00000280571	5.601047283	0.040964585	AC006059.2
ENSG00000221883	-5.628508527	0.040964585	ARIH2OS
ENSG00000240882	-5.628508527	0.040964585	AC063952.2
ENSG00000224097	5.601047283	0.040964585	AC021148.1
ENSG00000145283	5.601047283	0.040964585	SLC10A6
ENSG00000187758	5.601047283	0.040964585	ADH1A
ENSG00000249635	5.601047283	0.040964585	AC109361.1
ENSG00000251155	-5.628508527	0.040964585	SEPT14P4
ENSG00000261083	-5.628508527	0.040964585	LINC02516
ENSG00000249500	5.601047283	0.040964585	LINC01179
ENSG00000164107	5.601047283	0.040964585	HAND2
ENSG00000244073	5.601047283	0.040964585	RPS4XP6
ENSG00000213830	-5.628508527	0.040964585	CFL1P5
ENSG00000250071	5.601047283	0.040964585	AC093214.1
ENSG00000091010	5.601047283	0.040964585	POU4F3
ENSG00000271795	5.601047283	0.040964585	AC011337.1
ENSG00000205318	-5.628508527	0.040964585	GCNT2P
ENSG00000212240	5.601047283	0.040964585	RNU6-930P
ENSG00000238024	5.601047283	0.040964585	DDX39BP2
ENSG00000223865	5.601047283	0.040964585	HLA-DPB1
ENSG00000274259	-5.628508527	0.040964585	SYNGAP1-AS1
ENSG00000271551	-5.628508527	0.040964585	AL355297.4
ENSG00000236039	-5.628508527	0.040964585	AC019117.1
ENSG00000106069	-5.628508527	0.040964585	CHN2
ENSG00000223813	5.601047283	0.040964585	AC007255.1
ENSG00000232729	5.601047283	0.040964585	AC211433.1

ENSG00000158525	5.601047283	0.040964585	CPA5
ENSG00000106410	5.601047283	0.040964585	NOBOX
ENSG00000225037	5.601047283	0.040964585	EIF1AX-AS1
ENSG00000091482	5.601047283	0.040964585	SMPX
ENSG00000186912	5.601047283	0.040964585	P2RY4
ENSG00000226107	-5.628508527	0.040964585	AC004383.1
ENSG00000253934	5.601047283	0.040964585	MRPL49P2
ENSG00000204791	5.601047283	0.040964585	SMPD5
ENSG00000231808	5.601047283	0.040964585	LINC01388
ENSG00000238886	-5.628508527	0.040964585	SNORD121A
ENSG00000154537	-5.628508527	0.040964585	FAM27C
ENSG00000226268	-5.628508527	0.040964585	AC135977.1
ENSG00000124915	5.601047283	0.040964585	DKFZP434K028
ENSG00000244176	5.601047283	0.040964585	AP003733.1
ENSG00000274251	5.601047283	0.040964585	AP003419.2
ENSG00000275484	-5.628508527	0.040964585	AP003419.3
ENSG00000254495	-5.628508527	0.040964585	AP000487.2
ENSG00000225805	5.601047283	0.040964585	DEFB131B
ENSG00000276176	5.601047283	0.040964585	MIR6090
ENSG00000255348	5.601047283	0.040964585	AP001775.2
ENSG00000277479	-5.628508527	0.040964585	AL022345.3
ENSG00000130038	5.601047283	0.040964585	CRACR2A
ENSG00000212124	5.601047283	0.040964585	TAS2R19
ENSG00000197870	-5.628508527	0.040964585	PRB3
ENSG00000275769	5.601047283	0.040964585	AC068792.1
ENSG00000201563	-5.628508527	0.040964585	RF00019
ENSG00000257896	-5.628508527	0.040964585	AC093012.1
ENSG00000248265	5.601047283	0.040964585	FLJ12825
ENSG00000258344	-5.628508527	0.040964585	AC078778.2
ENSG00000273987	5.601047283	0.040964585	AC121761.2
ENSG00000258230	5.601047283	0.040964585	AC063950.1
ENSG00000158113	5.601047283	0.040964585	LRRC43
ENSG00000272158	-5.628508527	0.040964585	AL139022.2
ENSG00000258793	-5.628508527	0.040964585	AL355102.4
ENSG00000182218	-5.628508527	0.040964585	HHIPL1
ENSG00000259211	-5.628508527	0.040964585	AC013356.2
ENSG00000274297	5.601047283	0.040964585	AC009269.5
ENSG00000117971	-5.628508527	0.040964585	CHRNB4
ENSG00000259244	5.601047283	0.040964585	AC048382.2
ENSG00000260337	-5.628508527	0.040964585	AC091544.4
ENSG00000275445	-5.628508527	0.040964585	AC092119.3
ENSG00000178573	5.601047283	0.040964585	MAF
ENSG00000183914	5.601047283	0.040964585	DNAH2
ENSG00000235554	5.601047283	0.040964585	AC005822.1
ENSG00000266129	5.601047283	0.040964585	SRP68P1
ENSG00000276054	5.601047283	0.040964585	AC243654.3
ENSG00000280295	5.601047283	0.040964585	AC099811.6
ENSG00000221044	5.601047283	0.040964585	RF00012
ENSG00000265542	5.601047283	0.040964585	AC015845.2
ENSG00000267009	-5.628508527	0.040964585	AC007780.1
ENSG00000267203	5.601047283	0.040964585	SNRPGP4
ENSG00000204282	-5.628508527	0.040964585	TNRC6C-AS1
ENSG00000267108	-5.628508527	0.040964585	AP001029.1
ENSG00000283458	5.601047283	0.040964585	AC011139.1
ENSG00000263916	-5.628508527	0.040964585	AC100778.2
ENSG00000274322	5.601047283	0.040964585	AL136531.2
ENSG00000124232	5.601047283	0.040964585	RBPJL
ENSG00000124097	5.601047283	0.040964585	HMGB1P1
ENSG00000130812	-5.628508527	0.040964585	ANGPTL6
ENSG00000196361	5.601047283	0.040964585	ELAVL3
ENSG00000279044	5.601047283	0.040964585	AC007787.2

ENSG00000225067	5.601047283	0.040964585	RPL23AP2
ENSG00000267033	5.601047283	0.040964585	AC020911.1
ENSG00000225872	5.601047283	0.040964585	LINC01529
ENSG00000270760	5.601047283	0.040964585	AD001527.1
ENSG00000267375	5.601047283	0.040964585	AC008649.2
ENSG00000197380	5.601047283	0.040964585	DACT3
ENSG00000104870	5.601047283	0.040964585	FCGRT
ENSG00000221039	5.601047283	0.040964585	MIR1286
ENSG00000235513	-5.628508527	0.040964585	AL035681.1
ENSG00000278996	5.601047283	0.040964585	FP671120.1
ENSG00000210117	5.601047283	0.040964585	MT-TW
ENSG00000169862	1.360637028	0.040993521	CTNND2
ENSG00000169067	1.360637028	0.040993521	ACTBL2
ENSG00000159784	1.360637028	0.040993521	FAM131B
ENSG00000175356	1.360637028	0.040993521	SCUBE2
ENSG00000148600	-1.416268414	0.040993521	CDHR1
ENSG00000225721	2.361219452	0.041032031	AL592166.1
ENSG00000228237	2.361219452	0.041032031	EFCAB14-AS1
ENSG00000224680	-2.415322182	0.041032031	PLA2G12AP1
ENSG00000162779	2.361219452	0.041032031	AXDND1
ENSG00000235586	-2.415322182	0.041032031	AC011247.2
ENSG00000188687	2.361219452	0.041032031	SLC4A5
ENSG00000074317	-2.415322182	0.041032031	SNCB
ENSG00000180316	-2.415322182	0.041032031	PNPLA1
ENSG00000232759	2.361219452	0.041032031	AC002480.1
ENSG00000232104	-2.415322182	0.041032031	RFX3-AS1
ENSG00000274943	2.361219452	0.041032031	AC079684.1
ENSG00000254718	-2.415322182	0.041032031	AL157756.1
ENSG00000184206	2.361219452	0.041032031	GOLGA6L4
ENSG00000242622	-1.244590774	0.041535125	AC092910.3
ENSG00000266302	1.110934263	0.041652925	AC098850.3
ENSG00000069188	-1.065960352	0.041706837	SDK2
ENSG00000168874	-1.449496942	0.041903977	ATOH8
ENSG00000248890	1.393905535	0.041903977	HHIP-AS1
ENSG00000099840	1.393905535	0.041903977	IZUMO4
ENSG00000134668	1.051712667	0.042499767	SPOCD1
ENSG00000002746	1.051712667	0.042499767	HECW1
ENSG00000125089	-1.067056829	0.042499767	SH3TC1
ENSG00000253394	-1.488998742	0.042814164	LINC00534
ENSG00000256092	-1.488998742	0.042814164	AC137767.1
ENSG00000247982	1.433455544	0.042814164	LINC00926
ENSG00000090932	-1.488998742	0.042814164	DLL3
ENSG00000118160	-1.488998742	0.042814164	SLC8A2
ENSG00000174567	-1.178698436	0.043639812	GOLT1A
ENSG00000163803	1.213559454	0.043684225	PLB1
ENSG00000232775	-1.536755599	0.043692991	BMS1P22
ENSG00000117281	2.043050233	0.043991311	CD160
ENSG00000135773	-2.097728628	0.043991311	CAPN9
ENSG00000138028	-2.097728628	0.043991311	CGREF1
ENSG00000164509	2.043050233	0.043991311	IL31RA
ENSG00000258521	-2.097728628	0.043991311	AL157871.2
ENSG00000140832	2.043050233	0.043991311	MARVELD3
ENSG00000276523	2.043050233	0.043991311	AC025287.3
ENSG00000108958	-2.097728628	0.043991311	AC130689.1
ENSG00000075043	-2.097728628	0.043991311	KCNQ2
ENSG00000205790	-2.097728628	0.043991311	DPP9-AS1
ENSG00000274425	-2.097728628	0.043991311	AC114271.1
ENSG00000160207	1.056534792	0.044232085	HSF2BP
ENSG00000242960	-1.069451665	0.044232085	FTH1P23
ENSG00000134321	-1.59569394	0.044489089	RSAD2
ENSG00000172935	1.540284941	0.044489089	MRGPRF

ENSG00000261226	1.540284941	0.044489089	AC092384.3
ENSG00000255423	1.22819345	0.044879015	EBLN2
ENSG00000112333	1.22819345	0.044879015	NR2E1
ENSG00000272695	1.22819345	0.044879015	GAS6-DT
ENSG00000054277	1.100755623	0.045117399	OPN3
ENSG00000279289	1.100755623	0.045117399	AL136164.3
ENSG00000229931	1.615022344	0.045117465	AL137003.1
ENSG00000123496	1.615022344	0.045117465	IL13RA2
ENSG00000261351	1.615022344	0.045117465	AC116913.1
ENSG00000280033	1.615022344	0.045117465	AC116407.4
ENSG00000232860	1.846976412	0.045202178	SMG7-AS1
ENSG00000251685	1.846976412	0.045202178	UGT2B27P
ENSG00000246090	1.846976412	0.045202178	AP002026.1
ENSG00000079435	-1.90196194	0.045202178	LIPE
ENSG00000223658	-1.768069282	0.045435959	C1GALT1C1L
ENSG00000235781	-1.768069282	0.045435959	LINC02569
ENSG00000105877	1.712890948	0.045435959	DNAH11
ENSG00000229207	1.712890948	0.045435959	SERPINH1P1
ENSG00000108001	-1.768069282	0.045435959	EBF3
ENSG00000267226	1.712890948	0.045435959	AC104971.1
ENSG00000179772	1.712890948	0.045435959	FOXS1
ENSG00000237118	-1.768069282	0.045435959	CYP2F2P
ENSG00000229676	1.005351737	0.045783952	ZNF492
ENSG00000006837	1.244720227	0.046163285	CDKL3
ENSG00000229419	-1.135102757	0.046223025	RALGAPA1P1
ENSG00000188763	-1.073650979	0.047267958	FZD9
ENSG00000271895	-1.319224346	0.04754413	AL109811.3
ENSG00000177432	-1.008576416	0.04808137	NAP1L5
ENSG00000162009	-1.209928755	0.048597149	SSTR5
ENSG00000271425	-1.143999586	0.048686192	NBPF10
ENSG00000185839	-1.340807179	0.049028188	AL035411.1
ENSG00000231982	1.285145821	0.049028188	AC112907.2
ENSG00000242220	1.285145821	0.049028188	TCP10L
ENSG00000124593	-1.076957931	0.049653713	AL365205.1

Table S6. Clinical characteristics of ovarian cancer patients.

Characteristics	Ovarian cancer patients (*n* = 147)
Age, year	
Median (range)	51 (20-75)
Grander, *n* (%)	
Female	147 (100)
Male	0 (0)
Tumor size, cm (%)	
⩽ 5	30 (20.4)
> 5	117 (79.6)
